# Memorias del XXXIV Congreso Anual de la Sociedad Mexicana de Neurología Pediátrica A.C.

**DOI:** 10.31083/RN49601

**Published:** 2026-07-21

**Reporte de un Caso Miopatía Relacionada con el Receptor de Ryanodina Tipo 1 (RYR1)**

Arias Méndez Antonio Jesús^1^, González Medina Mario^1^

^1^Unidad Médica de Neurología, Hospital Regional de Alta Especialidad del Niño Dr. Rodolfo Nieto Padrón, 86030 Villahermosa, México

**Introducción**: La MRRR1 es causada por mutaciones en el gen *RYR1*, que codifica el receptor de rianodina tipo, crucial para la liberación de calcio en las células musculares esqueléticas. Las alteraciones en este proceso afectan la contracción muscular, provocando hipotonía, miopatías congénitas y otras disfunciones neuromusculares desde el nacimiento. Es una miopatía infrecuente donde el diagnóstico temprano y un manejo multidisciplinario son esenciales, ya que el pronóstico varía según la severidad de las manifestaciones clínicas. **Objetivo**: Presentar un caso clínico de miopatía neonatal infrecuente con mutación en el gen *RYR1*, destacando la importancia del diagnóstico temprano y el enfoque multidisciplinario en su manejo. **Resumen Clínico del Caso Con Resultado de Estudios, Tratamiento Instaurado y Evolución**: El paciente presentó hipotonía severa, reflejos primitivos ausentes y artrogriposis generalizada. Los estudios de imagen fueron normales, pero el análisis genético confirmó la mutación en el gen *RYR1*, diagnosticando miopatía congénita. Los resultados de laboratorio mostraron niveles elevados de CPK y lactato, indicando disfunción muscular. El seguimiento mostró una evolución tórpida con respuestas motoras limitadas. **Conclusiones**: El diagnóstico precoz de la miopatía congénita por mutación en el gen *RYR1* es fundamental para un manejo adecuado. Aunque no existe un tratamiento específico para la enfermedad, un enfoque multidisciplinario que incluya el uso de fármacos como salbutamol, piridostigmina y N-acetilcisteína, es esencial para mejorar la calidad de vida y optimizar el manejo clínico.

**Palabras Clave:**

miopatía congénita; RYR1 (MRRR1); hipotonía neonatal; enfermedades neuromusculares; diagnóstico genético

**Leucoencefalopatía Tóxica Asociada a Metotrexato Como Simulador de Evc: Reporte de Caso**

Medellín López Haideé Deyanira^1^, Rogel Cuevas Josué^1,2^

^1^Hospital Regional de Alta Especialidad del Niño Dr. Rodolfo Nieto Padrón, 86100 Villahermosa, Tabasco, México

^2^Universidad Nacional Autónoma de México (UNAM), 04510 Ciudad de México, México

**Introducción**: El metotrexato es un fármaco análogo del ácido fólico ampliamente utilizado en el tratamiento de enfermedades hematooncológicas. Su uso puede ocasionar la aparición de múltiples efectos adversos entre los que se encuentran aquellos relacionados con la presencia de toxicidad neurológica, que puede presentarse de forma aguda, subaguda o crónica. **Objetivo**: Describir el caso de un paciente con leucoencefalopatía tóxica secundaria a quimioterapia. **Resumen Clínico del Caso Con Resultado de Estudios, Tratamiento Instaurado y Evolución**: Paciente femenino de 15 años con diagnóstico de Leucemia linfoblástica Aguda B en protocolo de quimioterapia con 6-mercaptopurina y metotrexato con administración de este último 10 días previos al padecimiento. Ingresa con 12 horas de evolución con focalidad neurológica con hemiparesia faciocorporal izquierda sin otro acompañante, se realiza RMN cerebral con secuencias de protocolo STROKE en la que se identifican imágenes hiperintensas en T2, subcorticales, confluentes, con restricción verdadera a la difusión, en relación con edema citotóxico, hallazgos sugestivos de leucoencefalopatía tóxica. Se revalora a la paciente a las 24 horas de evolución con mejoría de la sintomatología con remisión de la parálisis facial y mejoría de la fuerza en hemicuerpo izquierdo. **Conclusiones**: Presentamos el caso de una paciente con neurotoxicidad subaguda es aquella que ocurre típicamente entre los 2 a 14 días posteriores a la administración de metotrexato y puede manifestarse con una amplia gama de síntomas neurológicos, en el caso de esta paciente se presentó a los 10 días de su administración con focalidad neurológica motora que remitió por completo.

**Palabras Clave:**

enfermedad vascular cerebral; leucoencefalopatía tóxica; metotrexato; neurotoxicidad

**Leucodistrofia de Alexander GFAP Variante en TOE1 Autosómica Recesiva: Reporte de Caso**

Ruíz González Samantha^1^, Sánchez Rojo Fernando^1^

^1^Servicio de Pediatría, Hospital General de La Piedad, 59350 La Piedad de Cabadas, México

**Introducción**: La enfermedad de Alexander (EA), es un raro trastorno neurodegenerativo de la sustancia blanca del sistema nervioso central (SNC) (leucodistrofia), con formación de fibras de Rosenthal. La EA se presenta con datos como macrocefalia, retraso motor, cognitivo, convulsiones, ataxia, disfagia, disartria, mioclonus palatal y disfunción autonómica. Existen cinco criterios principales por resonancia magnética (RM): Atrofia de la sustancia blanca frontal; Borde periventricular con hipointensidad en T2 e hiperintensidad en T1; Anomalías de los ganglios basales y talámicos (Hipointensidad en T2); Tronco encefálico (médula y el mesencéfalo); Y reforzamiento del contraste de uno o más de los siguientes: Halo periventricular, sustancia blanca frontal, quiasma óptico, fórnix, ganglios basales, tálamo, núcleo dentado y tronco cerebral. Las pruebas genéticas confirman el diagnóstico con identificación de GFAP (proteína ácida fibrilar glial). **Objetivo**: Describir el reporte de caso de un paciente con Leucodistrofia de Alexander en edad pediátrica. **Resumen Clínico del Caso Con Resultado de Estudios, Tratamiento Instaurado y Evolución**: Revisión de expediente clínico y descripción de hallazgos. Paciente masculino de 6 meses de edad que presenta retraso psicomotor (sostén cefálico, rodamiento, sedestación), algunas vocalizaciones, perímetro cefálico de 43 cm, percentil 50, tendencia a la mirada central y aversiva hacia abajo (tipo sol naciente), nistagmos horizontal evocado, no dirige la mirada, inicialmente hipotónico global, sin focalizaciones, REMSS +++/+++, en estimulación temprana. Tomografía simple de cráneo con hiperdensidad de sustancia blanca, centros semiovales y periventriculares bilateral, se realiza RM de cráneo, con datos de leucodistrofia de la sustancia blanca bilateral supra e infratentorial, involucrando tallo cerebral, medula cervical, discreta atrofia cortical anterior y a nivel pontocerebebeloso. Sistema ventricular conservado. Los hallazgos específicos de imágenes de RM apuntaban al diagnóstico de EA, que se confirmó al encontrar una mutación patógena del gen *GFAP* variante en TOE1 c.668del (p.Pro223GFinfs*22), asociación autosómica recesiva pontocerebelar hipoplásica. Potenciales evocados sin alteraciones, Electroencefalograma con puntas frontales izquierdas. **Conclusiones**: Las leucodistrofias son entidades poco comunes, la EA extremadamente rara, normalmente en infantes, con destrucción progresiva de la sustancia blanca cerebral, deben sospecharse en caso de presentar detención y regresión del neurodesarrollo, es crucial la identificación temprana, con el propósito de iniciar manejo paliativo y asesoramiento genético familiar.

**Palabras Clave:**

neurodegenerativo; neurodesarrollo; leucodistrofia; enfermedad de Alexander; gen *GFAP*; resonancia magnética

**Evento Vascular Cerebral Isquémico en Paciente con Sospecha de Homocistinuria, Reporte de un Caso**

Ibarra Delgado Alberto^1^, Silva Ramírez Martin Arturo^1^, De la Fuente Silva Fabiola Marycruz^1^, Cebada López Flora^1^

^1^Instituto Mexicano del Seguro Social, UMAE Hospital General, “Dr. Gaudencio González Garza” La Raza, 02990 Ciudad de México, México

**Introducción**: La Homocistinuria es un error innato poco frecuente del metabolismo de la metionina y sus metabolitos con un patrón de herencia autosómico recesivo. Esta enfermedad causa un acumulo anormal de homocisteína en los tejidos, afectando principalmente al sistema óseo, ocular y vascular, este último produciendo eventos vasculares cerebrales de tipo isquémico. Clínicamente los pacientes presentan miopía severa, anomalías esqueléticas (dolicostenomelia, escoliosis, pectus excavatum, entre otras), retraso del desarrollo o discapacidad intelectual y eventos vasculares cerebrales de tipo isquémico. El diagnóstico se establece con niveles de homocisteína plasmática total o mediante la identificación de variantes patogénicas. **Objetivo**: Describir la presentación clínica y abordaje de un paciente pediátrico con sospecha diagnostica de Homocistinuria. **Resumen Clínico del Caso Con Resultado de Estudios, Tratamiento Instaurado y Evolución**: Adolescente de 16 años, se diagnosticó con displasia congénita de cadera a los 8 meses, a los 15 años diagnóstico de discapacidad intelectual (coeficiente intelectual de 81), a los 16 años diagnóstico de escoliosis. Inicia a los 16 años con cefalea temporal bilateral punzante 7/10 con incremento paulatino a 9/10, posteriormente parálisis facial central y hemiplejia derechas. Durante su abordaje se identificó una trombosis aguda de arteria carótida interna izquierda con evento vascular cerebral, así como trombosis aguda oclusiva de tercio distal de la vena safena mayor en pierna derecha. Dentro del abordaje se descartaron causas cardiacas, reumatológicas e infecciosas que pudieran condicionar el cuadro isquémico a nivel cerebral, sin embargo, debido a la presencia de discapacidad intelectual, alteraciones óseas y el evento vascular cerebral y trombótico a nivel de extremidad inferior derecha fueron sugestivos de homocistinuria por lo que solicitó exoma. **Conclusiones**: Este es un padecimiento poco frecuente, pero de importancia clínica en la población pediátrica debido a su amplio espectro de manifestaciones clínicas y riesgo de recurrencia de evento vascular cerebral isquémico, en el caso de nuestra paciente continúa con abordaje, aún pendiente el resultado del exoma, sin embargo, cumple con las características clínicas para sospecha diagnostica.

**Palabras Clave:**

homocistinuria; EVC; discapacidad intelectual; escoliosis

**Distrofia Muscular de Cinturas. Una Enfermedad Multigénica. Reporte de Caso**

María Fernanda Pérez-Álvarez^1^, Omar Alejandro Martínez-Fernández^1^, Flora Cebada-López^1^

^1^Instituto Mexicano Del Seguro Social, UMAE Hospital General “Dr. Gaudencio González Garza” Centro Médico Nacional La Raza, 02990 Ciudad de México, México

**Introducción**: La Distrofia Muscular de cinturas, abarca un grupo heterogéneo de miopatías, asociada a más de 30 genes directamente, clasificada en 2 categorías dependiendo su tipo de herencia, con inicio de manifestaciones a cualquier edad. Existe compromiso principalmente en musculo esquelético, provocando debilidad progresiva de predominio en cintura pélvica y escapular secundario a perdida de fibras musculares. Algunas variantes presentan afectación cardiaca. Se acompaña de elevación de creatinina quinasa y alteraciones en resonancia magnética (sustitución grasa de los músculos). El diagnóstico se confirma mediante estudio genético y/o biopsia muscular. Se debe realizar diagnóstico diferencial dad su similitud con entidades tratables como la enfermedad de POMPE. El tratamiento se basa en rehabilitación, dado que no existe tratamiento curativo. Actualmente se encuentran en curso múltiples ensayos clínicos sobre terapias génicas que pudieran ser prometedores en cuanto a tratamiento. **Objetivo**: Presentar un caso de distrofia muscular de cinturas, enfatizando en el impacto del asesoramiento genético en el manejo del paciente neurológico. **Resumen Clínico del Caso Con Resultado de Estudios, Tratamiento Instaurado y Evolución**: Femenino de 7 años, sin antecedentes de relevancia. Inicia los 8 meses con dificultades para gatear, detectando a hipotonía, posteriormente con dificultades para consolidar la marcha. Captada por nuestro hospital en 2023, con cariotipo 46 XX, estudios complementarios mostraban elevación de creatinina quinasa, electromiografía con patrón miopático y estudio enzimático negativo para enfermedad de POMPE. Clínicamente presenta hipotonía, hipomimia facial, disminución de fuerza muscular de predominio proximal, hiporreflexia y artrogriposis. Se realiza exoma externo reportando variante patogénica homocigoto en gen *FKRP*, confirmando diagnóstico con base a la correlación clínica. Actualmente en seguimiento multidisciplinario, sin progresión de debilidad ni compromiso cardiológico o restrictivo pulmonar. **Conclusiones**: El tema genético se ha impulsado en los últimos años, logrando diagnosticar y/o descubrir nuevos padecimientos. Es trascendental el diagnóstico oportuno de este tipo de distrofias dada su similitud con enfermedad metabólicas tratables, más aún, para brindar asesoramiento genético a las familias.

**Palabras Clave:**

distrofia muscular; gen *FKRP*

**Hallazgos Clínicos en Esclerosis Tuberosa: Serie de Casos en Pediatría**

Sobrevilla Ponce Anali Guadalupe^1^, Cantú Salinas Adriana Carlota^1^, Carrión García Ana Luisa^1^, Vázquez Fuentes Salvador^1^, Chávez Luévanos Beatriz Eugenia^1^

^1^Hospital Universitario Dr. José Eleuterio González, 64460 Monterrey, México

**Introducción**: Se trata de un trastorno neurocutáneo, hereditario que involucra varios órganos y sistemas. Su expresión varía considerablemente, se describe de manera clásica con la triada de Vogt: convulsiones, discapacidad intelectual y angiofibromas faciales, los genes afectados son *TSC1*, *9q34* (hamartina) o *TSC2*, *16p13* (tuberina). Los pacientes con mutaciones TSC2 sufren de síntomas más severos que aquellos con mutaciones en TSC1, presentando tubérculos corticales, angiomiolipomas renales, hamartomas retinianos, y angiofibromas faciales, se observa también epilepsia, farmacorresistente. **Objetivo**: Comparar las características clínicas e imagenológicas en cuatro pacientes con diagnóstico de esclerosis tuberosa tratados en un hospital de tercer nivel durante el año 2024. **Resumen Clínico del Caso Con Resultado de Estudios, Tratamiento Instaurado y Evolución**: Se realizó un estudio descriptivo, transversal, retrospectivo, tratándose de una investigación sin riesgo al ser una investigación documental retrospectiva, que no requirió la realización de ninguna intervención, solamente requiriéndose la revisión de expedientes clínicos. Se recabaron cuatro casos de pacientes con diagnóstico de Complejo esclerosis tuberosa (CET).75% de los pacientes son del sexo masculino (n = 3), con edades comprendidas entre 1 a 13 años, con edad media de diagnóstico de 7 meses, solo uno de ellos pudo ser diagnosticado in útero por presencia de rabdomioma cardiaco, Ninguno contaba con antecedente heredofamiliar, los 4 pacientes presentaron alteración dermatológica caracterizada por angiofibromas faciales, 2 de ellos además placa de Chagrin; 2 de ellos con diagnóstico de discapacidad intelectual, 2 de ellos con rabdomiomas intra cardiacos que no alteran la función; 2 cuentan con quistes renales, 1 de ellos con hipertensión arterial nefrogénica. 3 de ellos cumplieron criterios clínicos para diagnóstico de CET, 1 de ellos con exoma genético confirmando alteración en el gen *TSC2*. 3 de ellos presentan epilepsia multitratada y refractaria a tratamiento farmacológico de los cuales una paciente fue sometida a lesionectomía para extracción del tuber de mayor tamaño y mejoró considerablemente la epilepsia, en ninguno de ellos se confirmó tumor de células gigantes subependimario (SEGA) o hamartomas retinianos. **Conclusiones**: El perfil clínico de CET es variable, por lo que es importante conocer sus criterios diagnósticos y comorbilidades para el adecuado tratamiento y seguimiento de los pacientes que cuentan con este diagnóstico.

**Palabras Clave:**

complejo esclerosis tuberosa; angiofibromas faciales; epilepsia farmacorresistente

**Desafíos Diagnósticos en el Guillain-Barré Atípico: Caso Clínico en una Niña de 7 años**

Jacqueline D. Lucio-Escamilla^1^, Cantú Salinas Adriana Carlota^1^, Carrión García Ana Luisa^1^, Vázquez Fuentes Salvador^1^, De la Garza Pineda Oscar^1^, Chávez Luévanos Beatriz Eugenia^1^

^1^Servicio de Neurología, Hospital Universitario “Dr. José Eleuterio González”, Universidad Autónoma de Nuevo León, 64460 Monterrey, México

**Introducción**: El síndrome de Guillain-Barré (SGB) es una polirradiculoneuropatía autoinmune caracterizada por debilidad progresiva y arreflexia. Existen variantes típicas y atípicas; sus variantes atípicas pueden retrasar el diagnóstico. Ante un paciente con cuadro clínico de debilidad muscular, con oftalmoplejía, alteraciones sensitivas, alteración del estado de conciencia, ataxia, respuesta extensora plantar bilateral, es importante sospechar SGB atípico. Su reconocimiento temprano permite un manejo oportuno, mejorando el pronóstico y la recuperación funcional ante este diagnóstico. **Objetivo**: Describir en detalle un caso clínico de Guillain-Barré atípico en una niña de 7 años, resaltando las manifestaciones clínicas poco comunes que dificultaron su diagnóstico, y subrayando la relevancia del diagnóstico temprano para implementar una intervención terapéutica adecuada, lo que mejora el pronóstico funcional del paciente. **Resumen Clínico del Caso Con Resultado de Estudios, Tratamiento Instaurado y Evolución**: Femenina de 7 años con antecedente de infección gastrointestinal previa. Inició con dolor 4/10 en la escala análoga del dolor, debilidad en miembro pélvico derecho que se propaga contralateralmente, con progresión ascendente acompañado de hiporreflexia y respuesta plantar extensora bilateral. En LCR con disociación albúmino-citológica y en los ECN con polirradiculoneuropatía desmielinizante, se manejó con inmunoglobulina a dosis de 1 g/kg/día, egresándose con un puntaje de 2 de la Guillain Barre Disability Score (GDS). **Conclusiones**: La variabilidad clínica del Guillain-Barré en pediatría como el inicio asimétrico o presencia de signos piramidales, puede retrasar su diagnóstico. Una correlación de la clínica con estudios neurofisiológicos y LCR permite el diagnóstico oportuno y una intervención terapéutica temprana mejorando el pronóstico funcional del paciente.

**Palabras Clave:**

Guillain-Barré atípico; polirradiculoneuropatía desmielinizante; diagnóstico temprano neuropatía periférica; inmunoglobulina intravenosa; disociación albúmino-citológica; estudio neurofisiológico

**Experiencia de Tratamiento con Anticuerpo Monoclonal en Neuritis Óptica Idiopática Recurrente en una Adolescente**

Emmanue Martínez-Fuentes^1^, Lennin A Marquez-Villaseñor^1^, Adriana C Cantú-Salinas^1^, Fernanda Flores-Alfaro^1^, Sergio Saldívar-Dávila^1^, Jibran Mohamed-Noriega^2^, José L. De León-Guerra^1^, Jesús O. Vargas-de-la-Rosa^1^, Beatriz E. Chávez-Luevanos^1^

^1^Servicio de Neurología, Hospital Universitario “Dr. José Eleuterio González”, Universidad Autónoma de Nuevo León (UANL), 64460 Monterrey, México

^2^Departamento de Oftalmología, Hospital Universitario, Facultad de Medicina, Universidad Autónoma de Nuevo León (UANL), 64460 Monterrey, México

**Introducción**: La neuritis óptica (NO), caracterizada por la tríada: disminución de la agudeza visual, discromatopsias y dolor periocular puede asociarse a enfermedades desmielinizantes como la Esclerosis Múltiple (EM), el Trastorno del Espectro de la Neuromielitis Óptica (NMOSD), la Enfermedad Asociada a anticuerpos contra la Glucoproteína de Oligodendrocitos de Mielina (MOGAD), entre otras. La NO comparte el edema del disco óptico como signo con otras patologías como las neuropatías ópticas isquémicas. En la edad pediátrica las NO son más frecuentemente anteriores y presentan algunas de las características históricamente descritas como atípicas. La Tomografía de Coherencia Óptica (OCT) y pruebas de Campos Visual (CV) ofrecen información que permite evaluar la gravedad, monitorizar eficacia y resolución de ataques. **Objetivo**: Describir la evolución clínica y paraclínica, previa y posteriormente al uso de rituximab en una adolescente con NO idiopática recurrente. **Resumen Clínico del Caso Con Resultado de Estudios, Tratamiento Instaurado y Evolución**: Femenina de 12 años sin antecedentes relevantes, inicia en enero de 2024 con NO bilateral de predominio derecho, descartándose durante su abordaje EM, NMOSD, MOGAD, Lupus Eritematoso Sistémico e infección por M. tuberculosis. La resonancia magnética demuestra cambios inflamatorios en ambos nervios ópticos con predominio derecho. Inicia tratamiento con metilprednisolona (MP) 1 g/día durante cinco días, obteniendo respuesta parcial, agregándose Inmunoglobulina Intravenosa con mejoría clínica y es egresada con prednisona (PRED) (1 mg/kg/día). En marzo 2024, presenta la primera recaída afectando al ojo izquierdo, tratándose con MP, azatioprina (AZA) (2,5 mg/kg/día) y PRED. Evoluciona favorablemente durante 5 meses y en agosto de 2024 al descender la dosis de PRED, presenta la segunda recaída ahora en el ojo derecho readministrándose el tratamiento previamente descrito. En septiembre 2024, ocurre la tercera recaída en el ojo izquierdo, iniciándose rituximab (375 mg/SCm2/dosis). **Conclusiones**: Tras 6 meses de tratamiento, la paciente no ha presentado recaídas. La agudeza visual se ha mantenido en 20/25 bilateralmente, sin discromatopsias ni dolor periocular. Los estudios de seguimiento por OCT muestran disminución del edema y las pruebas CV recuperación campimétrica.

**Palabras Clave:**

neuritis óptica; rituximab; enfermedad desmielinizante; pediatría

**¿Pueden los Esteroides Inducir Reducción del Timo en Miastenia Gravis? Presentación de Caso y Revisión de la Literatura**

Marco Antonio Vidal-Peregrina^1^, Fabiola Marycruz De la Fuente-Silva^1^, Salvador Jiménez-Mejinez^1^, Martín Arturo Silva-Ramírez^1^, Flora Cebada-López^1^

^1^Instituto Mexicano del Seguro Social, UMAE Hospital General “Dr. Gaudencio González Garza” Centro Médico Nacional La Raza, 02990 Ciudad de México, México

**Introducción**: El timo tiene un rol importante en la Miastenia Gravis (MG). Existen escasos reportes de reducción o regresión del timo secundario a uso de esteroides. **Objetivo**: Presentación de caso de reducción del timo secundario a esteroides en MG Juvenil. **Resumen Clínico del Caso Con Resultado de Estudios, Tratamiento Instaurado y Evolución**: Reporte de caso: Masculino de 8 años, originario de Yucatán, MG Generalizada Osserman III desde hace 3 años, tratado con Piridostigmina 4,6 mg/kg/d y Prednisona 1 mgkgd con mejoría, disminuyen Prednisona, reiniciando con sintomatología aumentando Piridostigmina, suspenden esteroide por aumento de peso, inician Azatioprina por 4 meses. Se realizó TC-tórax detectando Timoma enviado a HG CMN La Raza para Timectomía. Ingresó a Cirugía Pediátrica, valorado por Neurología Pediátrica: prueba de hielo y agotamiento palpebral positivas, fuerza ESD 4/3/4, ESI 4/4/4, EID 4/3/4, prueba de estimulación repetitiva y anticuerpos anti-receptor de acetilcolina positivos, inició Inmunoglobulina humana (IgIV) 2 gr/kg/do, se realizó TC-tórax con lesión ovalada en mediastino de 45 × 14 × 27 mm, considerando no candidato a cirugía, trasladado a Neurología Pediátrica reiniciando Prednisona 1 mg/kg/dia, Piridostigmina 11,5 mg/kg/d. Valorado 4 meses después con franca mejoría, efectos adversos de esteroide, iniciando Micofenolato 500 mg cada 12 hrs, TC-tórax timo de 15 × 13 × 19 mm. Cursó con crisis miasténica posterior a suspensión de Prednisona en HGZ, reiniciándose ésta en forma paulatina. Valorado 2 meses después en HG CMN La Raza: con crisis miasténica, se administró IgIV 2 gr/kg/do, antiMuSK negativo. TC-tórax timo de 16 × 13 × 20 mm. Se realizó toracotomía lateral derecha, timectomía total, colocación de sonda pleural derecha, timo de 5 × 4 × 1 cm, cursó con neumotórax y atelectasia apical derecha. Reporte histopatológico Hiperplasia tímica folicular. Actualmente asintomático, Piridostigmina 7,2 mg/kg/d, Prednisona 0,3 mg/kg/d, Micofenolato 500 mg cada 12 hrs. **Conclusiones**: Los esteroides orales en ocasiones pueden inducir reducción o regresión del timo, al tener receptores en el citosol de dicho órgano, produciendo apoptosis del componente linfocítico, sin embargo, nuestro paciente requirió Timectomía.

**Palabras Clave:**

Miastenia Gravis; pediatría; prednisona; hiperplasia tímica

**Una Causa de Ataxia Que Puede Ser Reversible**

Cruz Rios Vania Kenaí^1^, Herrera Mora Patricia^2^, Lieberman Hernández Esther^3^, Leal Anaya Paula^4^

^1^Neurología Pediátrica, Instituto Nacional de Pediatría (INP), 04530 Ciudad de México, México

^2^Departamento de Neurología, Universidad Nacional Autónoma de México (UNAM), INP, 04530 Ciudad de México, México

^3^Departamento de Genética Humana, UNAM, INP, 04530 Ciudad de México, México

^4^Genética Humana, UNAM, 04530 Ciudad de México, México

**Introducción**: La ataxia con deficiencia de vitamina E (AVED) es una enfermedad genética autosómica recesiva provocada por mutaciones en el gen *TTPA*, responsable de producir la proteína encargada de transportar el alfa-tocoferol. Lo que provoca daño neuronal como consecuencia del estrés oxidativo. **Objetivo**: Se reporta un caso de un paciente adolescente con ataxia por deficiencia de vitamina E. **Resumen Clínico del Caso Con Resultado de Estudios, Tratamiento Instaurado y Evolución**: Paciente con adecuado neurodesarrollo. A los 3 años inicia con caídas frecuentes e inestabilidad en la marcha, disartria, torpeza en la coordinación motora e insensibilidad al dolor. Con progresión que le impiden bipedestación y funciones de autoayuda. A los 11 años acude al INP. La exploración neurológica revela bradilalia, marcha atáxica, dismetrías, disdiadococinesias, Romberg +, Holmes +. Se realizan los siguientes estudios: RMN cerebral 20/05/22: Asimetría ventricular supratentorial. EEG 06/09/22: Normal. Niveles de alfa tocoferol (vitamina E): <1 mg/dL (rango normal >3 mg/dL) Exoma: variante patogénica homocigota en el gen *TTPA* (c.744del). Con lo que se diagnostica AVED y se administra tratamiento suplementario. **Conclusiones**: La evolución clínica de nuestro paciente es similar a los reportado en la literatura, reportándose inicio de los síntomas durante la 1ª o 2ª década de vida. El paciente progresó a un síndrome pancerebeloso y pérdida sensorial (afectación cordones posteriores) asociado a déficit de vitamina E por variante patogénica del gen *TTPA*. La AVED, produce distrofia axonal y degeneración de la columna posterior de la médula espinal, del tracto espinocerebeloso, pérdida nuclear grácil y cuneiforme y desmielinización de los nervios periféricos. El paciente posterior a la suplementación (dependiente de la presentación farmacológica) ha detenido la progresión de la enfermedad sin lograr marcha independiente, quizá por el diagnostico tardío. No se ha determinado si es más eficaz utilizar la forma racémica fabricada químicamente o la forma natural, aunque se sabe que la proteína de transferencia de alfa-tocoferol (α-TTP) se une y transporta selectivamente los 2R-α-tocoferoles.

**Palabras Clave:**

ataxia; síndrome cerebeloso; vitamina E; gen *TTPA*; reporte de caso

**Complejo de Esclerosis Tuberosa en Pre Escolar con Mutación en TSC2**

Marco Antonio Vidal-Peregrina^1^, Fabiola Marycruz De la Fuente-Silva^1^, Martín Arturo Silva-Ramírez^1^, Flora Cebada-López^1^

^1^Instituto Mexicano del Seguro Social, UMAE Hospital General “Dr. Gaudencio González Garza” Centro Médico Nacional La Raza, 02990 Ciudad de México, México

**Introducción**: El Complejo de Esclerosis Tuberosa (CET) es la segunda facomatosis más frecuente, con herencia autosómica dominante, ocasionando alteraciones en los genes supresores de tumores, *TSC1* y *TSC2* (Tuberous Sclerosis Complex 1 y 2). Puede presentar hamartomas en prácticamente todos los tejidos. El diagnóstico definitivo se puede realizar con 2 criterios mayores o 1 criterio mayor y 2 menores, diagnóstico posible con 1 criterio mayor o 2 criterios menores, sin embargo, el identificar la mutación en el gen *TSC1* o *TSC2* es suficiente para realizar el diagnóstico. **Objetivo**: Presentación de caso de Complejo de Esclerosis Tuberosa en preescolar con mutación en gen *TSC2*. **Resumen Clínico del Caso Con Resultado de Estudios, Tratamiento Instaurado y Evolución**: Masculino de 2 años, padres con antecedente de consanguinidad. Prematuro, curso con asfixia perinatal e hiperbilirrubinemia, con retraso de neurodesarrollo. Inicia a los 6 meses con crisis atónicas cefálicas, al año de edad se agregan espasmos en extensión y flexión, se inicia Valproato de magnesio presentando somnolencia, suspendiendo éste, se inicia Vigabatrina y Levetiracetam. Valorado en HG CMN La Raza donde se detectan más de 15 manchas hipocrómicas mayores de 5 mm, manchas en confeti, se sospecha en CET. EEG con ritmo lento para la edad, paroxismos de punta onda lenta generalizada, sin integrar grafoelementos de sueño. RM-cráneo con tubérculos subcorticales y corticales, nódulos subependimarios. Se realiza panel molecular encontrando variante patogénica TSC2 en estado heterocigoto. Valorado por Cardiología encontrando rabdomiomas cardiacos. Potenciales visuales alterados, Oftalmología sin lesiones oculares. Durante seguimiento con disminución en frecuencia de crisis, continuará seguimiento multidisciplinario. **Conclusiones**: Actualmente con el diagnóstico molecular es posible detectar las mutaciones genéticas, siendo de gran utilidad para el diagnóstico temprano en casos en donde la sintomatología no es concluyente, las variantes patogénicas de TSC2 tienden a presentar un fenotipo neurológico más grave y mayor riesgo de malignidad renal que las variantes patogénicas de TSC1, siendo fundamental el asesoramiento genético a los padres. Es importante enfatizar que no todos los pacientes tendrán acceso a dicho panel, por lo que debemos recordar los criterios mayores y menores.

**Palabras Clave:**

esclerosis-tuberosa; TSC2; tubérculos-corticales; manchas-hipocrómicas

**Caracterización de Variante Patogénica en Syngap1 en Paciente Pediátrico Mexicano: Reporte de Caso**

Estrada-Gómez CG^1^, Rosas Hernández Jhonatan^2^, Espinosa-Zacarias JP^1^, Ruíz-Ferreira MS^1^

^1^Hospital Regional “Lic. Adolfo López Mateos” ISSSTE, 01030 Ciudad de México, México

^2^Universidad Autónoma de Tamaulipas, 87000 Ciudad Victoria, México

**Introducción**: El gen *SYNGAP1* (synaptic Ras GTPase activating protein) se expresa en las sinapsis excitatorias, y su reducción o pérdida genera alteraciones en la señalización neuronal, afectando la cognición y excitabilidad neuronal. Se han reportado más de 50 casos de mutaciones en este gen, generalmente *de novo*, que se caracterizan por epilepsia generalizada (84%), discapacidad intelectual (100%) y TEA, entre otros. La variante c.97del (p.Gln33Asnfs*36) se ha identificado como patogénica en la base de datos ID ClinVar 3571977, aunque no se ha asociado con un caso clínico específico. **Objetivo**: Reportar el primer caso de un paciente pediátrico mexicano con esta variante patogénica. **Resumen Clínico del Caso Con Resultado de Estudios, Tratamiento Instaurado y Evolución**: Paciente masculino con factores perinatales de riesgo que nació a las 36 SDG por taquicardia fetal, APGAR de 9. Neurodesarrollo adecuado. A los 3 años, inició con crisis epilépticas generalizadas tónico-clónicas autolimitadas asociadas a fiebre, sin tratamiento. A los 6 años, después de una nueva crisis, fue referido a nuestra unidad y se inició tratamiento con VPM, logrando control de las crisis. Persistió actividad paroxística en el EEG, por lo que se añadió TPM, con mejoría en el comportamiento. El paciente presentó conductas heteroagresivas, desafiante de la autoridad y fracaso escolar, tratado por TDAH y discapacidad intelectual leve (CI 62 en WISC-V), con mejor respuesta a atomoxetina (1 mg/kg/día). A la exploración física, normocéfalo, sin dismorfias. Funciones mentales: Orientado en persona y lugar, pero no en tiempo, memoria a corto y mediano plazo conservada, a largo plazo no, cálculo con sumas simples, no logra juicio ni abstracción, praxias y gnosias presentes. Resto sin alteraciones. EEG paroxístico en vigilia con punta onda lenta y brotes de ondas agudas en regiones frontoparietales y temporales bilaterales. RM de encéfalo sin alteraciones. **Conclusiones**: Resalta la importancia de las mutaciones en SYNGAP1 como causa de epilepsia y discapacidad intelectual. El tratamiento adecuado mejoró el control de las crisis y el comportamiento.

**Palabras Clave:**

SYNGAP1; patogénica; TEA; discapacidad intelectual; epilepsia

**Mutacion del Gen *MAP1B* Como Variante de Sentido no Descrita, un Diseño Terapeutico en Paciente con Autismo y Epilepsia**

Mercado Silva Francisco Miguel^1^, González González Eunice Maday^1^

^1^Universidad Autónoma de Guadalajara, Hospital Ángeles del Carmen, 44670 Guadalajara, México

**Introducción**: Las variantes en MAP1B herencia autosómico dominante se asocian a la heterotopia nodular periventricular tipo 9, se caracteriza por malformación del desarrollo cortical, cuerpo calloso delgado, disminución de sustancia blanca, con efectos clínicos variables. La mayoría tienen desarrollo intelectual alterado y defectos cognitivos, pueden presentar convulsiones focales. Algunos portadores menos afectados, que sugiere penetrancia incompleta del fenotipo. **Objetivo**: Reporte de caso en paciente con mutación MAP1B c.3058G>A; p.(Ala1020Thr) variante c.3058G>A; p.(Ala1020Thr) identificada en heterocigosis como variante de cambio de sentido que no ha sido previamente descrita asociada a un fenotipo especifico. Su frecuencia en bases de datos es consistente con prevalencia en enfermedades asociadas al gen. **Resumen Clínico del Caso Con Resultado de Estudios, Tratamiento Instaurado y Evolución**: Material y Metodo: Reporte de caso. Antecedentes: Masculino 10 años, padre con cardiopatía dilatada, primos con esquizofrenia, labio leporino y anoftalmia. Camino 19 meses, lenguaje 18 meses y lo perdió. Cuadro Clinico: Debuta a los 2 años con pérdida de lenguaje, estereotipias, risa y llanto sin explicación. EEG con ondas agudas fronto-temporales. Se inicio levetiracetam desapareciendo la risa y llanto. Tomografía con displasia silviana derecha y asimetría ventricular, displasia de tallo, pérdida de anatomía de mesencéfalo, IV ventrículo forma de copa invertida. Completo 2 años de tratamiento, EEG normal se suspende levetiracetam. Se instala atomoxetina y risperidona mejorando su atención, reaparece la risa y llanto sin explicación, empeora comprensión verbal, ictiosis de las palmas de las manos. Se realiza exoma mostro mutación descrita, la función mitocondrial con ácido láctico y amonio elevado, acidosis metabólica se agrega levocarnitina, coenzima Q10 y complejo B. Con mejoría actualmente. Exploración normal, hay aplasia cutis parietal e ictiosis palmar bilateral. **Conclusiones**: La determinación genómica fue crucial para diagnóstico definitivo, esta variante no aparece comúnmente en bases públicas. El cambio de Alanina por Treonina altera la interacción de la proteína MAP1B con los microtúbulos, afectando la estabilidad neuronal y el transporte intracelular. Las alteraciones en citoesqueleto afectan el transporte mitocondrial en neuronas impactando en distribución energética, dinámica mitocondrial y neurodegeneración. El cuadro clínico es atípico sin sordera ni heterotopias, pero al manejar con coenzimas para mejorar la función mitocondrial repercutió en la repuesta del paciente.

**Palabras Clave:**

MAP1B; autismo

**Características de Potenciales Evocados Visuales en el Espectro de la Neuritis Óptica en la Edad Pediátrica. Revisión de Casos**

Marquez Villaseñor Lennin Antonio^1^, Cantú Salinas Adriana Carlota^1^, De La Garza Pineda Oscar^1^, Villarreal Rodríguez Diana Laura^1^, Vázquez Fuentes Salvador^1^, Saldívar Dávila Sergio^1^, Flores Alfaro Fernanda^1^, Carrión García Ana Luisa^1^, Chávez Luevanos Beatriz Eugenia^1^

^1^Servicio de Neurología, Hospital Universitario, “Dr. José Eleuterio González”, Universidad Autónoma de Nuevo León, 64460 Monterrey, México

**Introducción**: La neuritis óptica (NO) es una lesión aguda del nervio óptico caracterizada por pérdida de agudeza visual, dolor periocular y discromatopsias. La NO en pediatría puede ser originada por un trastorno del espectro de la neuromielitis óptica, enfermedad asociada a los anticuerpos contra la mielina de los oligodendrocitos (MOGAD), infecciosas o variantes seronegativas. Los potenciales evocados visuales (PEV) establecen la ubicación anatómica de una lesión en la vía visual que no se puede determinar mediante técnicas de imagen y clasifican la afección, ya sea (desmielinizante, axonal o ambas). **Objetivo**: Describir los hallazgos de los PEV en pacientes pediátricos con NO de diferentes variantes, analizando sus patrones de disfunción y su utilidad en la clasificación de la afección. **Resumen Clínico del Caso Con Resultado de Estudios, Tratamiento Instaurado y Evolución**: Caso 1. Masculino de 13 años con diagnóstico de NO bilateral variante MOGAD. Caso 2. Femenino de 12 años con diagnóstico de NO bilateral seronegativa. Caso 3. Masculino de 7 años con diagnóstico de NO bilateral variante MOGAD. Caso 4. Femenino de 12 años con diagnóstico de NO bilateral seronegativa variante inflamatoria crónica recurrente (CRION). Caso 5. Femenino de 9 años con diagnóstico de NO bilateral variante MOGAD. Caso 6. Femenino de 12 años con diagnóstico de NO bilateral variante MOGAD. Hallazgos de PEV: Caso 1. Disfunción desmielinizante bilateral grave y disfunción axonal secundaria. Caso 2. Disfunción axonal bilateral severa y desmielinizante secundaria. Caso 3. Disfunción desmielinizante bilateral grave y disfunción axonal secundaria. Caso 4. Disfunción grave por falta de respuesta en el ojo derecho y disfunción desmielinizante leve al estimular el ojo izquierdo. Caso 5. Disfunción desmielinizante por retardo en la conducción bilateral a partir de P100. Caso 6. Disfunción severa por ausencia de respuesta al estimular el ojo izquierdo. Disfunción moderada axonal y secundariamente desmielinizante al estimular ojo derecho. **Conclusiones**: Los PEV permiten evaluar topográficamente la conducción a lo largo de la vía visual y permitir un análisis funcional-estructural detallado. Esto convierte al PEV en una herramienta altamente sensible para el diagnóstico de la NO.

**Palabras Clave:**

Neuritis óptica (NO); potenciales evocados visuales (PEV); enfermedad asociada a los anticuerpos contra la mielina de los oligodendrocitos (MOGAD); disfunción; desmielinizante; axonal

**Síntomas Neuropsiquiatricos Como Debut Clínico en Adolescente con Glioma de Alto Grado: Reporte de Caso**

Diana Laura Gutiérrez Ramírez^1^, Adriana C. Cantú Salinas^1^, José A. Sánchez Garza^1^, Ana L. Carreón García^1^, Beatriz Eugenia Chávez Luevanos^1^,

^1^Servicio de neurología, Hospital Universitario, “Dr. José Eleuterio González”, Universidad Autónoma de Nuevo León, 64460 Monterrey, México

**Introducción**: Los tumores cerebrales son el segundo tipo de cáncer más frecuente en la infancia. Los gliomas de alto grado se consideran tumores primarios malignos, altamente infiltrantes, con un pronóstico desalentador, un diagnóstico oportuno, requiere alta pericia clínica debido a la presencia de síntomas inespecíficos o también, síntomas neuropsiquiátricos. **Objetivo**: Evidenciar posibles características neuropsiquiátricas como cuadro clínico inicial ante padecimientos neurológicos graves. **Resumen Clínico del Caso Con Resultado de Estudios, Tratamiento Instaurado y Evolución**: Femenino, 13 años, mexicana. Madre finada por homicidio, padre con toxicomanías positivas y sintomatología neuropsiquiátrica. En acogimiento en familia extensa no formal, con tía paterna y abuela paterna (2022). Víctima de maltrato infantil previo a acogida. Acudía a secundaria con adecuado aprovechamiento escolar y conductas sociales. Inició en agosto del 2024 con apatía, astenia, negligencia personal, disminución de rendimiento escolar, aislamiento social e hiporexia. Se diagnóstica por psicología trastorno mixto de ansiedad y depresión. En septiembre del 2024 se agregó aparente cambio de preferencia sexual, dos semanas posteriores presentó somnolencia, alucinaciones visuales y auditivas, mutismo, incontinencia de esfínteres e hiporexia. Se inició risperidona por psiquiatría, sin mejoría. En octubre del 2024 se identifica focalidad neurológica. A la exploración neurológica presentó FOUR 14 puntos somnolencia, inatención, mutismo, midriasis bilateral 4 mm, fotomotor y consensual presentes, reflejos de estiramiento muscular +++/4, Babinski bilateral. Escala de Bush – Francis 16 puntos, catatonia moderada. RM de cerebro simple y contrastada con lesión supratentorial, intraaxial, infiltrativa, heterogénea al contraste con aparente epicentro en rodilla del cuerpo calloso, que se extendía hacia región frontal bilateral y caudal hacia núcleos grises y tálamos derechos, así como a polos temporales, condicionando efecto de masa que desplaza línea media, obliteración de espacio subaracnoideo, compresión de tercer ventrículo, edema transependimario. Se realizó craneotomía frontoparietal derecha con resección tumoral de 95%, presentando evolución tórpida. Se realizó acompañamiento con cuidados paliativos. Se diagnóstico de glioma de alto grado, H3K27 positivo. **Conclusiones**: Los síntomas neuropsiquiátricos en el contexto de tumores cerebrales requieren mayor visibilidad y alta sospecha clínica con el fin de brindar un diagnóstico y tratamiento oportuno. **Aportación al Conocimiento**: La descripción de sintomatología neuropsiquiátrica asociada a tumores cerebrales es limitada, por lo que consideramos este caso puede enriquecer el conocimiento en esta área.

**Palabras Clave:**

síntomas neuropsiquiátricos; tumor; glioma

**Hiperglicinemia no Cetósica, Perfil Clínico, Diagnóstico y Tratamiento: Reporte de Caso**

Cruz Mejía Saytin Gabriela^1^, Hernández Antúnez Blanca Gloria^1^

^1^Instituto Nacional de Pediatría, 04530 Ciudad de México, México

**Introducción**: La hiperglicinemia no cetósica (HNC) es un error innato del metabolismo que afecta el sistema mitocondrial de clivaje de glicina ocasionando su acumulación que estimula los receptores NMDA, sus principales manifestaciones clínicas son epilepsia refractaria, hipotonía, hipo, alteraciones en respiración y retraso global del neurodesarrollo. **Objetivo**: Describir el caso de un paciente con hiperglicinemia no cetósica. **Resumen Clínico del Caso Con Resultado de Estudios, Tratamiento Instaurado y Evolución**: Femenino producto de la primera gesta nace de termino sin complicaciones, egreso binomio sano, tamiz metabólico, ácidos orgánicos en orina y perfil de aminoácidos reportados normales. A los 3 días de vida presenta apneas y crisis epilépticas, requiriendo intubación orotraqueal, egresa al mes de vida con Levetiracetam y Valproato de Magnesio. A los 2 meses es referida a nuestro hospital por epilepsia refractaria, se inicia abordaje diagnóstico, descartando causas infecciosas, RMN cerebral con hipoplasia del cuerpo calloso, EEG con periodos de atenuación/supresión y actividad epileptiforme multifocal abundante, se realiza panel de epilepsia que reporta variante patogénica en el gen *AMT* asociado con encefalopatía por glicina, se realizan niveles de glicina en plasma 414 µmol/L (81–436 µmol/L) y LCR 52 µmol/L (5–26 µmol/L) cociente de glicina LCR/Plasma 0,13 µmol/L (valor normal <0,08). Se inicia manejo con benzoato de sodio y dextrometorfano. Actualmente con Vigabatrina, Levetiracetam y Topiramato, en control de crisis clínicas. **Conclusiones**: La HNC es una enfermedad rara, probablemente subdiagnosticada debido a que no genera alteraciones metabólicas fácilmente detectables y presenta alta mortalidad en periodo neonatal. La sintomatología, se caracteriza por letargo que puede evolucionar a coma, hipotonía, hipo, apneas y epilepsia de tipo mioclónica o espasmos. El EEG suele mostrar patrón de atenuación/supresión en las primeras semanas de vida y el diagnóstico se confirma con la cuantificación de glicina en plasma y LCR, con una relación LCR/plasma >0,08 µmol/L. El tratamiento con benzoato de sodio, antagonistas NMDA como dextrometorfano y dieta cetogénica han demostrado mejoría clínica en el control de crisis.

**Palabras Clave:**

hiperglicinemia no cetósica; error innato del metabolismo; epilepsia refractaria; espasmos infantiles; hipotonía

**Esclerosis Múltiple Variedad Pseudotumoral en Paciente Pediátrico. Reporte de Caso**

Galindo Fuentes Maria Isabel^1^, Ulloa Carrillo Marysol^1^

^1^Servicio de Neurología Pediátrica, UMAE Hospital de Pediatría CMNO, 44340 Guadalajara, México

**Introducción**: La esclerosis múltiple (EM) es una enfermedad crónica inflamatoria autoinmune, con desmielinización y degeneración neuroaxonal del Sistema Nervioso Central (SNC), definida como pediátrica en menores de 18 años. Existen varios subtipos clínicos: remitente-recurrente, secundariamente progresiva, primariamente progresiva y progresiva-recurrente. Sin embargo, hay formas atípicas como la EM pseudotumoral o tumefacta, una variante rara y agresiva, que representa aproximadamente 1 de cada 1000 casos de EM. Esta se manifiesta con una amplia variedad de síntomas neurológicos como alteraciones motoras, cognitivas, sensoriales, cerebelosas, de tallo cerebral, estados confusionales, defectos de los campos visuales, convulsiones y coma. En la resonancia magnética (RM) se identifican lesiones desmielinizantes mayores a 20 mm, con edema y efecto de masa perilesional simulando una neoplasia, hiperintensas en T2 con realce tras la administración de contraste. El líquido cefalorraquídeo (LCR) es positivo para bandas oligoclonales en 1/3 de los casos. El tratamiento se basa en corticoesteroides a dosis altas, plasmaféresis, inmunoglobulina y anticuerpos monoclonales. **Objetivo**: Describir un caso clínico con esclerosis múltiple pseudotumoral en edad pediátrica. **Resumen Clínico del Caso Con Resultado de Estudios, Tratamiento Instaurado y Evolución**: Adolescente de 10 años sin antecedentes de importancia, previamente sana, inicia padecimiento el 10 de Julio 2024 con pérdida de la fuerza en el brazo derecho, cefalea hemicraneal derecha, nunca dejo de caminar, pero arrastra el pie, tras consultar varios médicos sin mejoría fue referida a nuestra unidad. A su ingreso presentaba hemiparesia desproporcionada de predominio braquial y alteración de la marcha. En la RM reveló una lesión hiperintensa a nivel parietal izquierdo en FLAIR, con leve desplazamiento de la línea media, de apariencia pseudotumoral. **Conclusiones**: Se realizaron estudios complementarios, incluyendo análisis de LCR, bandas oligoclonales, anticuerpos anti-MOG y anti-aquaporina 4, que fueron negativos. Se descartaron causas infecciosas y reumatológicas. El tratamiento consistió en pulsos de corticoesteroides seguidos de Rituximab, con mejoría clínica significativa y disminución importante de las lesiones en la RM de control.

**Palabras Clave:**

esclerosis múltiple (EM); esclerosis múltiple pseudotumoral; enfermedad desmielinizante

**Uso de Dieta Cetogénica Como Tratamiento de Epilepsia Farmacorresistente en Síndrome de Rett: Reporte de un Caso**

Ponce Jáuregui Joan Manuel^1^

^1^Centro Médico Nacional 20 de Noviembre, 03104 Ciudad de México, México

**Introducción**: El síndrome de Rett (SR) es un trastorno del neurodesarrollo ligado a mutaciones en el gen *MECP2*, caracterizado por regresión neurológica, estereotipias motoras, disfunción autonómica y epilepsia. Entre el 60–90% de las pacientes desarrollan crisis epilépticas, muchas de ellas farmacorresistentes. A pesar del creciente uso de la dieta cetogénica en epilepsias intratables, existe muy poca literatura que documente su efectividad específicamente en SR, con solo cuatro reportes de casos. La dieta cetogénica clásica, al inducir cetosis sostenida, modula la neurotransmisión GABA/glutamato, reduce estrés oxidativo y regula vías como mTOR. Además del efecto anticrisis, se ha descrito mejoría en conducta, sueño e interacción social. **Objetivo**: Describir la respuesta clínica de una paciente con síndrome de Rett y epilepsia farmacorresistente tras la implementación de dieta cetogénica clásica, en el contexto de la escasa evidencia disponible. **Resumen Clínico del Caso Con Resultado de Estudios, Tratamiento Instaurado y Evolución**: Paciente femenina de 15 años con mutación patogénica en MECP2 y epilepsia multifocal desde los 9 años. Presentaba crisis tónico-clónicas generalizadas, atónicas, focales con alteración del estado de alerta a bilateral motoras. Recibió múltiples fármacos anticrisis con diferentes mecanismos: potenciadores GABAérgicos, moduladores de canales de sodio y compuestos de acción sináptica múltiple, sin lograr control satisfactorio. Se instauró dieta cetogénica clásica (relación 4:1) y se evaluó su respuesta tras cuatro meses mediante reducción de crisis epilépticas y cambio conductual con el Cuestionario de Conducta para SR. Se observó reducción del 70–80% en crisis tónico-clónicas y atónicas, y del 50% en focales motoras, mientras que las no motoras persistieron sin cambios. Hubo mejoría en sueño, alerta y comportamiento. En el seguimiento clínico, la paciente mantuvo buena respuesta, sin efectos adversos y con evolución funcional favorable. El puntaje del cuestionario conductual disminuyó de 60 a 41, con mejoría en subescalas de ansiedad/estado de ánimo, respiración anormal y conductas repetitivas. **Conclusiones**: La dieta cetogénica clásica representa una intervención eficaz y bien tolerada en epilepsia farmacorresistente asociada a SR.

**Palabras Clave:**

síndrome de Rett; epilepsia farmacorresistente; dieta cetogénica; neurodesarrollo; terapia metabólica

**Enfermedad de Moyamoya. Presentación de un Caso con Antecedentes Familiares**

Sergio Ugarte-Anchondo^1^, Antonio Bravo-Oro^1^, Jorge Luis García-Ramírez^1^, Jorge Guillermo Reyes-Vaca^1^

^1^Servicio de Neurología, Hospital Regional de Alta Especialidad “Dr. Ignacio Morones Prieto”, 78210 San Luis Potosí, México

**Introducción**: La enfermedad de Moyamoya es una vasculopatía cerebrovascular crónica y progresiva, caracterizada por estenosis u oclusión de las arterias carótidas internas y sus ramas principales, con desarrollo de una red colateral anómala con aspecto de “penacho de humo”. En edad pediátrica, suele manifestarse entre los 5 y 10 años con episodios isquémicos como hemiparesia, convulsiones o cefalea. Puede ser idiopática o asociada a síndromes genéticos. El diagnóstico se basa en la angiografía cerebral, aunque la resonancia magnética con angiografía es útil en la evaluación inicial. El tratamiento definitivo es la revascularización quirúrgica. El pronóstico depende de la detección y tratamiento oportunos. **Objetivo**: Describir la presentación clínica y hallazgos por imagen de un caso pediátrico con enfermedad de Moyamoya. **Resumen Clínico del Caso Con Resultado de Estudios, Tratamiento Instaurado y Evolución**: Paciente femenino de 5 años que presentó debilidad en el miembro torácico derecho, con extensión al miembro inferior ipsilateral, claudicación de la marcha y afasia motora no fluente. Previo al cuadro neurológico, se reportó llanto prolongado seguido de hiperventilación. Se inició abordaje por evento vascular cerebral. Como antecedente, se identificó historia familiar por la rama paterna de dos primas hermanas gemelas diagnosticadas con enfermedad de Moyamoya. **Resultados**: La resonancia magnética mostró estenosis de arterias cerebrales medias y de la arteria vertebral, así como infartos en territorios de las arterias cerebrales anterior y medias bilaterales. En la panangiografía se evidenció estenosis de la arteria basilar y tortuosidades vasculares significativas. La paciente fue hospitalizada para vigilancia y referida a neurocirugía pediátrica. **Conclusiones**: Se documenta un caso clínico compatible con enfermedad de Moyamoya, con evidencia radiológica característica y asociación familiar. Este tipo de casos resalta la importancia del reconocimiento temprano de síntomas neurológicos y del antecedente familiar, así como la necesidad de un abordaje multidisciplinario y seguimiento estrecho para reducir complicaciones neurológicas.

**Palabras Clave:**

moyamoya; angiografía cerebral; enfermedad cerebrovascular

**Sindrome de Dyke-Davidoff-Masson, Reporte de un Caso**

Méndez Fernández Daniela^1^, Saldívar Santillán Andrea^1^, Cebada López Flora^1^

^1^Instituto Mexicano del Seguro Social UMAE Hospital General CMN La Raza, “Dr. Gaudencio González Garza”, 02990 Ciudad de México, México

**Introducción**: la hemiatrofia cerebral o síndrome de Dyke-Davidoff-Masson es atribuido a trastornos intrauterinos o perinatales que afectan la perfusión de un hemisferio cerebral. Clínicamente se presenta con retraso global del desarrollo, discapacidad intelectual, epilepsia refractaria, asimetría facial, hemiplejía. Los estudios de neuroimagen con atrofia de un hemisferio cerebral y cambios compensatorios óseos ipsilaterales. **Objetivo**: Explicar espectro clínico de Síndrome de Dyke-Davidoff-Masson. **Resumen Clínico del Caso Con Resultado de Estudios, Tratamiento Instaurado y Evolución**: Se presenta el caso de un paciente masculino de 12 años con antecedente de Craneotomía a los 29 días de vida por hematoma subdural parietooccipital (29/Junio/2007), infarto hemisferio izquierdo secundario a enfermedad hemorrágica del Recién nacido. Posteriormente Inicia en mayo 2022 con crisis epiléptica refleja a estímulo auditivo, caracterizada por aumento de tono en hemicuerpo derecho con alteración en el estado de consciencia. A la exploración física destaca alteraciones en funciones mentales con alteración en memoria semántica y episódica, discalculia, alteración en abstracción, con tono aumentado en hemicuerpo derecho, trofismo disminuido en hemicuerpo derecho, fuerza 4+/4-/3 en hemicuerpo derecho, 5/5 en hemicuerpo izquierdo, reflejos de estiramiento muscular +++/++++ generalizado. Cuenta con electroencefalograma con disfunción moderada, ritmo lento de base y asimetría interhemisférica, en vigilia y sueño, paroxismos de ondas agudas en inversión de fase en región frontal derecha durante el sueño. Su resonancia magnética de cráneo 9/09/2022 con hemiatrofia cerebral izquierda con múltiples áreas de malasia, gliosis asociadas a engrosamiento compensatorio del diploe, y atrofia walleriana izquierda, datos compatibles con Dyke-Davidoff-Masson. **Conclusiones**: El Síndrome Dyke-Davidoff-Masson es una entidad neurológica poco común con hemiatrofia cerebral, ocasionada por trastornos intrauterinos o perinatales de origen vascular, Dentro de esta patología lo más grave es su presentación con epilepsia farmacorresistente y estado epiléptico refractario, por lo cual es importante un reconocimiento de factores de riesgo, así como un abordaje y tratamiento oportuno.

**Palabras Clave:**

epilepsia; Dyke-Davidoff-Masson; farmacorresistencia

**Variante Patogénica del Gen *KCNMA1* Como Causa de Epilepsia Farmacorresistente. Reporte de Caso**

López Gallegos-Gabriela Monserrat^1^, Castro Ruiz-Alfredo^1^, Reyes Cuayahuitl-Araceli^1^, Rayo Mares-Jesús Darío^1^

^1^Hospital de Pediatría CMN Siglo XXI, IMSS, 06720 Ciudad de México, México

**Introducción**: El gen *KCNMA1* codifica la subunidad alfa del canal BK activado por calcio. Esta proteína participa en diversos procesos fisiológicos, como la contracción del músculo liso, la liberación de neurotransmisores y la excitabilidad neuronal. Las mutaciones en este gen se asocian con diversos trastornos neurológicos, como la discinesia paroxística no cinesiogénica, la epilepsia generalizada idiopática y el síndrome de Liang-Wang. Puede presentar un fenotipo variable, con retraso global del desarrollo, retraso o ausencia del lenguaje, hipotonía axial, dismorfias craneofaciales y asociación con crisis epilépticas generalizadas. **Objetivo**: Descripción clínica y paraclínica de un caso con epilepsia y variante patogénica del gen KCNMA1. **Resumen Clínico del Caso Con Resultado de Estudios, Tratamiento Instaurado y Evolución**: Masculino de 2 años, con retraso de los hitos del desarrollo desde, sin antecedentes de importancia. Inicia a los 11 meses con espasmos epilépticos; al año de edad crisis atónicas, se inicia manejo con valproato de magnesio. A los 15 meses se agregan crisis tónicas generalizadas (10/semana). A los 2 años crisis de ausencia atípicas (2–3/día). Previamente tratado con lamotrigina, topiramato, clonazepam, valproato semisódico y CDB con persistencia de crisis epilépticas. Cuenta con registro electroencefalográfico con frecuentes paroxismos de puntas y complejos punta-onda lenta generalizada de predominio centrotemporal izquierda. Resonancia magnética con atrofia cortical frontal bilateral e hipoplasia del cuerpo calloso. Se realiza estudio molecular encontrando variante patogénica del gen KCNMA1. **Conclusiones**: Las canalopatías *KCNMA1* son combinaciones raras de síntomas neurológicos que incluyen crisis epilépticas, trastornos del movimiento, retraso en el desarrollo y discapacidad intelectual. Se asocia asimismo con el síndrome de Liang-Wang (retraso del desarrollo, deterioro del desarrollo intelectual y habla deficiente, dismorfia craneofacial y anomalías viscerales y del tejido conectivo) aproximadamente el 50% de los pacientes presentan anomalías en las imágenes cerebrales, en particular atrofia cerebral y cerebelosa e hipoplasia del cuerpo calloso.

**Palabras Clave:**

canal; epilepsia; crisis epilépticas; farmacorresistente

**Distrofia Muscular de Duchenne: Variantes Genéticas, Incluyendo C.6283C>T (P.ARG2095TER) y su Asociación con la Progresión Rápida de la Debilidad Muscular**

Arias Méndez Jesús Antonio^1^, Valladares Sánchez Pablo^1^, Cornelio Nieto Jose Ovidio^1^

^1^Unidad Médica de Neurología, Hospital Regional de Alta Especialidad del Niño Dr. Rodolfo Nieto Padrón, 86030 Villahermosa, México

**Introducción**: La Distrofia Muscular de Duchenne (DMD) es una enfermedad genética recesiva ligada al cromosoma X que afecta a los músculos esqueléticos, causando una degeneración muscular progresiva. Su prevalencia se encuentra en varones, manifestándose generalmente en la infancia. La detección temprana y el tratamiento adecuado pueden ralentizar la progresión de la enfermedad, pero no existe cura definitiva. **Objetivo**: El objetivo de este estudio es presentar y analizar cinco casos clínicos de pacientes con diagnóstico de Distrofia Muscular de Duchenne, describiendo sus características clínicas, tratamiento y evolución. **Resumen Clínico del Caso Con Resultado de Estudios, Tratamiento Instaurado y Evolución**: Se presentan cinco casos clínicos de pacientes diagnosticados con DMD, con edades entre 7 y 12 años (promedio 9,4 años). El diagnóstico se realizó a una edad promedio de 6,4 años, tras la aparición de síntomas alrededor de los 4,2 años. Se recopiló información sobre los síntomas iniciales, tratamiento recibido, progresión de la enfermedad y resultados de los estudios genéticos. **Conclusiones**: La Distrofia Muscular de Duchenne se presenta con síntomas clínicos típicos y una evolución progresiva de la debilidad muscular. El tratamiento con prednisona y fisioterapia ayuda a ralentizar la progresión, pero la enfermedad sigue siendo debilitante. La identificación temprana y el manejo adecuado de las complicaciones, como la atrofia muscular y la hipotonía, son fundamentales para mejorar la calidad de vida de los pacientes.

**Palabras Clave:**

Distrofia Muscular de Duchenne; enfermedad genética; degeneración muscular; diagnóstico; tratamiento; prednisona; fisioterapia; complicaciones neurológicas

**Empiema Subdural en la Infancia: Una Complicación Silenciosa de la Sinusitis**

Jacqueline D. Lucio-Escamilla^1^, Cantú Salinas Adriana Carlota^1^, Carrión García Ana Luisa^1^, Vázquez Fuentes Salvador^1^, De la Garza Pineda Oscar^1^, Chávez Luévanos Beatriz Eugenia^1^

^1^Servicio de Neurología, Hospital Universitario “Dr. José Eleuterio González”, Universidad Autónoma de Nuevo León, 64460 Monterrey, México

**Introducción**: El empiema subdural es una acumulación purulenta entre la duramadre y la aracnoides, constituyendo una emergencia neurológica en pediatría. Puede presentarse como complicación intracraneal de infecciones sinusales, especialmente frontales o etmoidales no tratadas o tratadas de forma parcial, con una incidencia de hasta el 3,7% entre estas complicaciones. La clínica puede incluir fiebre, cefalea, vómitos, signos meníngeos, alteraciones del estado de conciencia, focalización neurológica y convulsiones, con una presentación habitualmente insidiosa o subaguda. La resonancia magnética con contraste es el estudio de elección para su diagnóstico, y el tratamiento requiere antibioticoterapia empírica de amplio espectro. A pesar de los avances terapéuticos, la morbilidad y mortalidad neurológica siguen siendo elevadas, por lo que la detección precoz es clave para mejorar el pronóstico. **Objetivo**: Describir un caso de empiema subdural secundario a sinusitis, destacando la importancia del diagnóstico temprano ante signos neurológicos sutiles. **Resumen Clínico del Caso Con Resultado de Estudios, Tratamiento Instaurado y Evolución**: Femenina de 13 años de edad, con antecedentes de episodios recurrentes de sinusitis parcialmente tratados, que inició con fiebre y cefalea de característica punzante de localización en región frontotemporal bilateral, con alivio parcial tras la administración de analgésicos. Posteriormente, presentó astenia, adinamia, así como parestesias y debilidad progresiva en el miembro inferior izquierdo, con limitación para la marcha, que progresó hacia miembro superior ipsilateral. Neurológicamente se encontró hemiparesia izquierda, con fuerza muscular 2/5 e hiporreflexia ipsilateral. Sin signos meníngeos, con LCR sin alteraciones significativas. La resonancia magnética de encéfalo con contraste reportó un empiema subdural frontoparietal derecho, con extensión hacia la región interhemisférica, acompañado de hallazgos compatibles con paquimeningitis y leptomeningitis. Asimismo, se identificó pansinusitis derecha con signos de agudización. Se inició tratamiento antimicrobiano empírico con esquema intravenoso a base de ceftriaxona, metronidazol y vancomicina, con evolución favorable y remisión progresiva del déficit neurológico focal. **Conclusiones**: Este caso resalta la necesidad de mantener un alto índice de sospecha clínica ante cualquier deterioro neurológico en pacientes con sinusitis, reafirmando la importancia de no subestimar esta patología común en la práctica pediátrica.

**Palabras Clave:**

empiema subdural; sinusitis; complicaciones intracraneales; diagnóstico precoz

**Epilepsia Farmacorresistente: Displasia Cortical Focal Asociada a Sindrome de Lennox Gastaut**

María de los Ángeles Olivares-Gutiérrez^1^, Ana Luisa Carrión-Garcia^1^, Adriana Carlota Cantú-Salinas^1^, José Ascención Arenas-Ruiz^1^, José Rogelio Bonilla-Galván^1^, Luis Enrique Flores-Huerta^1^, Ángel Raymundo Martínez-Ponce de León^1^, Armando Diaz-Martínez^1^, Salvador Vásquez-Fuentes ^1^, Beatriz Eugenia Chávez-Luévanos^1^

^1^Servicio de Neurología, Servicio de Neurocirugía, Hospital Universitario “Dr. José Eleuterio González”, 64460 Monterrey, México

**Introducción**: Displasia cortical un trastorno de la migración neuronal, manifestación más frecuente es epilepsia farmacorresistente (EFR); de los cuales aproximadamente 46,5% presentan alguna forma de esta patología. La epilepsia es frecuente en pediatría, incidencia aproximada 45/100.000 niños y prevalencia 0,5 a 0,8/1000 habitantes anualmente; siendo farmacorresistentes 25–30%, de ellos 5–10% pueden ser candidatos a cirugía. Las crisis producen daño encefálico irrecuperable por lo que se plantea terapéutica quirúrgica tempranamente en casos de EFR aunque las crisis lleven pocos meses de evolución. **Objetivo**: Analizar la cirugía de epilepsia como opción terapéutica en pacientes con displasia cortical como causa de epilepsia farmacorresistente. **Resumen Clínico del Caso Con Resultado de Estudios, Tratamiento Instaurado y Evolución**: Femenina 5 años de edad, sin antecedentes familiares, control prenatal adecuado. Nacida a término, cesárea por cesárea previa, antropometría adecuada, egresó con madre sin complicaciones. A los 15 dias de vida inician espasmos 3–4 episodios diarios, los cuales fueron aumentando. A los 30 días de vida presenta 40–50 espasmos diarios, iniciándose fármaco anticrisis, a los 45 días con crisis tónicas focales; posteriormente tónicas focales con bilateralización. A los 4 meses inició con crisis tónico-clónica generalizada. Desde entonces, presenta crisis diarias, con duración de 30 segundos hasta 2 minutos, uso de múltiples fármacos anticrisis y múltiples hospitalizaciones. Paciente presenta retraso global del desarrollo., balbuceaba y logró sedestación, rara vez bipedestación asistida; diagnosticada con Síndrome de Lennox Gastaut, siendo evaluada y transferida a cirugía de epilepsia donde Electroencefalograma (EEG): complejos punta-onda lenta y actividad paroxística rápida, Resonancia cerebral: aumento de señal en sustancia blanca periventricular, periatrial izquierda. Se realizó cirugía: Lesionectomía frontal derecha asistida por electrocorticografía, enviándose biopsia: Displasia cortical focal IIB. Posterior a cirugía crisis de 1–6 episodios diarios, tónico-clónica generalizada, 20–30 segundos de duración. EEG sin actividad intertical, ni actividad epiléptica. Actualmente paciente forma oraciones de 2 palabras y logra deambulación independiente. **Conclusiones**: La cirugía es un procedimiento quirúrgico eficaz y seguro para paciente EFR que presentan riesgo de retraso del desarrollo y muerte prematura, reduciendo de significativamente frecuencia, número y duración de crisis a corto y largo plazo precozmente previniendo mayores daños cognitivos.

**Palabras Clave:**

displasia cortical; síndrome de Lennox Gastaut; epilepsia; epilepsia farmacorresistente; cirugía

**Síndrome de Megalencefalia-Malformación Capilar-Polimicrogiria: Reporte de Caso**

Cruz Rios Vania Kenaí^1^, Rios Flores Braulio^2^, Olivas Peña Efraín^2^, Yáñez Félix Ana Lucía^2^, López Rodríguez Larissa^2^, Sevilla Montoya Rosalba^2^

^1^Instituto Nacional de Pediatría (INP), 04530 Ciudad de México, México

^2^Instituto Nacional de Perinatología (INPer), 11000 Ciudad de México, México

**Introducción**: El síndrome de megalencefalia-malformación capilar-polimicrogiria (MCAP) es una afección neurológica rara (hasta el 2022 reportándose 300 casos a nivel mundial5) caracterizada por macrocefalia, anomalías vasculares en piel y sobrecrecimiento corporal focal o segmentario. **Objetivo**: Reportamos un caso de paciente con diagnóstico de MCAP, realizando seguimiento pre y post natal. **Resumen Clínico del Caso Con Resultado de Estudios, Tratamiento Instaurado y Evolución**: Recién nacido de término de 39,2 SDG con seguimiento en INPer a partir de las 34,5 SDG por ventriculomegalia fetal. Se realiza resonancia magnética (RM) prenatal confirmando ventriculomegalia y sangrado de la matriz germinal in útero. Peso al nacer de 4835 gr y talla de 56 cm. Presenta dismorfias menores, así como malformación vascular profunda en tronco y extremidades. Se realiza RM cerebral neonatal encontrando ventriculomegalia, polimicrogiria en región opercular bilateral, disgenesia de cuerpo calloso y depósito de hemosiderina en surco caudado talámico. En base a criterios clínicos se establece diagnóstico de MCAP. Presenta EEG con amplitud integrada (9 canales) anormal con nueve crisis electrográficas (región temporal derecha) durante 24 horas, por lo cual se inicia tratamiento con levetiracetam. **Conclusiones**: El síndrome de MCAP, es un síndrome genética asociado a variantes en PIK3CA y otros genes de la vía PI3K-AKT, caracterizada por megalencefalia, malformaciones capilares cutáneas, sobrecrecimiento segmentario y/o asimetría corporal, dedos anchos y/o sindactilia parcial, alteraciones cerebrales estructurales, como polimicrogiria y retraso en el desarrollo y/o discapacidad intelectual. Las imágenes cerebrales pueden mostrar polimicrogiria, asimetría ventricular, hidrocefalia, engrosamiento del cuerpo calloso, herniación cerebelosa y menos descrito, heterotopias. El tratamiento es sintomático e incluye manejo de crisis epilépticas, terapia de rehabilitación y, en algunos casos, cirugía para hidrocefalia o compresión cerebelosa. El diagnóstico de MCAP es un desafío por su variabilidad fenotípica, sin embargo, existen criterios clínicos y de neuroimagen establecidos que permiten brindar el diagnóstico clínico, como en el caso del paciente. Hacer una confirmación genética permitiría establecer relación genotipo-fenotipo y aportaría a conocer mejor la condición.

**Palabras Clave:**

megalencefalia; polimicrogiria; síndrome de hipercrecimiento; reporte de caso

**Guillain-Barré Pediátrico: Expandiendo el Espectro Clínico**

Jacqueline D. Lucio-Escamilla^1^, Cantú Salinas Adriana Carlota^1^, Carrión García Ana Luisa^1^, Vázquez Fuentes Salvador^1^, De la Garza Pineda Oscar^1^, Chávez Luévanos Beatriz Eugenia^1^

^1^Servicio de Neurología, Hospital Universitario “Dr. José Eleuterio González”, Universidad Autónoma de Nuevo León, 64460 Monterrey, México

**Introducción**: La polirradiculoneuropatía aguda de origen autoinmune o síndrome de Guillain-Barré (SGB) actualmente es la principal causa de parálisis flácida aguda en la población pediátrica. Con una incidencia menor que en adultos, de 0,4 a 1,3 casos por cada 100.000 niños menores de 14 años en Mexico; siendo la variante más común del SGB en América Latina la polirradiculoneuropatía desmielinizante inflamatoria aguda (AIDP), caracterizada por debilidad muscular de inicio en miembros inferiores ascendente, progresiva, con arreflexia y disociación albúmino-citológica en el líquido cefalorraquídeo. De forma menos frecuente se presentan cuadros clínicos atípicos como debilidad de inicio en miembros superiores, debilidad faringo cervico braquial, inicio asimétrico, paraparesia, parálisis del VI nervio craneal o la presencia de signos piramidales. **Objetivo**: Describir dos casos de Guillain-Barré pediátrico con manifestaciones atípicas, ampliando el espectro clínico de esta patología. **Resumen Clínico del Caso Con Resultado de Estudios, Tratamiento Instaurado y Evolución**: Paciente 1: Masculino de 15 años con antecedente de infección gastrointestinal previa. Inició con parestesias, debilidad e hiporreflexia en miembro superior izquierdo, con progresión al miembro superior derecho y posteriormente al resto de las extremidades; presentando en el LCR disociación albumino-citológica y en los VCNMS reportando polirradiculoneuropatía desmielinizante. Paciente 2: Femenina de 7 años con antecedente de infección gastrointestinal previa. Inició con dolor 4/10 en la escala análoga del dolor, debilidad en miembro pélvico derecho que se propaga contralateralmente, con progresión ascendente acompañado de hiporreflexia y respuesta plantar extensora bilateral. En LCR con disociación albúmino-citológica y en los ECN con polirradiculoneuropatía desmielinizante. Ambos pacientes se les manejó con inmunoglobulina a dosis de 1 g/kg/día, egresándose con un puntaje de 2 de la Guillain Barre Disability Score (GDS). **Conclusiones**: Las manifestaciones atípicas observadas como el inicio en miembros superiores, debilidad asimétrica y presencia de signos piramidales, amplían el espectro clínico del Guillain-Barré pediátrico, subrayando la imperiosa necesidad de un diagnóstico clínico oportuno e individualizado para optimizar la terapéutica, mejorando el pronóstico funcional de los pacientes.

**Palabras Clave:**

síndrome de Guillain-Barré pediátrico; manifestaciones atípicas; diagnóstico clínico; disociación albúmino-citológica; velocidades de conducción nerviosa

**Espectro Fenotípico de la Variante SCN1A c.1143G>c (P. GLN381HIS) Reporte de un Caso**

López García Ameyalli^1^, Ruiz Ferreira María Soledad^1^, Espinoza Zacarias Juan Pedro^1^

^1^Hospital Regional “Lic. Adolfo López Mateos”, 01030 Ciudad de México, México

**Introducción**: El gen *SCN1A*, codifica la subunidad alfa del canal iónico de sodio dependiente de voltaje, que se encuentra asociado a varios fenotipos de epilepsia incluidos el síndrome de Dravet, la epilepsia genética con crisis febriles plus y encefalopatías epilépticas infantiles. El tipo y la ubicación de las variantes hacen diferencias en la gravedad del trastorno. **Objetivo**: Analizar la heterogeneidad clínica de SCN1A confirmado por prueba molecular y revisión de la literatura. **Resumen Clínico del Caso Con Resultado de Estudios, Tratamiento Instaurado y Evolución**: Masculino de 4 años, sin antecedentes de importancia neurológica o riesgo perinatal. Inicia al año con crisis focales con supra versión de la mirada y desviación al lado izquierdo a bilateral tónico clónico, asociado a fiebre. A los 3 años, sin asociarse a fiebre, presenta supraversión de la mirada, posición tónica de las cuatro extremidades y movimientos clónicos. Periodo post ictal caracterizado por somnolencia. A la exploración neurológica, perímetro cefálico 51 cm (P50), normocéfalo, sin facies sindrómica, pares craneales sin alteraciones, sistema motor Tono y trofismo conservado. Fuerza muscular 5/5 en escala de Daniels. REM’s ++ global. Respuesta plantar flexora. Sin clonus. Sensibilidad y cerebelo sin alteraciones. Marcha independiente. Logra punta, talón, tándem. Romberg: Negativo. Sin presencia de estigmas neurocutáneos. Denver: Acorde a su edad. OCT sin alteraciones. Audiometría: Sin alteraciones. Tratamiento anticrisis: Valproato de Magnesio. EEG: En vigilia se observa complejos de ondas agudas. Panel genético para epilepsia: SCN1A c.1143G>c. **Conclusiones**: La variante genética mencionada ha sido confirmada en un paciente con síndrome de Dravet, sin embargo, en este caso el paciente presenta una correlación genotipo-fenotipo que no cumple criterios de acuerdo con la ILAE para síndrome de Dravet. Las pruebas genéticas acortan el tiempo de diagnóstico individual y pueden ser beneficiosas para seleccionar el tratamiento anticrisis y mejorar la calidad de vida a largo plazo de los pacientes.

**Palabras Clave:**

SCN1A; síndrome de Dravet; heterogeneidad

**Quiste Dermoide Abscedado Como Causa de Compresión Medular en el Paciente Pediátrico**

Guzmán García Diana^1^, Díaz de León Cano Eduardo^1^, Cantú Salinas Adriana Carlota^1^

^1^Hospital ISSSTE Regional, 64380 Monterrey, México

**Introducción**: El quiste dermoide es una malformación congénita benigna poco frecuente, presente en el 0,3% de los recién nacidos. Se origina por el secuestro de tejido ectodérmico durante el cierre del tubo neural y presenta un crecimiento lento. Histológicamente contiene epitelio escamoso, folículos pilosos y glándulas sebáceas. Aunque suele ser asintomático inicialmente, puede ocasionar síntomas neurológicos debido a efecto de masa, además, pueden presentar complicaciones como meningitis recurrentes con riesgo de abscesos. Se asocia a otras anomalías congénitas en la mitad de los casos. **Objetivo**: El propósito de este estudio es proporcionar información sobre este padecimiento, enfocándonos en factores de riesgo y los datos clínicos tempranos para identificar signos de alarma neurológicos de forma oportuna y prevenir secuelas graves. **Resumen Clínico del Caso Con Resultado de Estudios, Tratamiento Instaurado y Evolución**: Paciente pediátrico de 15 meses con antecedente de médula anclada detectada a los 4 días de vida debido a la presencia de una fosa lumbar, se refiere cirugía a los 8 meses. Inició padecimiento actual 16 días previos al ingreso al presentar fiebre, irritabilidad y pérdida de fuerza y sensibilidad en extremidades inferiores que le impedía la bipedestación. Consultó con múltiples médicos sin recibir un diagnóstico claro y al no tener respuesta a tratamiento 4 días previos al ingreso acudió a una unidad de urgencias donde al presentar signos meníngeos y una punción lumbar patológica donde se aisló Streptococcus anginosus, se refirió a nuestra institución. Al ingreso, presentaba hipotonía severa, fuerza muscular 0/5 en miembros inferiores y ausencia de reflejos osteotendinosos. Se realizó una resonancia magnética contrastada que evidenció presencia de dos abscesos en canal medular lumbar. Requirió antibioticoterapia intravenosa (Meropenem 40 mg/kg/do) además de dos intervenciones neuroquirúrgicas. Posterior a esto, el paciente mostró mejoría progresiva, con resolución de lesiones por imagen y recuperando funcionalidad de extremidades inferiores. **Conclusiones**: Este caso resalta la importancia de reconocer signos de alarma neurológicos en pediatría, ya que su omisión puede retrasar el diagnóstico y tratamiento, aumentando el riesgo de complicaciones graves y elevando la morbimortalidad.

**Palabras Clave:**

absceso intramedular; quiste dermoide; Streptococcus anginosus

**Síndrome de Rett Masculino: Un Periplo Neurológico**

Guerrero De León Jorge Eduardo^1^, Ancer Ochoa Jorge A^1^, Calderón Sepúlveda Raúl Fernando^1^

^1^Hospital Regional Materno Infantil de Nuevo León, 67100 Guadalupe, México

**Introducción**: El síndrome de Rett (RTT) es un trastorno del neurodesarrollo que se presenta casi exclusivamente en mujeres. La mayoría de los casos están causados por variantes patogénicas del gen *MECP2*, localizado en Xq28 y que codifica la proteína 2 de unión a metil-CpG (MeCP2). Su deficiencia provoca un fallo en la maduración y el mantenimiento sináptico de la corteza, condicionando arresto del desarrollo entre los 6–18 meses, regresión de las habilidades adquiridas, pérdida del habla, movimientos estereotipados, microcefalia, crisis convulsivas y discapacidad intelectual. El espectro en los varones va desde la encefalopatía neonatal grave con signos piramidales, parkinsonismo y síndrome de macroorquidismo hasta la discapacidad intelectual grave. Afecta 1/10.000 mujeres nacidas vivas y se ha descrito ocasionalmente en varones, con un curso letal en la primera infancia. **Objetivo**: Describir el curso clínico de un masculino con RTT y evolución prolongada con complicaciones respiratorias, disautonomía y resistencia GABAérgica, destacando el abordaje multidisciplinario. **Resumen Clínico del Caso Con Resultado de Estudios, Tratamiento Instaurado y Evolución**: Masculino de 1 año 5 meses con diagnóstico genético de RTT (variante homocigótica patogénica en MECP2). Ingresó por fiebre, cianosis y deterioro neurológico. Requirió ventilación mecánica invasiva y presentó resistencia a Midazolam a dosis altas (1000 mcg/kg/h), sugiriendo disfunción del sistema GABAérgico. Episodios de bradicardia e hipertensión compatibles con disautonomía. TAC sin hallazgos relevantes. Síndrome convulsivo tónico generalizado, con EEG con intermitencia de puntas y complejos punta-onda aguda centrotemporooccipitales. Recibió sedación ajustada, antibióticos por neumonía asociada a VMI, Levetiracetam y melatonina. A la fecha, continúa hospitalizado con dependencia ventilatoria, sin nuevas crisis, y evolución neurológica estacionaria. **Conclusiones**: El RTT en varones representa una entidad de alta complejidad clínica y mal pronóstico. Este caso destaca la resistencia a benzodiacepinas, relacionada con alteración GABAérgica, así como la disautonomía severa que puede simular otros cuadros críticos. Se enfatiza la necesidad de un manejo individualizado y prolongado, especialmente en contextos con recursos limitados.

**Palabras Clave:**

síndrome de Rett; sexo masculino; gen *MECP2*; epilepsia

**Sindrome Hemicerebeloso Secundario a Diseccion Espontanea de la Arteria Vertebral**

Burgos Navarro Alan^1^, Huerta Hurtado Alma Maritza^1^, Mendoza Domínguez Irving Rubén^1^

^1^Unidad Médica de Alta Especialidad, Hospital de Pediatría, Centro Médico Nacional de Occidente (CMNO), 44340 Guadalajara, México

**Introducción**: La disección de la arteria vertebral (DAV) es una causa frecuente de accidente cerebrovascular (ACV) en pacientes <45 años, corresponde 2% de los mismos y su incidencia en entre 1,5–2 casos por cada 100.000. En el ámbito pediátrico la incidencia es baja. Se produce por la entrada de sangre a la media a través de un desgarro en la íntima. Es potencialmente mortal y puede ser difícil de diagnosticar clínica y radiológicamente. Dentro de las causas tenemos traumatismo directo severo o trauma menor y de baja energía (alguna manipulación del cuello o hiperextensión del mismo). La displasia fibromuscular y/o enfermedades del tejido conectivo son otras causas. Las disecciones a nivel de segmento 3–4 son los más afectados. **Objetivo**: Describir un caso clínico con presencia de síndrome hemicerebeloso derecho secundario a ACV en territorio de la arteria cerebelosa posteroinferior (PICA) secundario a DAV derecha. **Resumen Clínico del Caso Con Resultado de Estudios, Tratamiento Instaurado y Evolución**: Descriptivo, Paciente masculino de 14 años de edad previamente sano, quien acude referido de Morelia a la UMAE Hospital de Pediatría CMNO por presencia lenguaje escandido, reflejos de estiramiento muscular pendulares, disdiadococinecias, dismetrías a expensas de hipermetrías y ataxia con lateralización a la derecha además de desviación de la úvula a la izquierda y reflejo nauseoso abolido (IX par). Se realiza TAC de cráneo con presencia de hipodensidad en hemisferio cerebeloso derecho y medula oblonga del mismo lado. Se toma angio IRM evidenciando la DAV. Se realiza valoración multidisciplinaria y se da tratamiento con enoxaparina por un 1 mes después ribaroxaban. Se realiza angiografía en donde se observa zona de disección y oclusión de la arteria vertebral derecha en segmento V4, no se requirió la angioplastía. **Conclusiones**: La DAV es rara en la población pediátrica. En este caso se descartaron todas las posibles causas y se concluyó que fue una disección traumática de baja potencia por un probable movimiento brusco de cuello. A 6 meses del evento presenta recuperación clínica completa. Se mantiene en vigilancia puesto que el 10% puede tener recurrencia de dichos ataques.

**Palabras Clave:**

accidente cerebrovascular; disección; arteria vertebral; traumatismo

**Ataxia Telangiectasia. Reporte de Caso en Paciente PediÁTrico**

Galindo Fuentes María Isabel^1^, Armejo Chávez Luz Elena^1^

^1^Servicio de Neurología Pediátrica, UMAE Hospital de Pediatría CMNO, 44340 Guadalajara, México

**Introducción**: La ataxia telangiectasia (AT) es un trastorno neurodegenerativo autosómico recesivo que comienza en la infancia se caracteriza por ataxia cerebelosa progresiva, coreoatetosis, telangiectasias oculocutáneas, inmunodeficiencia y sensibilidad celular a la radiación ionizante. La prevalencia varía entre 1:40.000 a 1:300.000. Esta enfermedad es causada por mutaciones en el gen *ATM* (11q22-q23) que conduce a una expresión anormal de la proteína ATM, la cual es una quinasa serina/treonina crucial para el reconocimiento y la respuesta celular al daño de ADN por radiación ionizante en las roturas de doble hebra en el ADN. Clínicamente se manifiesta con ataxia cerebelosa progresiva, apraxia oculomotora, trastornos del movimiento (coreoatetosis, distonía, mioclonía, temblor de acción), telangiectasias oculocutáneas y disfunción cognitiva, además presenta afectación multisistémica incluyendo inmunodeficiencia, endocrinopatía y radiosensibilidad; y se asocia con un alto riesgo de cáncer, principalmente leucemia y linfoma. La resonancia magnética (RM) demuestra hipoplasia cerebelosa de vermis y de ambos hemisferios cerebelosos. El diagnóstico definitivo se establece mediante pruebas genéticas. **Objetivo**: Describir un caso clínico de un paciente pediátrico con ataxia telangiectasia. **Resumen Clínico del Caso Con Resultado de Estudios, Tratamiento Instaurado y Evolución**: Descriptivo, reporte de caso. Adolescente de 11 años, con antecedente familiar de hermana de 8 años diagnosticada con ataxia-telangiectasia (mutación patogénica heterocigota en el gen *ATM* C.2921+1g>A). Inicia padecimiento a los 9 años al presentar caídas frecuentes, marcha atáxica, temblor y posterior de un año ayuda para la deambulación. A la exploración física presenta telangiectasias en conjuntiva bulbar bilaterales, temblor que se exacerba con el movimiento, marcha con ayuda con incremento en plano de sustentación y ataxia marcada. Se inicio protocolo de estudio con reporte de Alfafetoproteína elevada (309 ng/mL) y linfopenia a expensas de linfocitos B y T. La RM evidenció hipoplasia cerebelosa y del vermis. Sin presencia de otra alteración sistémica. Pendiente toma de panel genético para confirmar diagnóstico. **Conclusiones**: La AT es una enfermedad rara con afectación multisistémica por lo que el diagnóstico temprano permite implementar un manejo multidisciplinario enfocado en la prevención de complicaciones y vigilancia de riesgo de cáncer.

**Palabras Clave:**

ataxia telangiectasia; ataxia; síndrome neurocutáneo

**Estimulación Cerebral Profunda en Paciente Pediátrico con Distonía Generalizada DYT 1A**

Rivera Figueroa Lily^1,2^, Cobilt Catana Rafael^1^, Sandoval Olivares Lizbeth Itzel^2,3^

^1^Hospital Star Médica Lomas Verdes, 53250 Naucalpan de Juárez, México

^2^Centro Médico Nacional “20 de Noviembre”, 03104 Ciudad de México, México

^3^Hospital Ángeles Acoxpa, 14308 Ciudad de México, México

**Introducción**: La distonía es un trastorno del movimiento caracterizado por una contracción muscular sostenida o intermitente, que causa movimientos o posturas anormales y/o repetitivas. No se conoce exactamente su la incidencia y prevalencia debido a la variabilidad metodológica utilizada en las publicaciones, oscila entre 3–4 casos por cada 100.000 habitantes. Se ha producido un cambio de paradigma en el diagnóstico genético molecular, con las técnicas de secuenciación de nueva generación (NGS) que ahora permiten analizar cientos de genes simultáneamente. **Objetivo**: Describir el abordaje diagnóstico, tratamiento con estimulación cerebral profunda y evolución post operatoria de un paciente pediátrico con diagnóstico de distonía generalizada DYT 1A. **Resumen Clínico del Caso Con Resultado de Estudios, Tratamiento Instaurado y Evolución**: Presentamos el caso de masculino de 13 años, referido para descartar parálisis cerebral infantil; no cuenta con antecedentes familiares positivos, sin antecedentes perinatales de riesgo, neurodesarrollo reportado dentro de parámetros normales. Inició su padecimiento a los 7 años con alteración progresiva en la marcha, con inversión de pie izquierdo, posteriormente afectación bilateral, evolucionó de deterioro progresivo de la marcha, a los 9 años se identifica afección de miembros superiores con predominio izquierdo, tratado con múltiples estrategias de fisioterapia sin mejoría. En exploración inicial se observa alteración del movimiento generalizada con contracciones musculares intermitentes que condicionaban alteración postural en extremidades y tronco, con predominio en extremidades izquierdas, empeoramiento con la realización de movimientos y desbordamiento, se indica el estudio genético que se reporta con mutación patogénica del gen *TOR 1A*, corroborándose diagnóstico de distonía generalizada DYT 1A, padecimiento progresivo y discapacitante, es propuesto para estimulación cerebral profunda en globo pálido interno bilateral, cirugía llevada a cabo sin complicaciones, presentó mejoría en el postoperatorio inmediato. Parámetros de estimulación Ancho de pulso: 80 s, amplitud 1,5 mA, frecuencia 89 Hz, trihexifenidilo (10 mg/día) a seis meses de cirugía con mejoría en la deambulación e integrado a actividades cotidianas. **Conclusiones**: Reconocer tempranamente las distonías generalizadas que inician predominantemente en edad pediátrica permite ofrecer tratamiento quirúrgico, previniendo complicaciones como contracturas y estigma social; la estimulación cerebral profunda del globo pálido interno es un procedimiento con morbilidad mínima.

**Palabras Clave:**

distonía; estimulación cerebral profunda; DYT 1A

**Síndrome de Rett en un Hospital de Tercer Nivel: Reporte de Caso**

Sánchez López Sergio Lisandro^1^, Armejo Chávez Luz Elena^1^

^1^UMAE Hospital de Pediatría CMNO, 44340 Guadalajara, México

**Introducción**: El síndrome de Rett es un trastorno del neurodesarrollo progresivo, predominantemente en el sexo femenino que condiciona graves problemas neurológicos. En la mayoría de los casos está asociado a mutaciones del gen *MECP2*, generalmente existe un neurodesarrollo normal hasta la edad de 6–18 meses, cuando existe una regresión del lenguaje y de las habilidades adquiridas, aparición de movimientos estereotipados de las manos y microcefalia. El diagnóstico se confirma con estudios genéticos que revelan la afectación del gen *MECP2*; el tratamiento y seguimiento de los pacientes es multidisciplinario. **Objetivo**: Describir las características clínicas y exámenes de gabinete de paciente con síndrome de Rett en un hospital de tercer nivel. **Resumen Clínico del Caso Con Resultado de Estudios, Tratamiento Instaurado y Evolución**: Se trató de una paciente con seguimiento en la consulta externa con diagnóstico genético de síndrome de Rett. Paciente femenino de 7 años de edad, sin antecedentes heredofamiliares. Neurodesarrollo con sostén cefálico 7 meses, primeras palabras 8 meses, sedestación 12 meses, no gateo, bipedestación a los 20 meses sin lograr deambulación, alcanzando lenguaje de 10 palabras sin formar frases. A los 2 años de edad comienza con regresión del desarrollo, pérdida del lenguaje adquirido, movimientos estereotipados de lavado de manos, sialorrea frecuente; se deriva con el servicio de genética realizándose estudio genético (exoma) 07.08.2024 con variante patogénica en el gen MECP2 NM001110792.2c>T. Contando con tomografía de cráneo observándose con atrofia y electroencefalograma con actividad paroxística en terapia anticrisis. Actualmente paciente de custodia en seguimiento multidisciplinario. **Conclusiones**: El síndrome de Rett es una patología poco frecuente caracterizada por un neurodesarrollo normal hasta aproximadamente los 2 años de edad en la cual se sufre una regresión con pérdida de las habilidades adquiridas. La importancia de la detección temprana recae en la necesidad de valoraciones y seguimiento multidisciplinario; encontrándose aun en investigaciones las terapias dirigidas a la enfermedad.

**Palabras Clave:**

síndrome; neurodesarrollo; regresión; gen

**Lisencefalia E Hipoplasia Cerebelosa Vinculada a una Nueva Variante en el Gen *DCX*: Reporte de Caso**

Estrada-Gómez CG^1^, Rosas Hernández Jhonatan^2^, Espinosa-Zacarias JP^1^, Ruíz-Ferreira MS^1^

^1^Hospital Regional “Lic. Adolfo López Mateos” ISSSTE, 01030 Ciudad de México, México

^2^Universidad Autónoma de Tamaulipas, 87000 Ciudad Victoria, México

**Introducción**: La lisencefalia tipo 1 o clásica, frecuentemente asociada a variantes patogénicas en PAFAH1B1 (LIS1), presenta una prevalencia de 1:100.000 nacimientos. Las mutaciones en el gen DCX (doblecortina), ligadas al cromosoma X, provocan una migración neuronal anómala en varones ocasionando lisencefalia, generalmente con mejor pronóstico que las variantes en PAFAH1B1. Estos pacientes pueden presentar discapacidad intelectual, parálisis cerebral, epilepsia farmacorresistente y microcefalia postnatal, sin alteraciones cerebelosas, alcanzando en algunos casos la marcha independiente hacia los 7,5 años. Se reporta el primer caso clínico asociado a la variante DCX c.547C>T (p.Arg183Cys), homocigota sin sentido, previamente clasificada de significado incierto (ClinVar ID 158452), pero sin una descripción clínica en la literatura. **Objetivo**: Describir el caso clínico de un paciente pediátrico con esta variante. **Resumen Clínico del Caso Con Resultado de Estudios, Tratamiento Instaurado y Evolución**: Masculino de 2 años 4 meses, sin antecedentes familiares relevantes. Nace vía cesárea programada a las 39 SDG. Inicio de padecimiento a los 10 días de vida con crisis epilépticas (mioclonías palpebrales, chupeteo, hipertonía en extremidad superior derecha). Sin hallazgos etiológicos sintomáticos; perfiles TORCH y metabólico negativos. En tratamiento con levetiracetam. Retraso del neurodesarrollo. Posteriormente presencia de espasmos epilépticos en extensión y movimientos clónicos. Exploración neurológica: microcefalia, no sostén cefálico, sonidos guturales, sin respuesta a estimulo luminoso, no seguimiento visual, nistagmo horizontal, hipertonía generalizada, hiperreflexia global, clonus bilateral. Alimentación por gastrostomía. EEG: paroxismos de punta, polipunta y complejos punta onda lenta en regiones frontocentrales bilaterales de predominio izquierdo. Resonancia magnética de encéfalo agiria anteroposterior bilateral, agenesia del cuerpo calloso, hipoplasia cerebelosa, quiste retrocerebeloso bilateral y ventriculomegalia. Actualmente en tratamiento anticrisis a base de VPM, TPM, VGB, OXC, CLB con control parcial. **Conclusiones**: Este es el primer caso clínico reportado con la variante DCX c.547C>T, la cual se reclasifica como probablemente patógena, dada su correlación clínica, múltiples anomalías anatómicas en encéfalo y pobre evolución neurológica, en contraste con otras variantes previamente descritas en DCX.

**Palabras Clave:**

lisencefalia; DCX; cerebelo; agiria; epilepsia

**Encefalopatía Necrotizante Como Complicación de Dengue Grave: Reporte de Caso**

Cruz Rios Vania Kenaí^1^, Herrera Mora Patricia^1,2^, Mayorga Chávez Gabriela Carolina^1^

^1^Instituto Nacional de Pediatría (INP), 04530 Ciudad de México, México

^2^Universidad Nacional Autónoma de México (UNAM), 04510 Ciudad de México, México

**Introducción**: El virus del dengue (DENV) es un virus neurotrópico que causa daño al SNC ya sea por vía directa o indirecta a través de la producción de autoanticuerpos. Causa cefalea (90%), y otras manifestaciones menos comunes como meningitis, miositis, mielitis, encefalomielitis diseminada aguda, neuritis óptica, síndrome de Guillain Barré y en raras ocasiones, encefalopatía aguda necrotizante. **Objetivo**: Se reporta un caso de paciente escolar con encefalopatía aguda necrotizante asociada a infección por DENV. **Resumen Clínico del Caso Con Resultado de Estudios, Tratamiento Instaurado y Evolución**: Femenino de 8 años, residente de Oaxaca, previamente sana. Inicia 04 de agosto con mialgias, artralgias, dolor retroocular y 24 horas después fiebre persistente, ataxia, irritabilidad, confusión, agresividad, bradipsiquia, bradilalia y crisis epilépticas tońico-clońicas generalizadas con evolución a estado epiléptico, recibe tratamiento con fenitoína, levetiracetam e infusión con midazolam. La TAC cerebral presenta hipodensidad simétrica en talamos. Se sospecha neuroinfección contra encefalitis autoinmune, se indica antibiótico y antiviral. Por el antecedente epidemiológico se pide PCR para DENV. Con evolución tórpida por lo que se inicia esteroide y posteriormente se administra Gammaglobulina intravenosa 2 gramos/kg con importante mejoría. Paraclínicos: anemia leve (12,8 gramos) y trombocitopenia (99.000 plaquetas), PCR sérico positivo para DENV serotipo 3. Punción lumbar: Proteinorraquia leve (48 mg/dL), ausencia de microorganismos. EEG: severa lenificación generalizada en rango delta. RM cerebral zonas de destrucción hemorrágica a nivel de tálamos bilateral, simétrica, con restricción a la difusión y halo de hemosiderina. **Conclusiones**: La encefalopatía necrotizante aguda (ANEC) vinculada al dengue es una complicación neurológica poco común y severa. Se presenta como una encefalopatía de rápida evolución que surge tras un cuadro febril viral. Los pacientes manifiestan deterioro temprano acompañado de cefalea, crisis epilépticas y petequias. La RMC muestra lesiones hemorrágicas en tálamos bilateral como en este caso. El tratamiento adecuado incluye inmunomodulación con metilprednisolona intravenosa y, en algunos casos, inmunoglobulina intravenosa.

**Palabras Clave:**

dengue; encefalopatía necrotizante; reporte de caso

**Curso Clínico de la Epilepsia Refractaria Secundaria a Displasia Cortical Focal en Pacientes Manejados con Tratamiento Médico Exclusivo Versus Aquellos Sometidos a Cirugía de Epilepsia: Serie de Casos**

Mayorga Chávez Gabriela Carolina^1^, Herrera Mora Patricia^1^

^1^Instituto Nacional de Pediatría, 04530 Ciudad de México, México

**Introducción**: La displasia cortical focal es una causa frecuente de epilepsia refractaria en edad pediátrica. En casos seleccionados, la cirugía de epilepsia puede ofrecer control significativo de crisis. Comparar la evolución clínica en pacientes con manejo médico versus quirúrgico permite valorar el impacto del tratamiento sobre la frecuencia de crisis y la carga farmacológica. **Objetivo**: Describir y comparar el curso clínico de la epilepsia refractaria en pacientes con displasia cortical focal tratados médicamente frente a aquellos sometidos a cirugía de epilepsia. **Resumen Clínico del Caso Con Resultado de Estudios, Tratamiento Instaurado y Evolución**: Material y Métodos: Serie de casos retrospectiva. Se analizaron 10 pacientes con epilepsia refractaria secundaria a displasia cortical focal, evaluados entre 2018 y 2024 en el Instituto Nacional de Pediatría. Se documentaron edad de inicio, tipo y frecuencia de crisis, tratamiento antiepiléptico, cirugía, electroencefalograma pre y postoperatorio, y evolución clínica. **Resultados**: Tres pacientes fueron sometidos a cirugía, ya sea hemisferectomía o resección focal, con una edad promedio de intervención de siete años. Todos presentaban crisis epilépticas focales de alta frecuencia, con hasta quince episodios diarios antes del procedimiento. Posteriormente, dos de ellos lograron un control total de las crisis durante un periodo superior a tres años, con una reducción notable en la cantidad de fármacos antiepilépticos utilizados, pasando de tres o cuatro medicamentos a uno solo. En el grupo que no fue intervenido quirúrgicamente, compuesto por siete pacientes, la epilepsia persistió con frecuencia variable, desde tres episodios mensuales hasta diez por día, lo que requirió el uso de múltiples fármacos. Los estudios electroencefalográficos previos a la cirugía mostraron actividad epileptiforme focal o generalizada. En dos de los casos operados, el EEG posterior no evidenció actividad epileptiforme. **Discusión**: La evolución clínica de los pacientes intervenidos fue favorable en términos de control de crisis y carga farmacológica, en comparación con aquellos tratados exclusivamente de forma médica, quienes mantuvieron epilepsia activa. La edad temprana de intervención se relacionó con mejores desenlaces. Los hallazgos de esta serie son compatibles con lo reportado en la literatura internacional. **Conclusiones**: La cirugía representa una opción terapéutica eficaz en epilepsia refractaria por displasia cortical focal.

**Palabras Clave:**

epilepsia refractaria; displasia cortical focal; cirugía de epilepsia; fármacos anticrisis

**Un Síndrome de Pierpont Llegó a Edad Adulta**

Roman Islas Leslie Marielle^1^, Delgado Ochoa Azucena^1^

^1^Hospital General de Tijuana, 22000 Tijuana, México

**Introducción**: El síndrome de Pierpont es una anormalidad genética rara que se caracteriza por retrasos del desarrollo, discapacidad intelectual, deformidad facial, hipotonía, microcefalia, “almohadillas” de tejido blando en palmas y plantas. Las mutaciones patogénicas en el gen *TBL1XR1* fueron descritas por primera vez en 2012 en dos pacientes con trastorno del espectro autista (TEA). La herencia genética molecular es un trastorno autosómico dominante en el cual se ha descrito como factor de riesgo la edad paterna avanzada. Se ha reportado herencia recesiva ligada al cromosoma X. **Objetivo**: Describir las manifestaciones clínicas y evolución de un paciente con síndrome de Pierpont. **Resumen Clínico del Caso Con Resultado de Estudios, Tratamiento Instaurado y Evolución**: Masculino de 19 años. Padres sanos, no consanguíneos. Padre contacto con fumigantes. Producto de la gesta 3 embarazo y parto sin complicaciones. Hospitalizaciones por descontrol de crisis, gastrostomía y funduplicatura. Al año de edad paro cardiorrespiratorio (durante realización de resonancia magnética). Inició a los 3 meses de edad con sacudidas esporádicas de extremidades. A los 5 meses espasmos tónicos y distonías, evolucionando a crisis focales y generalizadas, gelásticas, dacrísticas, tónicas axiales, ausencias atípicas, mioclonías. Desde los 3 años sueño intermitente no más de 3 horas continuas. Ha recibido fenitoína, fenobarbital, ácido valproico, levetiracetam, gabapentina, haldol, risperidona, prednisona, topiramato, diazepam, clobazam, clonazepam, cannabidiol, primidona, gabapentina, perampanel, etosuximida, lacosamida sin llegar al control reportándose hasta 40 crisis epilépticas durante el día. Tratamiento actual valproato de magnesio, topiramato, brivaracetam, presentando aun 1–3 crisis epilépticas por día. Exploración: Peso 16,1 kg, Talla. 155 cm. Hipoactivo, hiporeactivo, caquéctico. Microcefalia, sin sostén cefálico, opacidad conjuntival, nistagmos horizontal, hipotonía, limitación a la extensión de extremidades, reflejos osteotendinosos 0, escoliosis. Exoma clínico (12/09/2019) Variante *De novo* TBL1XR1.Electroencefalograma (5/02/2021). Puntas y ondas agudas intermitentes de mayor amplitud fronto temporal. **Conclusiones**: Se enfatiza la relevancia de la investigación continua para mejorar el diagnóstico y la comprensión del síndrome de Pierpont.

**Palabras Clave:**

síndrome de Pierpont; lipomatosis plantar; encefalopatía epiléptica; alteración cognitiva; retraso psicomotor; síndrome dismórfico; epilepsia de difícil control; TBL1XR

**Espectro Clínico de Trastornos Neurológicos Relacionados a Atp1a3. Presentación de Tres Casos Clínicos**

Pérez Rodríguez Juan Steven^1^, Vázquez Montantes José^1^, Bravo Oro Antonio^1^

^1^HRAE Dr. Ignacio Morones Prieto, 78290 San Luis Potosí, México

**Introducción**: Antecedentes: Los trastornos relacionados a variante en ATP1A3, son un grupo clínicamente heterogéneo, agrupándose en tres tipos: (1) hemiplejía alternante, (2) distonía-parkinsonismo de inicio rápido y (3) síndrome de CAPOS (ataxia cerebelosa, arreflexia, pie cavo, atrofia óptica y pérdida auditiva neurosensorial). ATP1A3 codifica la a3 Na+K+-ATPasa expresada principalmente en núcleos de la base, sustancia negra, tálamo, cerebelo, núcleo rojo, núcleo oculomotor, núcleo retículo-tegmental de la protuberancia e hipocampo, en ubicación conjunta con neuronas GABAérgicas. **Objetivo**: Se presentan 3 casos con mutaciones en el gen *ATP1A3*. **Resumen Clínico del Caso Con Resultado de Estudios, Tratamiento Instaurado y Evolución**: Caso 1: Femenina 2 años, a los 15 días de vida crisis focales motoras, se inicia levetiracetam con buena respuesta, a los 4 meses eventos de nistagmo transitorio y hemiplejía transitoria alternante de duración de horas a días asociada al baño, iniciaron oxcarbazepina sin mejoría. Se agrega flunarizina sin mejoría, posteriormente prednisolona logrando estar hasta un mes sin eventos de hemiplejia. Caso 2: Masculino de 1 año. A los cuatro meses crisis focales motoras refractarias con polifarmacia, valorado en nuestro servicio al año, retraso del desarrollo psicomotor, eventos de nistagmo transitorio que cede espontáneamente y hemiplejía alternante que duran horas, con la flunarizina disminuyen los eventos y un año sin crisis convulsivas. Caso 3: Recién nacido masculino con crisis convulsivas motoras, multifocales de difícil control, requiriendo hasta fármacos, tamiz metabólico ampliado negativo. Múltiples internamientos por estado epiléptico, se inicia esteroide a dosis bajas disminuyendo los eventos convulsivos. Resultados: Caso 1: Resonancia magnética normal, mutación patológica heterocigota c.2443G>A (p.Glu815Lys). Caso 2: Resonancia magnética con pérdida de volumen cortical, mutación patológica heterocigota c.2411C>T (p. Thr804Ile). Caso 3: Resonancia magnética con ventriculomegalia y disminución cortical, mutación patológica heterocigota c.2759A>C (p.Gln920Pro). Conclusiones: Variantes patogénicas en heterocigosis en ATP1A3 están asociadas a cuadros variables como lo presentamos con nuestros casos y lo complejo del tratamiento.

**Palabras Clave:**

ATP1A3; epilepsia; hemiplejía alternante; trastornos de movimiento; resonancia magnética de cráneo

**Astrocitoma Pilomixoide en Edad Pediatrica: Reporte de Caso**

Batista González Leidy Rosanny^1^, Cantú Salinas Adriana Carlota^1^, Carrión García Ana Luisa^1^, Vázquez Fuentes Salvador^1^, De la Garza Pineda Oscar^1^, Chávez Luévanos Beatriz Eugenia^1^

^1^Servicio de Neurología, Hospital Universitario “Dr. José Eleuterio González”, 64460 Monterrey, México

**Introducción**: El astrocitoma pilomixoide (APM), es una entidad clínico-patológica propia, con incidencia menor al 1% de los astrocitomas, con preferencia topográfica hipotálamo-quiasmática, diseminación por líquido cefalorraquídeo (LCR), alto grado de recidiva y mortalidad. La mayoría de los tumores exhiben fusiones o alteraciones BRAF V600E, esta mutación se ha descrito como un biomarcador de pronóstico desfavorable por mínima respuesta a quimioterapia. **Objetivo**: Describir el reporte de caso de astrocitoma pilomixoide en edad pediátrica. **Resumen Clínico del Caso Con Resultado de Estudios, Tratamiento Instaurado y Evolución**: Femenina de 17 meses con retraso de neurodesarrollo motor grueso y personal social desde los 8 meses, seguimiento en pediatría y gastroenterología pediátrica por poca ganancia ponderal diagnosticada en febrero 2024; a los 13 meses progreso a desnutrición severa en mayo del 2024, agregándose aumento del perímetro cefálico (PC) de 2,5 cm en 1 mes; junio 2024, 2 semanas previas a su ingreso inicio regresión progresiva del neurodesarrollo en área de lenguaje, personal social, motor fino, grueso; deshidratación severa, disfagia progresiva, iniciando a sólidos hasta líquidos. Al ingreso neurológicamente FOUR: 14, PC 48,5 cm, irritabilidad, braquicefalia; hipotrofia, hipotónica e hiperreflexia globalmente. La tomografía craneal (TC), evidenció: masa supraselar hipotalámica, hidrocefalia activa y obstrucción del III ventrículo. Se colocó válvula de presión media de derivación ventrículo peritoneal. La resonancia magnética (RM) mostró: lesión extraaxial, de bordes definidos, de componente sólido y quístico. Se abordó con craneotomía, resecando 60% de la lesión. La evolución fue remisión de irritabilidad e hiperreflexia global, persistiendo hipotonía central e hipotrofia. Tras 2 meses post resección, hipertonía global con hiperreflexia de miembros inferiores y, en octubre del 2024, se realizó TC control que mostró: lesión residual sin modificación respecto al previo. Actualmente, el paciente está recibiendo quimioterapia con vinblastina. El paciente cumplió con los criterios: antígeno Ki67 de crecimiento tumoral de 10%, de mal pronóstico, sinaptofisina, producida por células endocrinas y Olig2 demostrando ser un glioma, acompañado de matriz mixoide, permitiendo inclinarse al diagnóstico de APM. **Conclusiones**: Aparte de las pruebas inmunohistológicas, se deben realizar pruebas moleculares para planificar el tratamiento y mejorar el pronóstico

**Palabras Clave:**

astrocitoma pilomixoide; glioma; pruebas moleculares

**Tuberculosis de Sistema Nervioso Central**

Montoya Beltrán Andreé Monserrat^1^, Maldonado Ontiveros Dianey Jacqueline^1^, González Nava Darío Roberto^1^

^1^Hospital Civil de Tepic, 63000 Tepic, México

**Introducción**: La tuberculosis (TB) del sistema nervioso central (SNC) es una de las formas más temidas de tuberculosis, puede presentarse en forma de tuberculoma o meningoencefalitis, su agente etiológico es Mycobacterium Tuberculosis. En Nayarit, en 2022 se registraron 7 casos en menores de 5 años. **Objetivo**: Describir el abordaje y tratamiento de preescolar con TB de SNC. **Resumen Clínico del Caso Con Resultado de Estudios, Tratamiento Instaurado y Evolución**: Femenino de 5 años, con padecimiento caracterizado por cefalea, fiebre, disartria y crisis epilépticas de evolución aguda, inmunizaciones completas y combe positivo. A la exploración se corrobora presencia de irritabilidad, parálisis facial izquierda con hemiparesia contralateral. Tomografía de cráneo simple con lesión hipodensa en lóbulo frontoparietal izquierdo, con edema perilesional. La resonancia magnética de encéfalo simple y contrastada presencia de lesión previamente descrita además de lesión de características similares en lóbulo temporal derecho y múltiples nódulos a nivel infra y supratentorial, evidencia de reforzamiento meníngeo a nivel interhemisférico, supraventricular, y del lóbulo temporal izquierdo. Líquido cefalorraquídeo reporta Polimorfonucleares 59%, Mononucleares 41%, Gram, cultivo, y gen xpert: negativo, tinción Ziehl Neelsen: positiva. se instaura manejo con antifímicos y mejoría clínica hasta su egreso. Las neuroinfecciones representan un reto diagnóstico debido a lo inespecífico de los síntomas, como cefalea, fiebre hasta crisis epilépticas de primera vez, aunado a focalidad neurológica. El abordaje incluye estudios de neuroimagen y microbiológicos para guiar el tratamiento, teniendo en cuenta la epidemiología local como lo es en este caso. **Conclusiones**: La heterogeneidad de los síntomas de TB SNC varía de acuerdo a la localización de las lesiones, complicando inicialmente el diagnóstico, orientar el abordaje propicia un diagnóstico certero para el tratamiento temprano, mejorar el pronóstico y limitar las secuelas neurológicas.

**Palabras Clave:**

tuberculosis en pediatría; tuberculosis sistema nervioso central; tuberculosis meníngea

**Encefalitis Necrotizante Aguda en un Hospital de Tercer Nivel: Reporte de Caso**

Sánchez López Sergio Lisandro^1^, Ulloa Carrillo Marysol^1^, Cordero Robles Gerardo Enrique^1^

^1^UMAE Hospital de Pediatría CMNO, 44340 Guadalajara, México

**Introducción**: La encefalitis necrotizante aguda es una enfermedad poco frecuente de curso rápido con mal pronóstico neurológico, que causa deterioro neurológico, encefalopatía y en la mayoría de las veces crisis epilépticas. Generalmente se presenta posterior al curso de un cuadro infeccioso; con elevación de TNF-alfa e Interleucina 6 creando una tormenta de citocinas, las causantes de la inflamación y posterior necrosis tisular. Existen mutaciones en el gen *RANBP2* que se pueden asociar con asociaciones familiares. La resonancia magnética demuestra lesiones que son simétricas y multifocales, siendo característica la afectación talámica de manera bilateral. Se han reportado casos de respuesta favorables al uso de tocilizumab, anticuerpo monoclonal que actúa bloqueando la interleucina 6. **Objetivo**: Describir las características clínicas y exámenes de gabinete de paciente con encefalitis necrotizante aguda en un hospital de tercer nivel. **Resumen Clínico del Caso Con Resultado de Estudios, Tratamiento Instaurado y Evolución**: Se trató de un caso de encefalitis necrotizante aguda en un paciente pediátrico. Paciente masculino de 7 años de edad, sin antecedentes previos de importancia, quien inicia con sintomatología gastrointestinal con diagnóstico de dengue, 1 semana posterior con cuadro de encefalopatía, crisis convulsivas; intubación endotraqueal por deterioro neurológico con estancia en unidad de terapia intensiva, se realiza resonancia magnética con lesiones hiperintensas en secuencia T2 y FLAIR a nivel de lóbulos temporales, sustancia blanca subcortical y yuxtacortical, talamos bilaterales, cerebelo y tallo cerebral. Se integra cuadro de encefalitis necrotizante aguda, se administra terapia con esteroide sistémico y tocilizumab, egresado con secuelas caracterizadas por hemiparesia y espasticidad de hemicuerpo izquierdo. **Conclusiones**: La encefalitis necrotizante aguda es un cuadro de inicio agudo, de progresión rápida y de carácter monofásico, en bibliografías se reporta con mortalidad de hasta el 30% y de los sobrevivientes con marcadas secuelas neurológicas. Por eso la importancia de la detección temprana y tratamiento oportuno para el inicio de este.

**Palabras Clave:**

encefalitis; citocinas; autoinmune

**Neurofibromatosis Tipo 1 y Neurofibroma Plexiforme Gigante. Reporte de un Caso**

Méndez Fernández Daniela^1^, Torres Damián Yereth^1^, Caballero Navarro Yael^1^, Rosas Contreras Miguel Ángel^1^, Cebada López Flora^1^

^1^Instituto Mexicano del Seguro Social UMAE Hospital General “Dr. Gaudencio González Garza”, Centro Médico Nacional la Raza, 02990 Ciudad de México, México

**Introducción**: La neurofibromatosis, el síndrome neurocutáneo más común, dividido en neurofibromatosis tipo 1 (Von Recklinghausen) y tipo 2. La NF-1 ocurre en 1 por cada 4000 nacimientos y se considera autosómica dominante con penetrancia de 80–90%. El tumor más comúnmente encontrado en los pacientes con NF-1 son los neurofibromas, los cuales pueden afectar una raíz nerviosa o el plexo completo, y se presentan como un engrosamiento mal delimitado, irregularmente cilíndrico, fusiforme o nodular, y tienen cierto potencial de malignización. Involucran nervios profundos y grandes, y muestran fascículos irregulares como con secuencia del incremento de la matriz endoneural y del perineuro. **Objetivo**: Descripción de un paciente con Neurofibromatosis tipo 1 con presencia de neurofibroma plexiforme gigante. **Resumen Clínico del Caso Con Resultado de Estudios, Tratamiento Instaurado y Evolución**: Femenino de 7 años de edad, con antecedente de madre de 33 años con sospecha de neurofibromatosis tipo 1. Exploración neurológica alteraciones en lenguaje, discalculia; 100 manchas café con leche, efélides en región axilar, lesión de localización en extremidad inferior izquierda con diseminación a región pélvica, con involucro de labio mayor ipsilateral y clítoris, con piel hiperpigmentada, Dolorosa a la sedestación y deambulación. Biopsia (04.01.24): tumor multilobulado, nodular, afecta músculo estriado. Con resonancia magnética (04.06.24) lesión amorfa de bordes lobulados, heterogénea, de localización peritoneal que involucra espacios infracólico, paravesical, pararrectal de manera bilateral, rectouterino, vesicouterino, de retzius y presacro, compresión de vejiga, sigmoides y asas de intestino delgado, compromiso genital izquierdo. Catalogado como sintomático e inoperable. En manejo con gabapentina con mejoría del dolor, con impacto en funcionalidad. **Conclusiones**: Un neurofibroma plexiforme es una neoplasia que afecta múltiples fascículos de un nervio, ocasionando la compresión de estructuras adyacentes. La transformación maligna ocurre hasta en 17% de los casos, por lo cual la importancia vigilancia y tratamiento multidisciplinario oportuno. El manejo quirúrgico de estas lesiones se dificulta frecuentemente por la complejidad anatómica de los tumores y estructuras afectadas. El tratamiento para neurofibromas inoperables sigue siendo un reto clínico, sin embargo, se ha demostrado mejoría clínica con selumetinib, el mesilato de imatinib y el interferón alfa-2b los cuales encuentran propuestos como tratamiento en nuestra paciente.

**Palabras Clave:**

neurofibromatosis tipo 1; neurofibroma plexiforme

**Síndrome Cerebro-Renal Birk-Landau-Perez**

Ledezma Flores Elias^1^, Delgado Ochoa Martha Azucena^2^, Carolina Estefanía Herrera Cabadas^2^

^1^Universidad Xochicalco, 22100 Tijuana, México

^2^Hospital General de Tijuana, 22010 Tijuana, México

**Introducción**: El síndrome Birk–Landau–Pérez (BILAPES) es un trastorno autosómico recesivo asociado al gen *SLC30A9*, que codifica para proteínas transportadoras de zinc (ZnT-9). Descrito en 2017, caracterizado por deterioro neurológico de inicio temprano, pérdida auditiva, discapacidad intelectual, apraxia oculomotora, nefritis tubulointersticial e hipertensión. La alteración en las concentraciones de zinc en el sistema nervioso se manifiesta con espasticidad, discinesias, hipertonía de extremidades y alteraciones de la marcha. **Objetivo**: Dar a conocer otra causa genética no identificada en epilepsia de difícil control. **Resumen Clínico del Caso Con Resultado de Estudios, Tratamiento Instaurado y Evolución**: Femenino de 16 meses, originaria de Tijuana. Hija única de padres sanos, no consanguíneos, embarazo normoevolutivo 41 SDG, parto eutócico, peso 2700 g. Consulta por crisis epilépticas desde el período neonatal, microcefalia y agenesia del cuerpo calloso. Antecedentes de sangrado de tubo digestivo, catarata congénita, displasia de cadera derecha, hipoplasia renal derecha. Hospitalizaciones por tres estados epilépticos. Gastrostomía y traqueostomía (a los 13 meses). Hipertensión arterial en tratamiento con prazosina y nifedipina. Frecuencia de crisis epilépticas cada mes, focales componente motor tónico de menos de 1 min de duración. En tratamiento con levetiracetam y fenobarbital. Exploración: Peso 5 kg (<P1), talla 62 cm (<P1), IMC 13,1 (<P1), perímetro cefálico 34 cm (<P1). Microcefalia, fontanela anterior puntiforme, microoftalmia, úvula hendida, micrognatia, pabellón auricular izquierdo alado y cuello corto. Reactiva, irritable, hipotonía axial, hipertonía periférica, trofismo disminuido, hiperreflexia +++, ausencia de sostén cefálico, grasping, respuesta plantar flexora bilateral. Electroencefalograma: Puntas y punta onda lenta en regiones central y temporal derechas. Exoma clínico: Variantes heterocigotas SLC30A9 (p.Gly262Valfs*22 y p.Asp497Val). **Conclusiones**: BILAPES es un trastorno ultrarraro con manifestaciones multisistémicas desde etapas tempranas. Enfatizamos una mayor conciencia sobre enfermedades raras y la importancia en la identificación clínica, investigación y confirmación genética para mejorar el diagnóstico y tratamiento.

**Palabras Clave:**

síndrome cerebro-renal; SLC30A9; BILAPES; transportador de zinc; epilepsia

**Coma Persistente Secundario a Meningoencefalitis por Virus Herpes 7 en Paciente Pediátrico Inmunocompetente**

Narváez González Diana Elisa^1^, Márquez Villaseñor Lennin Antonio^1^, Cantú Salinas Adriana Carlota^1^, Carrión García Ana Luisa^1^, Vázquez Fuentes Salvador^1^, De la Garza Pineda Oscar^1^, Chávez Luévanos Beatriz Eugenia^1^

^1^Servicio de Neurología Pediátrica, Hospital Universitario “Dr. José Eleuterio González”, Universidad Autónoma de Nuevo León, 64460 Monterrey, México

**Introducción**: La infección primaria por VHH-7 (Virus Herpes Humano) conocida como “la sexta enfermedad”, está presente en casi el 95% de los adultos debido a una infección previa, permaneciendo de forma persistente; sin embargo, los niños, adquieren seropositividad de los anticuerpos maternos durante el primer año de vida, alcanzando un 53–67% a los 2 años. La transmisión se ha descrito través de fluidos corporales infecciosos, como la saliva y las secreciones respiratorias. **Objetivo**: Describir la evolución clínica y desenlace de pediátrico inmunocompetente con meningoencefalitis secundaria a HHV-7. **Resumen Clínico del Caso Con Resultado de Estudios, Tratamiento Instaurado y Evolución**: Paciente de 2 años 3 meses de edad, sin antecedentes familiares ni perinatales de importancia. Neurodesarrollo adecuado para la edad por Denver II, inmunizaciones incompletas, cuadro de vías aéreas superiores 2 semanas previas a su ingreso, tratada con analgésicos y antihistamínicos sin remisión. Inicia padecimiento 7 días previos, con disminución en el estado de alerta, alternando con irritabilidad y somnolencia, vómito en proyectil, intolerancia a la vía oral, fiebre de hasta 40 °C, deshidratación severa, cefalea holocraneana de intensidad 8/10, estatus epiléptico caracterizado por crisis tónicas. A la exploración física FOUR 12 puntos, nervios craneales sin alteración, fuerza muscular 4/5, reflejos de estiramiento muscular +++ global, Brudzinski presente y respuesta plantar extensora bilateral, sin manifestaciones cutáneas. Se realiza punción lumbar con parámetros bioquímicos compatibles a neuroinfección y tomografía computada con hidrocefalia activa. Electroencefalográficamente con actividad delta, de baja amplitud, asimétrico sin actividad ictal y detección de VHH-7 en panel viral de LCR. Con posterior necesidad de craniectomía descompresiva de urgencia. **Conclusiones**: Las meningoencefalitis por VHH-7 es una emergencia neurológica donde el diagnóstico y tratamiento empírico desciende la mortalidad hasta 10%, en cambio el retrasar la administración la incrementa hasta un 70%, el haber recibido atención médica al inicio del cuadro pudo haber modificado la historia natural de la enfermedad, evitando el proceso de coma persistente y posteriormente la muerte.

**Palabras Clave:**

meningoencefalitis; virus; herpes 7; pediátrico

**Encefalopatía Hiperamonémica Inducida por Valproato en un Adolescente con Epilepsia Refractaria: Reporte de Caso**

Arianna Paola Peiro-Sánchez^1^, Antonio Bravo-Oro^1^, Jorge Luis Garcia-Ramírez^1^

^1^Departamento de Neurología Pediátrica HRAE “Dr. Ignacio Morones Prieto”, 78290 San Luis Potosí, México

**Introducción**: La encefalopatía hiperamonemica inducida por valproato (EHV) es una entidad clínica poco frecuente pero potencialmente grave, especialmente en población pediátrica con epilepsia refractaria. Se caracteriza por deterioro neurológico agudo o subagudo, incluyendo alteraciones del estado de conciencia, ataxia, convulsiones, letargia e incluso coma, sin necesidad de disfunción hepática evidente ni niveles tóxicos del fármaco. El valproato interfiere en el ciclo de la urea al inhibir la enzima carbamilfosfato sintetasa I, secundario a la reducción de N-acetilglutamato, y disminuye los niveles de carnitina, lo que contribuye a la acumulación de amonio sérico. Los factores de riesgo incluyen el uso combinado de anticonvulsivantes, dosis elevadas de valproato, deficiencia de carnitina, trastornos metabólicos subyacentes y edades extremas. **Objetivo**: Se presenta el caso de un paciente masculino de 14 años con epilepsia refractaria de etiología no determinada, en tratamiento con valproato de magnesio, topiramato, clonazepam y cannabidiol con alteración del estado de conciencia. **Resumen Clínico del Caso Con Resultado de Estudios, Tratamiento Instaurado y Evolución**: Paciente que Ingresa al servicio de urgencias con somnolencia, múltiples crisis convulsivas tónico-clónicas, y deterioro progresivo del estado de alerta. Se documentaron 11 crisis en 48 horas, con intolerancia a la vía oral. Se sospechó estado epiléptico no convulsivo, confirmado mediante electroencefalograma. Durante su evolución se detectó hiperamonemia severa (346 µmol/L), con niveles terapéuticos de valproato y pruebas de función hepática normales. Ante la sospecha de EHV, se suspendió el valproato y se instauró tratamiento con dieta hipoproteica, L-carnitina, cisteína y L-ornitina-L-aspartato. La evolución clínica fue favorable, con recuperación progresiva del estado de conciencia, reintroducción de alimentación oral y control adecuado de las crisis convulsivas. Al egreso, el paciente se encontraba clínicamente estable, deambulando con ayuda mínima. **Conclusiones**: La EHV debe considerarse como diagnóstico diferencial en todo paciente con epilepsia que presente un deterioro neurológico agudo, independientemente de los niveles séricos de valproato y función hepática. La identificación temprana y la suspensión oportuna del fármaco, junto con el manejo metabólico adecuado, son fundamentales para evitar secuelas neurológicas permanentes.

**Palabras Clave:**

hiperamonemia; encefalopatía metabólica; estado epiléptico no convulsivo; ciclo de la urea; valproato de magnesio

**Distrofia Miotónica Tipo 1, Asociado a Alteración de Gen *DMPK*, Reporte de Caso**

Cristian A. Marrufo Suarez^1^, Mario González Medina^1^, Pablo Valladares Sánchez^1^

^1^Servicio de Neurología Pediátrica, Hospital Regional de Alta Especialidad del Niño Dr. Rodolfo Nieto Padrón, 86030 Villahermosa, México

**Introducción**: La distrofia muscular miotónica tipo 1 o enfermedad de Steinert es una enfermedad de baja prevalencia (<5/10.000) con penetrancia casi completa y daño multiorgánico. Es una enfermedad autosómica dominante producida por la expansión de tripletes CTG en la región no codificante del gen DMPK, localizada en el brazo largo del cromosoma 19 (19q13.3). Es una enfermedad con alteraciones neuromusculares, pero que produce también alteraciones sistémicas. **Objetivo**: Presentación de un caso de Síndrome de Steinert, así como sus características. **Resumen Clínico del Caso Con Resultado de Estudios, Tratamiento Instaurado y Evolución**: Antecedentes: Paciente masculino, madre de 36 años y padre de 41 años, ambos sanos. Hermano sin patologías. Sin antecedentes familiares de importancia. Vacunas completas. Sin antecedentes prenatales relevantes. Desarrollo neurológico aparentemente normal hasta los 7 años, presentó dificultades del aprendizaje. A los 10 años, es remitido a la consulta de Neurología por diagnóstico de TDAH, asociado al inicio de sintomatología motora. Se observa debilidad muscular, eventos miotónicos durante actividades que requieren esfuerzo físico y fatigabilidad. Ante la sospecha de síndrome miotónico, se realiza derivación a diferentes especialidades: Cardiología (por PR prolongado en seguimiento), Neumología (por disfagia ocasional y patrón restrictivo), Neuropsicología y Genética. Actualmente en manejo con fenitoína y metilfenidato. **Conclusiones**: La enfermedad presenta una edad de aparición muy variable, desde la etapa prenatal hasta la edad adulta, con manifestaciones clínicas heterogéneas. La forma infantil (inicio entre 1 y 10 años) que se manifiesta por un trastorno del aprendizaje (principalmente lenguaje, escritura y lectura), trastornos de atención y un síndrome disejecutivo. Los niños muestran signos de fatiga y de hipersomnia. Generalmente la miotonía y los problemas musculares son de aparición tardía. El caso presentado es compatible con una variedad infantil, por la edad de inicio y los datos encontrados, el manejo de acuerdo a literatura es sintomático, en el caso del paciente con síntoma predominante motor (miotonía), la mexiletina es el medicamento de elección sin embargo fuera del mercado en México por lo que se utiliza fenitoína como alternativa, con adecuada respuesta, y evolución clínica.

**Palabras Clave:**

distrofia miotónica tipo 1; gen *DMPK*; distonía; miotonía; mutación genética; síndrome de Steinert

**Sindrome de Zhu-Tokita-Takenouchi-Kim Presentacion de un Caso**

Tejeda Guzmán Zuleyki Eugenia^1^, Cornelio Nieto José Ovidio^1^, González Medina Mario^1^

^1^Hospital Regional de Alta Especialidad del Niño Dr. Rodolfo Nieto Padrón, 86030 Villahermosa, México

**Introducción**: Síndrome causado por mutación heterocigota en el gen *SON* (182465) en el cromosoma 21q22. Con múltiples anomalías congénitas poco frecuente, caracterizado por retraso psicomotor, discapacidad intelectual y dismorfia facial de leve a moderada asociado a malformaciones cerebrales variables (paquigiria, ventriculomegalia, sustancia blanca e hipoplasia del cuerpo calloso y hemisferios cerebelosos), anomalías musculoesqueléticas, afectación ocular e hipotonía. Crisis epilépticas, malformaciones urogenitales, talla baja, defectos cardíacos y malformaciones gastrointestinales. **Objetivo**: Identificar este síndrome, a la fecha solo se han registrado 60 casos. Por lo que, ante datos clínicos, se debe pensar. **Resumen Clínico del Caso Con Resultado de Estudios, Tratamiento Instaurado y Evolución**: madre 35 años, padre 38 años, sanos sin antecedentes ni neurodegenerativos, hermano gemelo dicigótico sano, femenina de 14 años sano. Embarazo normo evolutivo, cesárea a las 35 semanas por preeclampsia, obtienen gemelar dicigóticos, siendo el paciente #1, con llanto débil, peso 1900 gramos, Apgar, talla, PC NR. egresan en binomio. herniorrafía inguinal derecha a los 3 meses de vida, sostén cefálico 1año. Balbuceo 4 años, monosílabos 6 años, bisílabos 9 años. Sedestación 1ª 3 meses, bipedestación 7 años, deambulación 9 años. Paciente de 11 años de edad, frente prominente, ojos hundidos, baja implantación auricular, hipertelorismo mamario, micropene. Hipotonía y fuerza proximal disminuida. En RM de encéfalo simple poca diferenciación de sustancia blanca en regiones parietales y occipitales, desmielinización en estas áreas, porción posterior de ambos centros semiovales. Quiste en cisterna magna, hipoplasia del vermis inferior. EEG normal. EMG. Neuropatía de nervio peroneo superficial y cubital bilateral con degeneración axonal. Rx tórax cardiomegalia, leve crecimiento aurícula derecha. Escoliosis dorsal. **Conclusiones**: Este caso destaca la importancia de considerar síndromes genéticos raros como la mutación en el gen *SON* al evaluar a pacientes con retraso psicomotor, discapacidad intelectual y dismorfias faciales. La identificación temprana de esta mutación puede mejorar los pronósticos del paciente al permitir un manejo integral. Este caso también enfatiza la necesidad de investigaciones adicionales para comprender mejor las características clínicas de este síndrome.

**Palabras Clave:**

síndrome ZTTK; mutación en el gen *SON*

**La Maldición de Ondina: De la Mitología a la Realidad. Presentación de un Caso**

Marrufo Suarez Cristian A^1^, González Medina Mario^1^, Valladares Sánchez Pablo^1^

^1^Hospital de Alta Especialidad del Niño Dr. Rodolfo Nieto Padrón 86030, Villahermosa, México

**Introducción**: El síndrome de Ondina es una enfermedad neurológica rara con fallo en el control automático de la respiración, que provoca hipoxia, hipercapnia y riesgo vital. Es un trastorno poco diagnosticado que podría explicar alteraciones en el desarrollo infantil y muertes súbitas. Descrito por primera vez en 1970, su incidencia estimada es de 1 por cada 200.000 nacidos vivos. **Objetivo**: Presentación de un caso de hipoventilación central congénita (síndrome de Ondina), así como sus características. **Resumen Clínico del Caso Con Resultado de Estudios, Tratamiento Instaurado y Evolución**: Antecedentes: Madre de 28 y padre de 30 años, ambos sanos; hermanos sanos. Sin antecedentes familiares neurológicos o psiquiátricos. Vacunación incompleta. Embarazo controlado y parto abdominal por postérmino y polihidramnios. Buen inicio respiratorio, somatometría adecuada y egresó a las 24 horas. Desde el nacimiento presentó cianosis durante la alimentación y dificultad respiratoria. Ingresó como posible cardiopatía, descartándose por ecografía y radiografía de tórax. Se detectó eventos de apnea y se aseguró la vía aérea. Apneas persistentes con hipercapnia (180–200 mmHg). Presentó una convulsión tónica provocada; se realiza EEG sin actividad epileptiforme. Se descartó patología del tallo cerebral (por resonancia magnética), cardiaca, respiratoria o neuromuscular. Se realizó prueba de EEG más toma de signos vitales en sueño y vigilia, se evidenció apnea durante el sueño. **Conclusiones**: El síndrome de Ondina presenta hipoventilación durante el sueño, pese a una ventilación normal en vigilia. Se asocia a mutaciones del gen *PHOX2B* (cromosoma 4p12), con herencia autosómica dominante. Este gen regula la migración de células de la cresta neural, lo que explica posibles asociaciones con otras alteraciones. Es un diagnóstico de exclusión, basado en hipoventilación del sueño (PaCO_2_ >60 mmHg), inicio temprano y ausencia de otras causas. En este caso, el paciente presenta EEG y RMN normales, y prueba genética confirmatoria con triplete anómalo en PHOX2B.

**Palabras Clave:**

síndrome de Ondina; apnea central del sueño; hipoventilación central congénita; neurología pediátrica; mutación; PHOX2B

**Hemiplejia Alternante de la Infancia: Presentación de un Caso Clínico en Paciente Pediátrica con Retraso del Neurodesarrollo**

Joel Iván Arriaga-Espinosa^1^, Araceli Reyes-Cuayahuitl^1^, Luis Antonio Arenas-Aguayo^1^

^1^Hospital de Pediatría CMN Siglo XXI, 06720 Ciudad de México, México

**Introducción**: La hemiplejía alternante de la infancia (HAI) es un trastorno neurológico raro que se manifiesta antes de los 18 meses de edad. Se caracteriza por episodios recurrentes hemiplejía y que pueden alternar de un lado al otro o involucrar ambos lados simultáneamente. La duración es de minutos hasta días y suelen resolverse durante el sueño y reaparecer al despertar. Puede estar acompañada por nistagmo, distonía, coreoatetosis y crisis epilépticas. La mayoría de los casos se asocian con mutaciones en el gen *ATP1A3*. **Objetivo**: Descripción clínica de un paciente con diagnóstico clínico de hemiplejia alternante de la infancia. **Resumen Clínico del Caso Con Resultado de Estudios, Tratamiento Instaurado y Evolución**: Femenino de 6 años con diagnóstico previo de epilepsia, con episodios caracterizados por supraversión cefálica y ocular, seguido de incremento del tono generalizado acompañado de alteración de la conciencia, con duración de 20 segundos y periodo postictal de 10 minutos, con frecuencia de uno cada dos semanas. Fue manejada con fenitoína durante un año con reducción de la frecuencia, posteriormente recibió valproato y carbamazepina sin respuesta incrementando los fenómenos 4 al día cada quince días. A los 4 años se resuelven los fenómenos descritos anteriormente, pero iniciaron episodios de hemiplejía alternante con duración de hasta 4 días acompañados de dificultad para la deglución, con duración de 10 minutos, en ocasiones desencadenan por emociones. Actualmente con persistencia del cuadro. Registro electroencefalográfico sin actividad irritativa cortical de tipo epileptiforme, estudio de imagen sin alteraciones estructurales. Se inicia manejo con flunarizina con mejoría del cuadro clínico. **Conclusiones**: La HAI es una entidad neurológica poco frecuente que puede confundirse con epilepsia refractaria, especialmente en etapas iniciales. La identificación y clasificación de los episodios, así como la ausencia de hallazgos epileptiformes en los estudios complementarios deben orientar el diagnóstico. El reconocimiento oportuno permite iniciar tratamiento específico, como la flunarizina, con el objetivo de reducir la frecuencia y duración de los episodios, mejorando así la calidad de vida y el pronóstico funcional del paciente.

**Palabras Clave:**

hemiplejía alternante; epilepsia; pediatría

**Epilepsia Genética Asociada a Mutación en el Gen *DEPDC5*: Reporte de Caso Clínico**

Brenda Irais Alvarez-González^1^, Antonio Bravo-Oro^1^, Jorge Luis Garcia-Ramírez^1^

^1^Hospital “Dr. Ignacio Morones Prieto”, 78290 San Luis Potosí, México

**Introducción**: Las mutaciones en el gen *DEPDC5* se han identificado como una causa de epilepsia focal. El gen *DEPDC5* forma parte del complejo GATOR1, codifica una proteína reguladora de la vía mTORC1. Hasta la fecha se han reportado más de 80 mutaciones, Este tipo de epilepsia se caracteriza por ser una epilepsia refractaria al tratamiento y se asocia con espasmos infantiles y en algunos casos con discapacidad intelectual y trastorno del espectro autista. **Objetivo**: Describir caso clínico de Epilepsia asociada a mutación en el gen *DEPDC5* con el objetivo de revisar la presentación clínica y correlación fenotípica, así como las características de las crisis epilépticas con esta mutación y su relación con alteraciones estructurales en IRM. **Resumen Clínico del Caso Con Resultado de Estudios, Tratamiento Instaurado y Evolución**: Se trata de paciente femenino de 3 años de edad quien acude a valoración por primera vez al mes de edad por presentar espasmos infantiles, posteriormente crisis epilépticas focales, se realizó IRM cráneo donde se reporta displasia cortical. Paciente con epilepsia de difícil control actualmente con carbamazepina y valproato de magnesio. A la exploración física, hipotonía generalizada, no ha logrado la marcha autónoma, ni lenguaje expresivo y receptivo esperado para la edad. Valorada por genética quien solicita secuenciación exómica. Paciente valorada por el servicio de neurocirugía para cirugía de epilepsia, pero por la localización de la displasia la considera no candidata a cirugía. **Resultados**: Se identificó variante patogénica c.4122C>A p. (Tyr1374*) en heterocigosis en el gen *DEPDC5* autosómica dominante. **Conclusiones**: Analizamos la relación fenotipo genotipo de epilepsia asociada a mutación en el gen *DEPDC5* y su relación con displasia cortical, destacando la importancia de su detección genética y tratamientos a utilizar en estos pacientes.

**Palabras Clave:**

epilepsia; vía mTOR; DEPDC5; displasia cortical; trastorno del neurodesarrollo

**Síndrome de Sturge Weber Tipo III en Paciente de 14 Años, a Propósito de un Caso**

Rodrigo Arispe Adriana Blanca^1^, Castañeda Loeza Selene^1^, Roldán Gutiérrez Martin Iván^1^, Rodríguez García Verónica^1^, Venta Sobero José Antonio^1^

^1^Centro Médico Nacional 20 de Noviembre, ISSSTE, 03104 Ciudad de México, México

**Introducción**: El Síndrome de Sturge-Weber (SSW) es una enfermedad neurocutánea rara que se subdivide en 3 tipos, siendo el tipo III el más raro representando un 10% de los casos de SSW descritos en la literatura; su presentación en edad pediátrica es muy rara. Este tipo se caracteriza por afectación neurológica aislada, siendo los síntomas más frecuentes crisis convulsivas febriles complejas y/o cefalea de tipo migrañosa, sin malformaciones cutáneas ni oculares, lo que dificulta su diagnóstico. **Objetivo**: Describir la presentación clínica y hallazgos imagenológicos en un caso de SSW tipo III en paciente pediátrico. **Resumen Clínico del Caso Con Resultado de Estudios, Tratamiento Instaurado y Evolución**: Femenino adolescente con antecedentes de crisis epilépticas focales desde lactante y discapacidad intelectual desde etapa escolar. Se mantuvo con monoterapia libre de crisis durante 11 años. A los 14 años de edad inicia con cefalea de tipo migrañoso y reinicia con episodio de crisis epilépticas focales por suspensión del fármaco anticrisis. En TAC de cráneo se observa hemangioma calcificado; en estudio de RMN de cerebro se encuentra atrofia hemisférica izquierda, angioma pial hemisférico derecho y reforzamiento leptomeníngeo a nivel temporoparietooccipital derecho y temporooccipital izquierdo con calcificaciones cerebrales a este nivel. Con este reporte se estableció el diagnóstico de SSW tipo III al no encontrarse presencia de hemangioma facial o alteraciones oculares. Se continuó con monoterapia anticrisis y se mantendrá vigilancia del angioma pial. **Conclusiones**: Su evolución ha sido favorable, con disminución de los eventos de cefalea con tratamiento abortivo y con control clínico de crisis epilépticas con monoterapia.

**Palabras Clave:**

síndrome de Sturge Weber tipo III; cefalea; crisis epilépticas; resonancia magnética cerebral

**Eritromelalgia Primaria Reporte de Caso Clínico**

Burgos Navarro Alan^1^, Armejo Chávez Luz Elena^1^, Hernández Villaseñor Enrique^1^

^1^Unidad Médica de Alta Especialidad Hospital Pediátrico, Centro Médico Nacional de Occidente, 44340 Guadalajara, México

**Introducción**: La eritromelalgia, también conocida como síndrome de los pies ardientes, es una enfermedad rara, crónica. Caracterizada por la triada de eritema, hipertermia y dolor urente de la región afectada, predominantemente los miembros pélvicos. Se clasifica en eritromelalgia primaria (EP), la cual es de etiología idiopática, debido a una neuropatía autosómica caracterizada por la mutación de los genes *SCN9A*, *SCN10A* y *SCN11A* que se encargan de codificar la subunidad alfa de los canales de sodio dependientes de voltaje. Estos canales se expresan en los ganglios de la raíz dorsal de las neuronas ganglionares simpáticas. Esta mutación conduce a la hiperexcitabilidad de las fibras nociceptivas, causando su activación con estímulos subumbrales, en una respuesta dolorosa ante estímulos no dolorosos. **Objetivo**: Describir caso clínico de paciente con Eritromelalgia Primaria. **Resumen Clínico del Caso Con Resultado de Estudios, Tratamiento Instaurado y Evolución**: Paciente femenino de 15 años curso con cuadro de dolor crónico durante 14 años. Inicia con dolor a el año de edad, con presencia de dolor urente constaten con escala de EVA 10/10 incapacitante sin lograr el sueño de manera adecuada. En tratamiento crónico con AINES y opiáceos, Dicho dolor presentaba exacerbación con el calor, además de presentar escoliosis álgica dentro del cuadro clínico con eritema el cual al principio del cuadro era limitado a pie sin embargo actualmente abarcaba por enzima del tobillo, dicho cuadro se trato por parte de psiquiatría sin mejoría del cuadro además valorada por dermatología en el 2023 tratada como dermatitis atópica, Se cataloga como Neuropatía de fibras pequeñas probable Eritromelalgia ante la presencia de triada de dicha patología al no contar con presupuesto para panel genético se realiza prueba terapéutica con Carbamazepina con mejoría clínica. Actualmente paciente realiza sus actividades diarias sin complicaciones. **Conclusiones**: Durante el abordaje multidisciplinario. Se lograron descartar causas secundarias sin embargo el diagnóstico de la Eritromelalgia es genético por la afectación en los genes *SCN9A*, *SCN10A* y *SCN11A*. A pesar de estar en una unidad de tercer nivel no contamos con dichos estudios por lo que se realiza prueba terapéutica con Carbamazepina con mejoría sintomática.

**Palabras Clave:**

eritromelalgia; dolor crónico; triada; eritema; hipertermia y dolor urente de la región afectada; canales de sodio

**Neuroinfección por Herpes Humano Tipo 6**

Marco Antonio Vidal-Peregrina^1^, Fabiola Marycruz De la Fuente-Silva^1^, Marisol Burgoin-Rivero^1^, Martín Arturo Silva-Ramírez^1^, Flora Cebada-López^1^

^1^Instituto Mexicano Del Seguro Social, UMAE Hospital General “Dr. Gaudencio González Garza” Centro Médico Nacional La Raza, 02990 Ciudad de México, México

**Introducción**: El Virus del Herpes Humano tipo 6 (HHV-6) es una de las principales causas de enfermedades febriles agudas en Pediatría, su asociación con neuroinfección a nivel mundial es del 1–2%. **Objetivo**: Presentación de dos casos de neuroinfección por HHV-6. **Resumen Clínico del Caso Con Resultado de Estudios, Tratamiento Instaurado y Evolución**: Caso 1: Femenino de 11 meses, con retraso de neurodesarrollo. Inicia con fiebre de hasta 40°C, diarrea, presenta dermatosis maculopapular generalizada, valorada en Urgencias con postura de opistótonos, pupilas de 2 mm, movimientos clónicos extremidad superior derecha, signos meníngeos, se administra benzodiazepina IV y Fenitoína. TC de cráneo edema cerebral moderado, reforzamiento parietotemporal bilateral. Tratamiento con Aciclovir, punción lumbar sin alteraciones, FilmArray HHV-6 positivo, cambio a Ganciclovir 14 días, amerita intubación e ingreso a UTIP con persistencia de postura en opistótonos, crisis focales clónicas extremidad superior derecha, se agrega Levetiracetam. Durante estancia en Infectología Pediátrica sin crisis epilépticas, RM de cráneo normal, egresa con Valproato de magnesio. Seguimiento en Consulta Externa con alteración de ciclo sueño-vigilia, sin sostén cefálico, distonías, crisis epilépticas, EEG sin grafoelementos de sueño. Caso 2: Masculino de 11 meses previamente sano. Inicia con rinorrea, tos, posteriormente disnea acudiendo a hospital particular con deshidratación, se detecta hiponatremia, alteración del estado de alerta, chupeteo, aumento del tono global, tratamiento con Fenitoína, envío a HGZ donde se sospecha en Encefalitis, punción lumbar con células de 16, FilmArray HHV-6 positivo, enviado a Hospital de Infectología. A su ingreso se detecta parálisis de VI nervio craneal bilateral y dermatosis maculopapular en tronco y abdomen, tratado con Ganciclovir 14 días y Dexametasona. TC cráneo con atrofia temporal bilateral, EEG con múltiples artefactos, egresa libre de crisis epilépticas. **Conclusiones**: El HHV-6 es un virus neurotrópico, puede permanecer latente en sistema nervioso central, se ha relacionado con crisis febriles, esclerosis múltiple, esclerosis mesial temporal, epilepsia, romboencefalitis y neuroinfección. No existen antivirales aprobados sin embargo se recomienda el uso de Ganciclovir.

**Palabras Clave:**

Herpes-6; neuroinfección; ganciclovir; pediatría

**Perfil Epiléptico e Imagenológico de una Serie de Pacientes con Leucodistrofia Metacromática en el Instituto Nacional de Pediatría**

Mayorga Chávez Gabriela Carolina^1^, Herrera Mora Patricia^1^

^1^Instituto Nacional de Pediatría, 04530 Ciudad de México, México

**Introducción**: La leucodistrofia metacromática es una enfermedad autosómica recesiva causada por deficiencia de arilsulfatasa A, que produce acumulación de sulfátidos y desmielinización progresiva del sistema nervioso central y periférico. Se manifiesta clínicamente con deterioro motor, cognitivo y, en algunos casos, epilepsia refractaria. **Objetivo**: Describir el perfil epiléptico e imagenológico, de 4 pacientes con leucodistrofia metacromática. **Resumen Clínico del Caso Con Resultado de Estudios, Tratamiento Instaurado y Evolución**: Material y Métodos Serie de casos retrospectiva de cuatro pacientes con diagnóstico confirmado de leucodistrofia metacromática por estudio enzimático y/o genético, atendidos entre 2011 y 2023. Se analizaron variables clínicas, tipo y frecuencia de crisis epilépticas, tratamientos farmacológicos, hallazgos por resonancia magnética cerebral, electroencefalogramas. **Resultados**: Tres de los cuatro pacientes presentaron epilepsia, siendo focal refractaria en dos de ellos, los tres pacientes con crisis de inicio focal con evolución a bilateral, en cuanto a la frecuencia, un paciente presentó 8 crisis por semana, otro 2 crisis mensuales, y un tercero se encontraba libre de crisis un año. El tratamiento incluyó múltiples fármacos como levetiracetam, oxcarbazepina, lacosamida, clonazepam y topiramato. El EEG evidenció actividad epileptiforme multifocal y lentificación generalizada en los 3 pacientes con epilepsia. Todos los pacientes mostraron hallazgos imagenológicos compatibles con leucodistrofia. Las resonancias magnéticas revelaron ventriculomegalia supratentorial en los cuatro casos, con hipomielinización periventricular bilateral. Dos pacientes presentaron adelgazamiento o hipoplasia del cuerpo calloso, mientras que uno mostró además desmielinización en centros semiovales, tálamos y cerebelo. Estos hallazgos son consistentes con el patrón de desmielinización progresiva observado en la leucodistrofia metacromática. Un paciente falleció a los 7 años; los demás mostraron deterioro progresivo dos de ellos en estado de mínima conciencia y uno en estado vegetativo. **Conclusiones**: La epilepsia focal refractaria es una manifestación común en leucodistrofia metacromática. Los electroencefalogramas mostraron actividad epileptiforme y lentificación generalizada, en correlación con los hallazgos por resonancia magnética. La evaluación clínica, imagenológica y electrofisiológica integrada es clave para el diagnóstico y abordaje oportuno.

**Palabras Clave:**

leucodistrofia metacromática; arilsulfatasa A; epilepsia refractaria; desmielinización; neuroimagen; encefalopatía

**Sospecha de Deficiencia de 3 Metilcrotonil – Coa Carboxilasa Como Causa de Epilepsia Metabólica: Reporte de Caso**

Diana Laura Gutiérrez Ramírez^1^, Alfredo Falcón Delgado^1^, Adriana C. Cantú Salinas^1^, Ana L. Carreón Garcia^1^, Beatriz Eugenia Chavez Luevanos^1^

^1^Servicio de Neurología, Hospital Universitario “Dr. José Eleuterio González” Universidad Autónoma de Nuevo León, 64460 Monterrey, México

**Introducción**: Se han identificado alrededor de 600 epilepsias metabólicas, reconocer las crisis como posibles síntomas de enfermedades metabólicas, permitirá una sospecha diagnóstica temprana, orientando abordaje diagnóstico para ofrecer un tratamiento específico. La deficiencia de 3 – metilcrotonil – CoA carboxilasa (MCC) produce la excreción elevada de 3 – metilcrotonilglicina (3 – MCG) y ácido 3 – hidroxiisovalérico (3 – HIVA), con un fenotipo clínico variable. **Objetivo**: Describir características clínicas de paciente con laboratoriales coincidentes con sospecha de MCC. **Resumen Clínico del Caso Con Resultado de Estudios, Tratamiento Instaurado y Evolución**: Masculino, 9 años, mexicano, hermana con trastorno cognitivo no específico, institucionalizado en casa hogar (2023), diagnósticos de ingreso: peso y talla bajos para la edad, dismorfias faciales, sospecha de discapacidad intelectual, con hiporexia frecuente, insomnio y trastorno de conducta. Primera crisis en 2024 focal, con arresto del comportamiento, sialorrea y pérdida de control de esfínteres, se inicia valproato de magnesio 15 mgkgdia, electroencefalograma normal, continúa con crisis de desconexión, se realiza resonancia magnética (RM) simple de encéfalo, lesiones hiperintensas de pequeño tamaño infra y supratentoriales subcorticales frontales y occipitales bilaterales, en pedúnculo mesencefálico izquierdo y periventriculares sugestivas de gliosis vs desmielinización. Noviembre del 2024 se agrega temblor de reposo en miembros superiores y mioclonías intermitentes de miembro superior izquierdo. Se aborda para búsqueda de metabolopatía, se realiza perfil de ácidos orgánicos en orina, de aminoácidos y acilcarnitinas, donde se reporta niveles urinarios elevados de ácido 3 – hidroxiisovalérico (3 – HIVA) y 3 – metilcrotonilglicina (3 – MCG). con 3-hidroxiisovalerilcarnitina (C_4_OH) presente en sangre y orina. Se realiza sospecha de MCC ante hallazgos bioquímicos, debido a no contar con posibilidad de realización de estudio genético, se decide inicio de dieta baja en leucina y alta en carbohidratos, con continuidad de tratamiento anticrisis, con lo que se logra libertad de crisis al momento por 3 meses, disminución de anticrisis y resolución de trastorno del movimiento. **Conclusiones**: La epilepsia de etiología metabólica, no cuenta con un cuadro clínico o características en paraclínicos específicos, requiere un alto nivel de sospecha para su diagnóstico.

**Palabras Clave:**

epilepsia; metabólico; ácidos orgánicos

**Variabilidad Clínica de la Atrofia Muscular Espinal Tipo 2. Serie de Casos**

Castro Ruiz-Alfredo^1^, Gutiérrez Soto-Marisol^1^, Reyes Cuayahuitl-Araceli^1^, Rayo Mares-Jesús Darío^1^

^1^Servicio de Neurología Pediátrica, Hospital de Pediatría CMN Siglo XXI, IMSS, 06720 Ciudad de México, México

**Introducción**: La Atrofia Muscular Espinal (AME) es un trastorno autosómico recesivo, caracterizado por la degeneración de las neuronas motoras inferiores en la médula espinal. Las manifestaciones clínicas son hipotonía, debilidad, fasciculaciones, REMs disminuidos o ausentes. Se clasifica en diferentes tipos, siendo el tipo 1 (60%) y tipo 2 (20%) las formas más frecuentes. Ambas están causadas por mutaciones en el gen *SMN* (cr5q13), el fenotipo depende del número de copias del gen *SMN2*. **Objetivo**: Describir 4 casos clínicos de pacientes con AME tipo 2. **Resumen Clínico del Caso Con Resultado de Estudios, Tratamiento Instaurado y Evolución**: Caso1: Femenino de 3 años, con hipotonía periférica, fasciculaciones y arreflexia. Con estudio molecular evidenciando SMN1:0 copias SMN2: (AME 2B). Obtiene 7 puntos de la escala HFMSE Actualmente en tratamiento con Risdiplam, seis meses posteriores al tratamiento HFMSE 25 puntos. Caso clínico 2: Femenino de 3 años 6 meses, inicia con debilidad y arreflexia a los 8 meses, análisis molecular con 0 copias de SMN1, con 3 copias de SMN2. Actualmente con escoliosis no restrictiva y escala HFMSE 25 puntos. Sin tratamiento modificador. Caso clínico 3: Masculino de 15 año, inicia a los dos años con caídas frecuentes y arreflexia, pérdida de la bipedestación y sedestación independiente, estudio molecular ausencia de exones 7 y 8 (SMN1) y 3 copias del SMN2. Escala HFMSE 18 puntos. Sin tratamiento modificador. Caso clínico 4: Masculino de 10 años, inicia a los 10 meses al no lograr la bipedestación, a los 2 años de sin ganancias motoras, hipotonía, debilidad y arreflexia, análisis molecular SMN1 0 copias y 3 copias de SMN2. Actualmente en silla de ruedas, con diagnóstico de apnea obstructiva del sueño. Escala HFMSE 10 puntos. Sin tratamiento modificador. **Conclusiones**: La AME es una condición que debe ser sospechada ante un paciente con debilidad, arreflexia y fasciculaciones, como en los casos presentados, es sabido que la cantidad de copias del gen *SMN2* está relacionado con la gravedad de la enfermedad, la evolución es variable, sin embargo, con el advenimiento de tratamientos modificadores de la enfermedad la sobrevida ha aumentado, así como, las ganancias motoras, evidenciado en literatura actual y en el caso número 1.

**Palabras Clave:**

atrofia muscular espinal; SMN1; SMN2; hipotonía; arreflexia; fasciculaciones

**Síndrome de Guillain-Barré Recurrente en Pediatría. Reporte de Caso**

María Fernanda Pérez-Álvarez^1^, Fabiola Marycruz De la Fuente-Silva^1^, Salvador Jiménez-Mejinez^2^, Daniela Méndez-Fernández^1^, Marco Antonio Vidal-Peregrina^1^, Alberto Ibarra-Delgado^1^, Katia Moreno-Silva^1^, Martín Arturo Silva-Ramírez^1^, Flora Cebada-López^1^

^1^Servicio de Neurología, UMAE Hospital General “Dr. Gaudencio González Garza”, Centro Médico Nacional La Raza, IMSS, 02990 Ciudad de México, México 

^2^Hospital General de Zona No. 9, Instituto Mexicano del Seguro Social ,IMSS, 49000 Ciudad Guzmán, México

**Introducción**: El Síndrome de Guillain-Barré (SGB) es una polineuropatía inmunomediada de inicio agudo que se caracteriza por debilidad muscular simétrica ascendente. Se estima que el 2–5% puede presentar un secundo cuadro catalogándose como recurrente. Se define con un intervalo mínimo de ≥4 meses entre los episodios si el paciente no se recuperó por completo (Hughes ≥2) o ≥2 meses cuando se recuperó por completo o casi por completo (Hughes ≤1) después de 1 episodio. El diagnóstico y tratamiento no difiere del primer evento de Guillain barre. **Objetivo**: Presentación de caso de SGB recurrente en Pediatría. **Resumen Clínico del Caso Con Resultado de Estudios, Tratamiento Instaurado y Evolución**: Femenino de 4 años quien cursó con SGB Hughes 5 variante neuropatía axonal motora-sensitiva aguda (AMSAN), tratada con inmunoglobulina humana, recuperando la marcha a los 4 meses de su egreso hospitalario. A los 11 años presenta cuadro de debilidad muscular en extremidades inferiores, disfagia, sin lograr bipedestación, acompañado de arreflexia, ausencia reflejo nauseoso y tusígeno, con antecedente de gastroenteritis 2 días previos al inicio de síntomas neurológicos. Se realiza punción lumbar repostando disociación albumino-citológica, dados los antecedentes se cataloga como Síndrome de Guillain-Barré recurrente Hughes 5. Velocidades de conducción nerviosa reportan variable AMSAN e hisopado rectal con Campylobacter jejuni, manejada con inmunoglobulina a 2 grs/kg. Se egresa a domicilio con Hughes 5, portadora de traqueostomía y gastrostomía. La paciente logra sedestación a los 2 meses de su egreso, con deambulación a los 4 meses con ayuda. Actualmente (8 meses del cuadro) logra deambulación independiente, con recuperación clínica completa. **Conclusiones**: El síndrome de Guillain barre es una entidad subnotificada, es necesario indagar sobre antecedente de evento previo de Guillain barre, así mismo, de sus características en cuanto evolución, dado que su principal diferencial es una polineuropatía desmielinizante inflamatoria crónica (CIDP). Actualmente muchos autores continúan en controversia sobre dicho padecimiento, se requiere de mayor difusión y reporte de casos para catalogar sus principales características.

**Palabras Clave:**

Guillain-Barre recurrente; polineuropatía inflamatoria

**Desafío Diagnóstico de Síndrome Miopático Congénito Infrecuente en Paciente con Hipotonía: Reporte de Caso**

Narváez González Diana Elisa^1^, González González Raúl Humberto^1^, Durón Tábora Iliana^1^, Romero Díaz Viktor Javier^1^, Cantú Salinas Adriana Carlota^1^, Carrión García Ana Luisa^1^, Vázquez Fuentes Salvador^1^, De la Garza Pineda Oscar^1^, Beatriz E. Chávez-Luevanos^1^

^1^Servicio de Neurología Pediátrica, Hospital Universitario “Dr. José Eleuterio González”, Universidad Autónoma de Nuevo León, 64460 Monterrey, México

**Introducción**: El diagnóstico de precisión etiológica en el recién nacido con hipotonía nos orienta hacia el patrón evolutivo, comorbilidades y opciones terapéuticas. Entre ellas, la miopatía nemalínica es un trastorno neuromuscular donde existe la mutación de los genes que codifican proteínas estructurales de filamentos delgados formados por la hélice polimerizada de α-actina codificada por *ACTA-1*, llamados cuerpos nemalínicos “bastones” en la fibra muscular. **Objetivo**: Describir el abordaje y diagnóstico de miopatía nemalínica en recién nacido con hipotonía. **Resumen Clínico del Caso Con Resultado de Estudios, Tratamiento Instaurado y Evolución**: Recién nacido a término, sin antecedentes familiares, producto de cuarta gestación, embarazo normoevolutivo, adecuado peso y talla, APGAR 8/8, Silverman Anderson 2 puntos, con necesidad de ventilación mecánica invasiva a las 12 horas de vida. Trasladada a nuestra unidad por probable encefalopatía hipóxico-isquémica. Al examen neurológico con hipotonía axial y debilidad proximal, fuerza 1/5 en escala de Daniels, ausencia de reflejos primitivos y de estiramiento muscular; CPK de 24,6 UI/L, ecografía transfontanelar y resonancia magnética cerebral normales, registro de electroencefalograma de 24 horas con actividad cortical discontinua generalizada sin evidencia de crisis epilépticas; Tamiz metabólico ampliado normal. Se realiza prueba con neostigmina y estudio electrofisiológico no concluyente, por lo que se solicita biopsia muscular en la cual se reporta presencia de fibras musculares y alteraciones estructurales asociadas a la formación de bastones y de la proteína alfa-actinina , confirmando diagnóstico de miopatía nemalínica tipo ACTA 1: c.758G>A (p. Gly253Asp) por medio de panel genético de enfermedades neuromusculares. **Conclusiones**: El conocer nuevas etiologías permite ampliar las sospechas diagnósticas, para un abordaje neurológico y multidisciplinario oportuno, buscando mejorar la calidad de vida del paciente.

**Palabras Clave:**

miopatía; nemalina; congénito; hipotonía; biopsia

**Síndrome de Joubert: Reporte de Caso**

Vázquez Villarreal Karolina Lizeth^1^, Calderón Sepúlveda Raúl Calderón^1^, Ancer Ochoa Jorge Abraham^1^

^1^Programa Multicéntrico de Especialidades, Tecnológico de Monterrey, 64849 Monterrey, México

**Introducción**: El síndrome de Joubert (SJ) es una ataxia cerebelosa congénita con herencia autosómica recesiva o ligada al cromosoma X, caracterizado por hipotonía, ataxia, movimientos oculares anormales y discapacidad intelectual. Es causado por mutaciones recesivas encontrados en más de 27 genes, todos los cuales codifican proteínas que se localizan en el cilio primario, la disfunción de este orgánulo conduce a una serie de enfermedades conocidas como “ciliopatías”; estos trastornos tienen una variedad de fenotipos que incluye disfunción cognitiva, malformaciones del sistema nervioso central, enfermedad renal, anomalías esqueléticas y craneofaciales. El SJ presenta una malformación característica del cerebelo y el tronco encefálico, reconocible en la resonancia magnética cerebral axial “signo del diente molar” que comprende: una fosa interpeduncular profunda, pedúnculos cerebelosos prominentes, hipoplasia del vermis. Tiene una incidencia entre 1/80.000 y 1/100.000 nacidos vivos. **Objetivo**: Presentar el caso clínico de una paciente con Síndrome de Joubert, a la que se le atribuyeron los datos clínicos presentados en la infancia a evento de asfixia perinatal. **Resumen Clínico del Caso Con Resultado de Estudios, Tratamiento Instaurado y Evolución**: Antecedentes: Retraso psicomotor diagnosticado a los 6 meses. Cirugía de estrabismo a los 3 años. Traumatismo craneoencefálico leve a los 5 años. Cesárea por pérdida de bienestar fetal, asfixia perinatal. Paciente femenina de 9 años con episodios de movimientos anormales en extremidades y desviación de la comisura bucal. Los episodios duran 15 minutos, seguidos de somnolencia de 30 minutos, con resolución espontánea, presentados 2 ocasiones. Examen físico: rasgos faciales dismórficos (orejas bajas, frente ancha), peso > percentil 99, estrabismo, ataxia y disminución de fuerza en extremidades derechas. TAC de encéfalo muestra hipoplasia cerebelosa, ausencia del vermis, elongación de pedúnculos cerebelosos y “signo del diente molar”. Diagnóstico: síndrome de Joubert, basado en hallazgos clínicos y de neuroimagen. **Conclusiones**: Paciente con hipotonía desde los 6 meses y disfunción oculomotora, inicialmente atribuida a asfixia perinatal. A los 9 años, presentó un evento neurológico y en la neuroimagen se evidenció el signo del diente molar. El síndrome de Joubert es un trastorno del neurodesarrollo con manifestaciones clínicas y genéticas variables, cuyo diagnóstico requiere historia clínica y estudios de imagen.

**Palabras Clave:**

Joubert; diente molar; ciliopatía

**Epilepsia Genética por Variante Patogénica en el Gen *NPRL3*: Asociación con Pubertad Precoz e Hipotiroidismo**

Pérez Rodríguez Juan Steven^1^, José de Jesús Vázquez Montante^1^, García Ramírez Jorge Luis^1^

^1^HRAE Dr. Ignacio Morones Prieto, 78290 San Luis Potosí, México

**Introducción**: Antecedentes: Variantes patogénicas en el gen NPRL3 se asocian a epilepsia de inicio temprano en los primeros 6 meses de vida, hipotonía y retraso del neurodesarrollo. Las crisis convulsivas comúnmente suelen ser complejas e incluyen espasmos epilépticos, crisis mioclónicas, tónicas y tónico-clónicas. Al principio, el EEG puede no revelar anomalías epileptiformes significativas; sin embargo, con el tiempo, se evidencian descargas epileptiformes abundantes y multifocales. Aunado a esto se encuentra relacionado con alteraciones del sistema endocrino como pubertad precoz. No sé cuenta con tratamiento específico por lo cual requiere tratamiento multidisciplinario incluyendo terapia anticrisis, dieta ecogénica, fisioterapia y rehabilitación. **Objetivo**: Discusión de las características clínicas y variante patogénica de un paciente en el Hospital regional de alta especialidad “Ignacio Morones Prieto”. **Resumen Clínico del Caso Con Resultado de Estudios, Tratamiento Instaurado y Evolución**: Masculino de 12 años de edad cuenta con diagnóstico de epilepsia focal motora genética por mutación NPRL3 de 9 años de edad. Resonancia magnética de cráneo normal. Primera crisis a los 8 años de edad, focales de tipo mioclónico sin generalización secundaria, no se logró control de las crisis requiriendo multitratamiento logrando control con Vigabatrina, Levetiracetam y Valproato de Magnesio, logrando control Casi total de las crisis. Presentando descontrol esporádico con aplicación de Análogos de GNRH lo que requiere reajuste terapéutico. **Conclusiones**: Mutaciones en NPRL3 son causa de epilepsias de difícil control requiriendo en muchas ocasiones un manejo multidisciplinario para mejorar la calidad de vida de los pacientes además de lograr la mayor funcionalidad e independencia posible. En cuanto a las comorbilidades que presenta nuestro paciente lo coloca en una condición de vulnerabilidad respecto al control adecuado de crisis permitiendo además lograr una talla y desarrollo adecuado respecto a su pubertad precoz, lo cual nos plantea una necesidad mayor en la comprensión de la fisiología hormonal en SNC además de su relación con los distintos de tipos de mecanismo que incrementan o disminuyen los umbrales para presentar crisis.

**Palabras Clave:**

epilepsia; pubertad precoz; NPRL3

**Distonía de Origen Genético Evolución y Tratamiento**

Reyes Cuayahuitl-Araceli^1^, Rayo Mares-Jesús Darío^1^, Cárdenas Conejo-Alan^1^, Gutiérrez Soto-Marisol^1^, López Gallegos-Gabriela Monserrat^1^

^1^Hospital de Pediatría CMN Siglo XXI, IMSS, 06720, Ciudad de México, México

**Introducción**: La distonía es un trastorno del movimiento caracterizado por contracciones musculares sostenidas o intermitentes estereotipados. Se han reportado causas genéticas y no genéticas. Se han identificado más de 200 genes como causa de distonía generalizada de inicio en la infancia. Dentro de las formas monogénicas de las distonías se encuentran las distonías aisladas dentro de las cuales están *TOR1A* y *KMT2B* y dentro de las condiciones que se asocian a alteración en el neurodesarrollo asociado a distonía esta *CTNNB1*. **Objetivo**: Describir la presentación clínica y tratamiento de tres pacientes con Mutación en *CTNNB1*, *TOR1A* y *KMT2B* relacionados con distonía. **Resumen Clínico del Caso Con Resultado de Estudios, Tratamiento Instaurado y Evolución**: Caso clínico 1: Masculino de 13 años con retraso en el neurodesarrollo. A los 12 meses movimientos distónicos generalizados. A los 3 años crisis febriles TCG. EEG con puntas frontotemporales bilaterales. RNM de encéfalo con atrofia. Variante patogénica gen *CTNNB1*. En tratamiento con valproato, baclofeno y toxina botulínica con buena respuesta. Caso clínico 2: Femenino de 10 años. Prematurez. A los 5 años inicia con alteración en la marcha y cefalea. A los 7 años debilidad de extremidades superiores, cervical y en los músculos de la masticación. Variante patogénica en *KMT2B*. RNM sin alteraciones. A la exploración con movimientos distónicos de extremidades superiores, cervical y orofacial, braquidactilia tipo D. Tratamiento con levodopa / carbidopa y toxina botulínica con buena respuesta. Caso clínico 3: Masculino de 13 años. Inicia a los 6 años con dolor en planta izquierda impidiendo deambulación. Debilidad en extremidades inferiores, movimientos incoordinados y aumento de tono. Movimientos distónicos en extremidades superiores. Mutación de *TOR1A*. Tratamiento estimulador cerebral profundo y toxina botulínica. **Conclusiones**: Las distonías genéticas en la infancia constituyen un grupo clínicamente y genéticamente heterogéneo de trastornos del movimiento, cuyo reconocimiento precoz es crucial para un manejo adecuado. La identificación de variantes genéticas patogénicas como es el caso de estos 3 pacientes permite no solo establecer un diagnóstico definitivo, sino también orientar decisiones terapéuticas, anticipar la evolución clínica y ofrecer asesoramiento genético preciso. La toxina botulínica ha resultado ser un tratamiento alentador en estos casos.

**Palabras Clave:**

trastornos del movimiento; distonía; TOR1A; CTNNB1; KMT2B

**Mielitis Transversa Longitudinalmente Extensa, Similares Presentaciones, Diferentes Etiologías**

Tejeda Guzmán Zuleyki Eugenia^1^, Cornelio Nieto José Ovidio^1^, González Medina Mario^1^

^1^Hospital Regional de Alta Especialidad del Niño Dr. Rodolfo Nieto Padrón, 86030, Villahermosa, México

**Introducción**: La mielitis transversa longitudinal extensa (MTLE) es una lesión en la médula espinal que afecta tres o más segmentos medulares y se caracteriza por síntomas como paraparesia, paraplejía, pérdida sensorial, incontinencia urinaria y anal. Las causas de MTLE pueden ser autoinmunes, metabólicos, tóxicos, inflamatorios, infecciosos, vasculares y post-vacunales. Este artículo presenta tres series de casos clínicos de MTLE, donde la presentación clínica y la etiológica fueron diferentes. **Objetivo**: Mostrar la etiología, tratamiento y evolución de tres pacientes con Mielitis Transversa Longitudinalmente extensa, tratadas en el Hospital del Niño Dr. Rodolfo Nieto Padrón. **Resumen Clínico del Caso Con Resultado de Estudios, Tratamiento Instaurado y Evolución**: Se presentan 3 series de casos de MTLE, el 100% de los síntomas iniciaron las primeras 24 hrs, 66% sexo femenino, dolor torácico 33%, dolor lumbar 33%, dolor escapular 33%, paraparesia 100%, parestesia 66%, hiporreflexia 66%, arreflexia 33%, incontinencia urinaria y anal 100%, disautonomía 33%, proteinorraquia 66%, el 100% recibió tratamiento con IGV, metilprednisolona y prednisona, 33% plasmaféresis y el 66% rituximab. El diagnostico se confirmó por RM de encéfalo y neuroeje 100% con afectación de más de 3 cuerpos medulares, causado por post vacuna de sarampión 33%, vascular 33% por anticuerpos anti MGO y Antiacuaporina-4 33%. Secuelas de hemiparesia 66%, paraparesia 33%, vesicostomia 33%, depresión 66%. **Conclusiones**: La presentación clínica fue aguda y progresiva, el diagnóstico y tratamiento oportuno fueron esenciales para la evolución. Se enfatiza la importancia de la evaluación neurológica diaria. La MTLE, al ser una lesión inflamatoria medular, puede ser la forma de presentación de un complejo de neuromielitis óptica, el cual fue diagnosticada en el caso 3, lo que resalta la necesidad de diagnóstico temprano y tratamiento oportuno, resaltando que fueron todos de etiologías diferente, donde la de etiología autoinmune tuvo mayor secuela.

**Palabras Clave:**

mielitis transversa; neuromielitis óptica; desmielinización

**Distonia y Parálisis Bulbar Asociados a Alteración del Gen *NOTCH3* en un Paciente Pediátrico, Reporte de Caso**

Ibarra Delgado Alberto^1^, Loman Zúñiga Verónica^1^, Caballero Navarro Yael^1^, Cebada López Flora^1^

^1^UMAE Hospital General, “Dr. Gaudencio González Garza”, 02990 Ciudad de México, México

**Introducción**: El síndrome de CADASIL (Cerebral Autosomal Dominant Arteriopathy with Subcortical Infarcts and Leukoencephalopathy) es una enfermedad autosómica dominante por una mutación del gen *NOTCH3*, lo cual produce una vasculitis de pequeños vasos y se manifiesta con episodios de cefalea, demencia, eventos isquémicos cerebrales, trastornos neuropsiquiátricos y en algunos casos trastornos del movimiento. **Objetivo**: Describir el espectro clínico de un paciente pediátrico con manifestaciones clínicas infrecuentes del síndrome de CADASIL, para un reconocimiento temprano y tratamiento oportuno. **Resumen Clínico del Caso Con Resultado de Estudios, Tratamiento Instaurado y Evolución**: Se presenta el caso de un paciente femenino de 12 años con inicio de padecimiento a los 9 años (2021) con manifestaciones psiquiátricas preocupación constante excesiva, bruxismo, inatención, poca a tolerancia a la frustración y mayor dificultad para tomar decisiones manejándose como trastorno de ansiedad generalizada. En noviembre 2022 presentó movimientos involuntarios con movimientos oculógiros duración de menos de 5 segundos, sin alteración del estado de conciencia. En mayo 2023 presentó eventos de cefalea holocraneana, exacerbación de movimiento oculógiros y distonías cefálicas con hipertensión de cuello con duración de 30 minutos a 1 hora los cuales mejoraban de forma espontánea con el reposo y aumentaban durante periodos de estrés en un inicio 3 veces por semana y posteriormente diario, En 2024 se agrega dificultad para el paso de los alimentos, disfagia a sólidos, así como dificultad disartria. Cuenta con electroencefalograma sin actividad paroxística y resonancia magnética de encéfalo con atrofia fronto-parietal, así como microadenoma hipofisiario. Se realizó exoma el cual reportó una variante patogénica, en estado heterocigoto en el gen *NOTCH3* compatible para síndrome de CADASIL. **Conclusiones**: Se presenta el caso de una paciente pediátrica con manifestaciones clínicas infrecuentes de síndrome de CADASIL (disfunción bulbar, síndrome regresivo cognitivo y distonía), no reportados en literatura que asociamos a manifestaciones raras en edades pediátrica. Recalcamos importancia de reconocimiento temprano de la enfermedad y abordaje oportuno debido al riesgo de eventos isquémicos a nivel cerebral, actualmente la paciente se encuentra con manejo farmacológico para distonía, así como terapia con antiagregante plaquetario.

**Palabras Clave:**

distonía; CADASIL; cefalea

**Tuberculosis Meníngea en Paciente Pediátrico: Reporte de Caso y Revisión Diagnóstica Oportuna**

Barbosa Martínez Samantha del Carmen^1^, Beltrán Nuño José Ignacio^1^

^1^Hospital para El niño IMIEM, 50170 Toluca de Lerdo, México

**Introducción**: La tuberculosis (TB) continúa siendo un problema de salud pública en México. En 2021 se reportaron 1832 casos en menores de 20 años, representando el 9% del total nacional. La tuberculosis meníngea (TBM), considerada la forma más grave de TB, constituye el 4,7% de los casos pediátricos. Su diagnóstico suele retrasarse debido a la inespecificidad del cuadro clínico inicial, lo que incrementa el riesgo de secuelas neurológicas y mortalidad. **Objetivo**: Presentar un caso de TBM en paciente pediátrico, enfatizar la importancia del reconocimiento clínico temprano y el uso de herramientas diagnósticas específicas para mejorar el pronóstico. **Resumen Clínico del Caso Con Resultado de Estudios, Tratamiento Instaurado y Evolución**: Se describe el caso de un paciente masculino de 4 años, previamente sano, con esquema de vacunación completo, incluido BCG, antecedentes familiares de TB negados. Presentó traumatismo craneoencefálico leve el 22/10/2024. Días después desarrolla fiebre de 38,8 °C, cefalea intermitente e intolerancia a la vía oral. Evoluciona con irritabilidad y alteraciones conductuales, se realiza TAC de cráneo el 04/11/2024, sin hallazgos relevantes. El 11/11/2024, se identifica anisocoria, parálisis facial derecha y Glasgow 14. Ingresa para valoración neurológica, iniciando tratamiento empírico por sospecha de neuroinfección. La punción lumbar reporta proteínas 381 mg/dL, glucosa 6,4 mg/dL, celularidad de 532 células/mm^3^ (95% linfocitos), pruebas de FilmArray y cultivo negativas. La RMN cerebral muestra lesiones hiperintensas en secuencia FLAIR; se sospecha encefalomielitis aguda diseminada (EMDA) se inicia metilprednisolona (30 mg/kg/día por 5 días). El 26.11.24 se sospecha de muerte encefálica por presentar ausencia de reflejos del tallo y de defensa, se realiza nueva RMN el 29/11/2024 evidenciando hidrocefalia, evolución no esperada en la EMDA. Se solicita GeneXpert en LCR y se coloca derivación ventriculoperitoneal. El resultado positivo para Mycobacterium tuberculosis en LCR se obtiene el 03/12/2024. Se inicia esquema DOTBAL (fase intensiva) más esteroides. A pesar del manejo instaurado, el paciente desarrolla deterioro hemodinámico y ventilatorio progresivo mas estado vegetativo persistente. Fallece tras 36 días hospitalizado. **Conclusiones**: La TBM representa un reto diagnóstico en pediatría. La implementación temprana de pruebas como GeneXpert y la sospecha clínica fundamentada son clave para reducir morbimortalidad.

**Palabras Clave:**

tuberculosis meníngea; GeneXpert; hidrocefalia; resonancia magnética; pediatría

**Síndrome Regresivo en la Infancia: Dos Casos de Leucodistrofia Autosómica Recesiva por Mutación en el Gen *ACER3* Diagnosticados por Exoma**

Soto Macedo Andrea Sofia^1^, Vázquez Briseño J.Jesús^1^

^1^Hospital Regional ISSSTE León, 37520 León de los Aldama, México

**Introducción**: El gen *ACER3* codifica la enzima ceramidasa alcalina, que convierte ceramida-esfingosina. La mutación del ACER3 acumula esfingolípidos y ceramidas de cadena larga en cerebro y endotelio, causa desmielinización de sustancia blanca. **Objetivo**: Contribuir al conocimiento de las mutaciones en el gen ACER3 y la leucodistrofia autosómica recesiva, discutir las implicaciones clínicas para el diagnóstico, manejo clínico y orientación médica. **Resumen Clínico del Caso Con Resultado de Estudios, Tratamiento Instaurado y Evolución**: Paciente 1, femenino 18 meses, con retraso del neurodesarrollo, cuadriparesia discinética-distónica y en RMN hipoplasia pontocerebelosa y del cuerpo calloso, con lesiones hiperintensas periventriculares occipitales en T2/FLAIR. Secuenciación de exoma con variante de significado incierto en el gen *ACER3*, relacionado con leucodistrofia progresiva de aparición temprana en la infancia. Paciente 2, femenino de 3 años, con regresión del neurodesarrollo, cuadriparesia mixta espástica-distónica, epilepsia e hiperintensidades periventriculares en RMN. Secuenciación de exoma con 2 variantes de significado incierto en el gen *ACER3*. **Conclusiones**: Ambas pacientes presentaron alteración en el neurodesarrollo, regresión y retraso que comenzó entre los 5 y 12 meses, incluyendo hipotonía troncal, espasticidad, distonía y epilepsia. Las pacientes fueron compatibles con esta condición. La evolución de la enfermedad es lenta y progresiva, puede llevar a una detención completa del desarrollo y falla multiorgánica. Esta condición es autosómica recesiva, hay degeneración de la mielina del sistema nervioso central. El inicio de la enfermedad ocurre en la infancia temprana. Aparentemente los niveles elevados de ceramidas en el sistema nervioso central son la causa de desmielinización aberrante, lo que produce regresión del neurodesarrollo.

**Palabras Clave:**

RMN, resonancia magnética; SNC, sistema nervioso central; leucodistrofia

**Características de Cefalalgia en Pacientes con Absceso Cerebral Intracraneal Secundario a Sinusitis Complicada**

Pérez Parra Jesús Elmer^1^, Gutiérrez Ramírez Diana Laura^1^, Narváez González Diana Elisa^1^, Nava Rodríguez Nelly Marlen^1^, Cantú Salinas Adriana Carlota^1^, Chávez Luevanos Beatriz Eugenia^1^

^1^Hospital Universitario, “Dr. José Eleuterio González”, Universidad Autónoma de Nuevo León, 64460 Monterrey, México

**Introducción**: Los senos paranasales y su proximidad con el encéfalo y los ojos exponen a los pacientes a complicaciones graves con rapidez. La prevalencia de complicaciones neurológicas por sinusitis no está descrita en la literatura actual. Aunados datos faltantes para identificar una sinusitis temprana y consideramos a la cefalea un síntoma pivote. La tercera edición de la Clasificación internacional de las cefaleas (ICHD-III) presenta los criterios de cefalea atribuida a sinusitis aguda en adultos, dejando de lado población pediátrica, nuestra información podría brindar datos clave en este grupo. En adultos, la cefalalgia suele percibirse en la región frontal o facial, pero se irradia a regiones posteriores, ipsilaterales a la sinusitis. **Objetivo**: Describir las características cefalálgicas de 4 pacientes con sinusitis aguda que presentaron complicaciones neurológicas, con el fin de orientar un diagnóstico oportuno. **Resumen Clínico del Caso Con Resultado de Estudios, Tratamiento Instaurado y Evolución**: Se evaluaron 4 pacientes con complicaciones neurológicas por sinusitis aguda, presentaron cefalea de inicio súbito (n = 4), intensidad de moderada a severa, la zona afectada con cefalea fue adyacente a la lesión intracraneal (n = 3), cefalea con banderas rojas (n = 1), evaluaciones médicas previas (n = 4), focalización neurológica (n = 3), fiebre acompañante (n = 4), alteración oftalmológica (n = 1), traumatismo craneoencefálico leve con cefalea y focalización neurológica (n = 2), neuroimagen con absceso epidural (n = 1), el cual no presentó focalización neurológica, absceso epidural y subdural (n = 1) y absceso intracraneal (n = 2). **Conclusiones**: La presencia de cefalea ante un paciente con sinusitis, de intensidad alta, súbita y asociada a fiebre, requiere abordaje de complicaciones intracraneales, con la finalidad de dar un tratamiento oportuno y evitar secuelas importantes.

**Palabras Clave:**

cefalea; sinusitis; fiebre; absceso; empiema

**Epilepsia y Leucoencefalopatía Progresiva Asociadas a Variante Patogénica del Gen *AARS***

Sánchez Encarnación Rocío^1^, Munive Báez Leticia^1^, Medellín López Haideé Deyanira^1^

^1^Instituto Nacional de Pediatría (INP), 04530 Ciudad de México, México

**Introducción**: Las epilepsias y leucoencefalopatías progresivas son poco frecuentes, de etiología diversa entre ellas las alteraciones de micromoléculas. Las alteraciones del gen *AARS*, afectan la codificación de alanyl-tRNA sintetasa mitocondrial, desarrollando deterioro neurológico progresivo, epilepsia, disautonomía, leucoencefalopatía, cardiopatías. Se presenta el caso de femenino de 17 años de edad, con epilepsia focal y leucoencefalopatía progresiva secundaria a variante patogénica del gen *AARS*, con latencia diagnóstica prolongada. **Objetivo**: Reportar las manifestaciones clínicas, paraclínicas y dificultades diagnósticas de paciente con epilepsia focal y leucoencefalopatía progresiva asociadas a variante patogénica del gen *AARS*. Estudio descriptivo, observacional, retrospectivo, transversal. Reporte de caso clínico. **Resumen Clínico del Caso Con Resultado de Estudios, Tratamiento Instaurado y Evolución**: Paciente previamente sana, inició a los 6 años con epilepsia no motora, seguimiento en el INP a los 8 años realizando múltiples EEG observando actividad epileptiforme focal temporal posterior, parietal, occipital en hemisferio cerebral izquierdo, RM cerebral (2016) T2 y Flair: lesiones hiperintensas en sustancia blanca bilaterales, se abordan causas infecciosas, metabólicas, autoinmunes: anticardiolipinas en zona gris, se realizó panangiografía cerebral (2020) reportando vasculitis de arterias carótidas y vertebrales, inmunología aplica múltiples inmunosupresores sin mejoría, en el 2024 se actualiza abordaje diagnóstico considerando epilepsia focal, leucoencefalopatía y disautonomía, se solicitó panel genético encontrando variante patogénica heterocigota del gen *AARS* en la paciente y en el padre, se realiza retiro de inmunosupresores, ajuste de dosis de levetiracetam, oxcarbazepina, se agrega bisoprolol y escitalopram, actualmente paciente sin deficit cognitivo, motor ni sensitivo, en control clinico de la epilepsia y disautonomía. **Conclusiones**: Los estudios paraclínicos son importantes en el diagnóstico, siempre direccionados a las manifestaciones principales, no convertirse en distractores. No se debe establecer un diagnóstico etiológico ni dar tratamiento dirigido sin tener la evidencia suficiente. Los diagnósticos diferenciales permiten considerar etiologías poco frecuentes (genéticas), evitando diagnósticos y tratamientos equivocados.

**Palabras Clave:**

epilepsia; encefalopatía epiléptica; leucoencefalopatía genética; AARS

**Meningoencefalitis por SARS-CoV-2: La Nueva Gran Simuladora**

Marco Antonio Vidal-Peregrina^1^, Fabiola Marycruz De la Fuente-Silva^1^, Maria Fernanda Pérez-Álvarez^1^, Salvador Jiménez-Mejines^1^, Daniela Naomi Ortiz-Fernández^1^, Bianca Maravi Pale-Campomanes^1^, JosuéNava-García^1^, Martín Arturo Silva-Ramírez^1^, Flora Cebada-López^1^

^1^instituto Mexicano del Seguro Social, UMAE Hospital General “Dr. Gaudencio González Garza” Centro Médico Nacional La Raza, 02990 Ciudad de México, México

**Introducción**: El coronavirus del Síndrome Respiratorio Agudo Grave de tipo 2 (SARS-CoV-2) puede afectar al Sistema Nervioso Central por diseminación hematógena o diseminación retrógrada de neuronas en forma indirecta, sin embargo, aún no se descubre el mecanismo exacto. El 20% de los pacientes con SARS-CoV-2 pueden presentar síntomas neurológicos severos, siendo una de ellas la Meningoencefalitis. **Objetivo**: Presentación de caso de Meningoencefalitis por SARS-CoV-2. **Resumen Clínico del Caso Con Resultado de Estudios, Tratamiento Instaurado y Evolución**: Masculino de 13 años. COVID hace 2 años. Inicia con cefalea, fiebre, tratamiento sintomático, se agrega vértigo, dolor abdominal, hiporexia, tratado con Ceftazidima. Acude a hospital particular 5 días después, donde presenta crisis tónica generalizada en 2 ocasiones, tratado con benzodiacepina y Fenitoína, TC de cráneo normal, laboratorios con pancitopenia, transaminasemia, enviado a nuestra Unidad encontrándose febril, con somnolencia, punción lumbar con proteínas 262, glucosa 43,8, células sin reactivo, FilmArray negativo. A los 3 días se agrega irritabilidad, disartria, fotofobia, parálisis de VI nervio craneal bilateral, diplopía, temblor de intención, dismetrías y disdiadococinesias, se repite prueba rápida para SARS-CoV-2 negativa, Ferritina elevada, trasladado a Infectología Pediátrica donde alterna con irritabilidad y estupor, crisis epilépticas, tratado con Levetiracetam, Vitamina K, plasma y concentrado plaquetario, se sospecha en Síndrome Multisistémico Inflamatorio Pediátrico, ecocardiograma normal, tratado con Inmunoglobulina sin mejoría. Se realiza nueva punción lumbar, células 48, polimorfonucleares 60%, proteínas 119,7, glucosa 39, se sospecha en Tuberculosis, tratamiento con DOTBAL + Dexametasona por 3 días presentando mejoría en estado de alerta, se suspende DOTBAL al contar con GeneXpert negativo, anticuerpos neutralizantes contra SARS-CoV-2 en líquido cefalorraquídeo (LCR) positivos, remisión de parálisis de VI a los 12 días. RM de cráneo con leptomeningitis frontal e insular de predominio izquierdo, neuritis del VI nervio izquierdo, microadenoma hipofisiario hemorrágico, se suspende Fenitoína. Seguimiento en Consulta Externa sin focalización, EEG y RM neuroeje normal. **Conclusiones**: La Meningoencefalitis por SARS-CoV-2 es una complicación frecuente, sin embargo, en la mayoría de los casos no se logra aislar el virus en LCR, siendo éste el primer caso reportado en Pediatría. Es fundamental realizar un amplio abordaje diagnóstico para excluir otras causas, ya que el SARS-CoV-2 hoy en día, puede ser la nueva gran simuladora.

**Palabras Clave:**

meningoencefalitis; SARS-CoV-2; COVID-19; pediatría

**Síndrome de Nabais Sá-de Vries: Reporte de Caso Clínico**

Brenda Irais Alvarez-González^1^, Antonio Bravo-Oro^1^, Jorge Luis García-Ramírez^1^

^1^Hospital Regional de Alta Especialidad, “Dr. Ignacio Morones Prieto”, 78290 San Luis Potosí, México

**Introducción**: El síndrome de Nabais Sá-de Vries (NSDVS) es un trastorno genético con patrón de herencia autosómico dominante descrita en 2020, con dos subtipos: NSDVS1 y NSDVS2. Hasta ahora, solo se habían reportado 6 casos con 5 variantes sin sentido que afectan la función de la proteína SPOP, clave en la degradación de proteínas. En 2020, Nabais Sá y colaboradores identificaron mutaciones esporádicas en SPOP en siete niños con discapacidad intelectual y del desarrollo, dando lugar a un nuevo síndrome: el síndrome de Nabais Sá-de Vries. **Objetivo**: Describir dos casos clínicos de síndrome de Nabais Sa-de Vries con el objetivo de revisar e identificar las características clínicas y neurológicas de la enfermedad, así como su relación con trastornos del neurodesarrollo. **Resumen Clínico del Caso Con Resultado de Estudios, Tratamiento Instaurado y Evolución**: Caso 1: paciente masculino de 4 años de edad quien acude a valoración a los 14 meses de edad por retraso del neurodesarrollo y presentar conductas de riesgo de trastorno del espectro autista, a los 3 años de edad se realiza diagnóstico de TEA. Caso 2: paciente masculino de 8 años con antecedente de comunicación intraventricular, hipoacusia bilateral, discapacidad intelectual y paladar hendido. Se solicitó valoración de los dos casos por genética realizándoles secuenciación exómica. Se identificaron en ambos casos variantes en heterocigosis en el gen *SPOP*, las variantes de este gen son asociadas al síndrome Nabais Sa-de Vries autosómico dominante. **Conclusiones**: Se han reportado pocos casos del Síndrome de Nabais Sa-de Vries por lo que es fundamental llevar a cabo estudios adicionales para esclarecer la correlación entre el genotipo (tipo de mutación en SPOP) y el fenotipo clínico.

**Palabras Clave:**

SPOP; trastornos del neurodesarrollo; discapacidad intelectual; autosómica dominante; síndrome de Nabais Sá-de Vries

**Enfermedad de Moyamoya, Reporte de Dos Casos**

Gutiérrez Soto-Marisol^1^, López Gallegos-Gabriela Monserrat^1^, Reyes Cuayahuitl-Araceli^1^, Rayo Mares-Jesús Darío^1^

^1^Hospital de Pediatría CMN Siglo XXI, IMSS, 06720 Ciudad de México, México

**Introducción**: La enfermedad de Moyamoya es un desorden cerebrovascular idiopático, poco frecuente, presenta estenosis bilaterales de las arterias carótidas internas, sus ramas principales y neoformación de vasos. Se presenta entre los 5 y 40 años. En los niños los cuadros son de tipo isquémico. La RNM ayuda en gran medida al diagnóstico. La revascularización quirúrgica constituye la intervención más eficaz para mejorar la perfusión cerebral y prevenir la progresión de la enfermedad. **Objetivo**: Describir la presentación clínica, electroencefalográficas y estudios de imagen de pacientes con enfermedad de Moyamoya. **Resumen Clínico del Caso Con Resultado de Estudios, Tratamiento Instaurado y Evolución**: Caso 1: masculino de 9 años. Asfixia al nacimiento. Al año de edad con movimientos clónicos en extremidad superior izquierda. TAC de cráneo con lesión hipodensa en lóbulo frontal derecho y lóbulos temporales, parietal, en relación al territorio de la arteria cerebral media derecha. RNM, lesiones hipointensas en lóbulo temporal, defecto de llenado de ambas arterias carótidas internas. A la exploración con hemiparesia derecha y REMS aumentados. En tratamiento con ácido acetil salicílico. Caso 2: masculino de 10 años de edad. Prematuro. Diagnóstico de síndrome de Down y PCA. Inicia a los 7 años con disminución de fuerza en hemicuerpo derecho, alteración en lenguaje y movimientos clónicos en extremidad superior izquierda. EEG con ondas de alto voltaje en región frontal derecha. RNM de encéfalo y angioresonancia múltiples zonas de EVC isquémico. Fuera de tratamiento para revascularización. A la exploración funciones mentales alteradas, tono disminuido global, REMS aumentados, no realiza marcha. **Conclusiones**: La enfermedad de Moyamoya en la infancia representa una causa importante, aunque poco frecuente, de enfermedad cerebrovascular isquémica. Su reconocimiento temprano es esencial para evitar daño neurológico irreversible, ya que el curso clínico puede ser silencioso o manifestarse inicialmente con episodios transitorios de déficit neurológico. El seguimiento neurológico estrecho es clave, dada su evolución impredecible y su potencial para generar discapacidad neurológica significativa si no se interviene a tiempo.

**Palabras Clave:**

Moyamoya; enfermedad cerebrovascular; evento vascular isquémico; déficit neurológico; vasculopatía oclusiva crónica

**Evento Stroke-Like en Paciente con Sindrome de Melas: Reporte de Caso**

González González Raúl Humberto^1^, Hernandez González Claudia Nelly^1^, Flores Alfaro Fernanda^1^, Saldivar Dávila Sergio^1^, Vázquez Fuentes Salvador^1^, Chávez Luevanos Beatriz Eugenia^1^

^1^Servicio de Neurología, Hospital Universitario “Dr. José Eleuterio González”, Universidad Autónoma de Nuevo León, 64460 Monterrey, México

**Introducción**: El síndrome de encefalomiopatía mitocondrial con acidosis láctica y eventos Stroke-like (MELAS) es un trastorno mitocondrial poco frecuente que presenta disminución de la vía aerobia e incremento de la producción de energía por la vía anaerobia, generando niveles elevados de concentración de lactato; y que generalmente se presenta con episodios stroke-like los cuales son visibles en imagen utilizando resonancia magnética nuclear de cerebro (RMN) observando imágenes sugestivas de evento isquémico que no respetan territorios vasculares. **Objetivo**: Describir un caso de episodio Stroke-like en una paciente con diagnóstico de síndrome de MELAS. **Resumen Clínico del Caso Con Resultado de Estudios, Tratamiento Instaurado y Evolución**: Femenina de 10 años de edad, con antecedente de diagnóstico de Síndrome de MELAS un año previo; iniciando con retraso global del neurodesarrollo en la infancia, debilidad muscular generalizada y múltiples episodios de cefalea holocraneana intensa. Sospechándose el diagnóstico por clínica y una RMN de cerebro con espectroscopía y con confirmación de la variante del gen MT-TL1, siendo esta la más común, presentándose hasta en el 80% de los casos. Acudió a valoración por 6 horas de evolución con paresia en extremidad superior derecha rápidamente progresiva, acompañada de cefalea holocraneana opresiva de severa intensidad. A su valoración se observó paresia, hiperreflexia e hipoestesia en extremidad superior izquierda y lactacidemia. Se realizo una RMN identificando una hiperintensidad en secuencia FLAIR en giro post-central derecho con restricción a la difusión, así como un pico de lactato en la espectroscopia de la lesión. **Conclusiones**: Se diagnostica con un evento stroke-like en región parietal derecha y se inicia manejo con L-arginina y levetiracetam como medicamento anticrisis. Presenta una evolución adecuada con resolución de la sintomatología para las 48 horas posteriores a su ingreso y se egresa a las 72 horas con manejo con suplementación con L-arginina y el mismo medicamento anticrisis y seguimiento multidisciplinario por la consulta externa.

**Palabras Clave:**

MELAS; stroke-like; mitocondrial

**Perfil Clínico del Síndrome de Lennox-Gastaut en el Instituto Nacional de Pediatría: Serie de Casos**

Jorge Luis Sánchez Vargas^1^, Matilde Ruiz García^1^

^1^Instituto Nacional de Pediatría (INP), 04530 Ciudad de México, México

**Introducción**: El síndrome de Lennox-Gastaut (SLG) es una encefalopatía epiléptica pediátrica grave, caracterizada por múltiples tipos de crisis, deterioro cognitivo y un patrón EEG distintivo de punta-onda lenta. Su inicio suele ser antes de los 8 años y se asocia con etiologías diversas como alteraciones genéticas, estructurales o perinatales. El manejo representa un reto clínico debido a su alta refractariedad y elevada carga neurocognitiva y funcional. **Objetivo**: Describir las características clínicas, diagnósticas y terapéuticas de pacientes con SLG atendidos en el Instituto Nacional de Pediatría entre 2014 y 2024. **Resumen Clínico del Caso Con Resultado de Estudios, Tratamiento Instaurado y Evolución**: Material y Métodos: Estudio observacional, descriptivo y retrospectivo tipo serie de casos. Se incluyeron seis pacientes con diagnóstico confirmado de SLG. Se analizaron variables clínicas, tipos de crisis, resultados de EEG, neuroimagen, comorbilidades, tratamientos y evolución clínica. **Resultados**: Cinco de los seis pacientes fueron varones (83,3%), con inicio de crisis entre los 2 y 5 años. Todos presentaron epilepsia refractaria y requirieron politerapia; solo un paciente logró estar libre de crisis. Los hallazgos en EEG fueron diversos, predominando anomalías inespecíficas (50%) y punta-onda lenta (33,3%). La mayoría presentó discapacidad intelectual, especialmente en su forma leve (50%). Las comorbilidades sistémicas más frecuentes fueron desnutrición y afecciones pulmonares y cardiovasculares. En neuroimagen se reportó atrofia cerebral (33,3%) y displasia cortical (16,7%). **Conclusiones**: El SLG es una condición neurológica severa, con inicio temprano, alta refractariedad al tratamiento y un importante impacto en el desarrollo cognitivo. A pesar de la politerapia, la mayoría de los pacientes presentan control parcial o nulo de las crisis. La identificación temprana y el enfoque multidisciplinario son esenciales para mejorar el pronóstico y la calidad de vida.

**Palabras Clave:**

epilepsia refractaria; encefalopatía epiléptica; Lennox-Gastaut; politerapia; discapacidad intelectual

**Uso de Sirolimus Prenatal y Vigabatrina Postnatal en Complejo de Esclerosis Tuberosa: Reporte de Caso**

María de los Ángeles Olivares-Gutiérrez^1^, Iliana Duron-Tabora^1^, Salvador Vásquez-Fuentes^1^, Adriana Carlota Cantú-Salinas^1^, Beatriz Eugenia Chávez-Luévanos^1^, Ana Luisa Carrión-Garcia, De la Garza Oscar^1^

^1^Servicio de Neurología, Servicio de Neonatología, Hospital Universitario “Dr. José Eleuterio González”, 64460 Monterrey, México

**Introducción**: Complejo de Esclerosis Tuberosa (CET) trastorno neurocutáneo, afectando 1:6000 nacidos vivos; autosómica dominante, generando tumoraciones en diferentes órganos como corazón y cerebro, generando nódulos subependimarios. 90% de manifestaciones neurológicas pueden ser epilepsia refractaria, principalmente síndrome de espasmos infantiles, trastorno del neurodesarrollo, deterioro cognitivo, autismo y problemas de aprendizaje, que determinan su pronóstico; afectando gravemente la calidad de vida. Lactantes con CET experimentan diversos tipos de crisis epilépticas, principalmente espasmos epilépticos y el tratamiento de epilepsia asociada a CET sigue siendo desafío médico. **Objetivo**: Uso de Sirolimus prenatal y vigabatrina postnatal en complejo de esclerosis tuberosa. **Resumen Clínico del Caso Con Resultado de Estudios, Tratamiento Instaurado y Evolución**: Masculino de 8 meses, manejo prenatal adecuado, diagnosticando a las 28,1 SDG rabdomioma y derrame pericárdico; resonancia magnética (RM) fetal corroborando rabdomioma cardiaco, túberes cerebrales múltiples frontal-temporal-occipital bilaterales. Iniciándose sirolimus prenatalmente. Nació vía cesárea a las 39 SDG, Apgar y antropometría adecuada. Ingresó a cuidados intensivos neonatales para abordaje con ecocardiograma y RM cardiotorácica visualizando rabdomioma intramural interventricular. RM cerebral lesiones hipointensas múltiples en sustancia blanca en T1 e hiperintensas en T2 correspondiendo a múltiples nódulos subependimario y FLAIR subcorticales fronto-parietales y temporooccipital, además calcificaciones por técnica SAWN. 4to día de vida, Inició sirolimus, presentando sangrado tubo digestivo alto, omitiendo tratamiento, sustituido por Everolimus, agregándose al mes de vida vigabatrina de forma profiláctica. Egresando con everolimus y vigabatrina y electroencefalograma mensualmente donde no se evidencia patrón de hipsarritmia ni actividad epiléptica. Paciente de 8 meses sin alteración en neurodesarrollo, ni oftalmológica, no espasmos, No crisis epilépticas, continua en seguimiento neurológico y multidisciplinario. **Conclusiones**: El diagnóstico y la intervención temprana con sirolimus y vigabatrina ayudan reduciendo la aparición de epilepsia farmacorresistente como alteraciones en neurodesarrollo. Importante mejorará significativamente la calidad de vida de pacientes con CET y reducirá cargas familiares y sociales. Vigabatrina significativamente relacionada con mejora de la cognición y comportamiento en individuos con CET, como preventivo, puede retrasar aparición de crisis y riesgo de encefalopatía epiléptica. La eficacia de la inhibición de mTOR con everolimus complementario se ha documentado en pacientes con epilepsia farmacorresistente en CET.

**Palabras Clave:**

complejo de esclerosis tuberosa; prenatal; postnatal; sirolimus; everolimus; vigabatrina

**Polineuropatía Aguda Como Manifestación de Porfiria Aguda Intermitente: Reporte de Caso**

Cruz Mejía Saytin Gabriela^1^, Vallecillo Amador Len Isaías^1^, Medellín López Haideé Deyanira^1^, Ruiz García Matilde^1^, Hernández Antúnez Blanca Gloria^1^

^1^Departamento de Neurología Pediátrica, Instituto Nacional de Pediatría, 04530 Ciudad de México, México

**Introducción**: Las polineuropatías son trastornos caracterizados por el daño a nervios periféricos, sus causas incluyen enfermedades metabólicas, infecciosas, autoinmunes, tóxicas y hereditarias. La porfiria aguda intermitente (PAI), es una causa metabólica rara, que ocasiona deficiencia enzimática del grupo hemo, que puede provocar crisis neuroviscerales agudas y polineuropatía progresiva. Su presentación clínica puede simular patologías más frecuentes, como el síndrome de Guillain-Barré (SGB). **Objetivo**: Describir el caso de un paciente con polineuropatía aguda secundaria a PAI. **Resumen Clínico del Caso Con Resultado de Estudios, Tratamiento Instaurado y Evolución**: Paciente femenina de 16 años ingresa por dolor abdominal, vómitos, hipertensión arterial y crisis epilépticas asociadas a hiponatremia severa, se agrega debilidad en extremidades de predominio proximal y simétrica, acompañado de parestesias. Se realizan velocidades de conducción nerviosa (VCN) que evidencian neuropatía axonal motora y sensitiva, disminución de amplitud, velocidades y respuesta F normal. Debilidad progresa rápidamente y sufre depresión respiratoria, requiriendo ventilación mecánica invasiva. Inicialmente se sospecha SGB, sin embargo, ante clínica previamente presentada, VCN y patrón de debilidad, se considera como diagnóstico diferencial PAI. Se realiza prueba de Hoesch con resultado positivo y la orina expuesta a luz ultravioleta muestra coloración naranja fluorescente. Se confirma diagnóstico con niveles de porfobilinógeno en orina de 379,7 µmol/L (valor de referencia ≤1,3 µmol/L). Se inicia tratamiento con hemina intravenosa y plasmaféresis. Actualmente la paciente presenta cuadriparesia. **Conclusiones**: La PAI tiene una baja incidencia mundial, es más común en mujeres y los principales desencadenantes de crisis son medicamentos, infecciones, alcohol y cambios hormonales. La PAI es conocida como la gran imitadora debido a su heterogeneidad clínica. El patrón de debilidad y VCN ayudan a diferenciarla. El cambio en coloración de la orina ante su exposición a la luz es un hallazgo característico de los ataques agudos, siendo crucial para el diagnóstico del caso.

**Palabras Clave:**

porfiria aguda intermitente; síndrome de Guillain Barré; polineuropatía; parálisis flácida aguda; hemina

**Miastenia Gravis Pediatrico con Debut Ocular Unilateral con Presencia de Timoma**

Iván Humberto Ortega García^1^, Gerardo Enrique González Salazar^1^

^1^Hospital Infantil de Tamaulipas, 87070 Cdad. Victoria, México

**Introducción**: La Miastenia Gravis es una enfermedad neuromuscular autoinmune caracterizada por debilidad muscular fluctuante, que empeora con el ejercicio y mejora con el descanso. Con una incidencia juvenil del 10–15% en Norteamérica. La presentación más frecuente es ocular, con diplopía y ptosis la cual puede ser unilateral o bilateral pero típicamente es simétrica. La debilidad puede variar a lo largo del día. El diagnóstico se confirma mediante serología, detectando anticuerpos contra receptores de acetilcolina mayores a 0,64 nmol/L. La clasificación se basa en distribución de síntomas, severidad, serología y estado tímico. El pronóstico depende de la respuesta al tratamiento. **Objetivo**: (1) Identificar características clínicas y evolución de Miastenia Gravis pediátrica con debut ocular unilateral asociado a timoma en pacientes infantiles. (2) Evaluar estudios diagnósticos y estrategias terapéuticas en niños con Miastenia Gravis ocular unilateral y hallazgo de timoma en imagenología. **Resumen Clínico del Caso Con Resultado de Estudios, Tratamiento Instaurado y Evolución**: Femenina de 7 años con ptosis palpebral derecha y diplopía fluctuante, con mejoría matutina y evolución de dos semanas. Sin antecedentes familiares ni prenatales relevantes. Nacida por parto vaginal a término, Apgar 9, con hospitalización en UCIN por tres días por mala adaptación pulmonar. Desarrollo psicomotor y vacunación completos. En exploración, Glasgow de 15, orientada, con movimientos oculares incompletos en los cuatro cuadrantes del ojo derecho, ptosis palpebral derecha y alteraciones en los pares craneales III y IV. El resto del examen neurológico fue normal. Se realizaron prueba de compresa fría y prueba de neostigmina, ambas positivas. Los anticuerpos anti-acetilcolina fueron elevados (2,64). La tomografía de tórax evidenció una tumoración en el timo, compatible con Miastenia Gravis, confirmando el diagnóstico clínico-serológico con afectación ocular inicial y hallazgo asociado de timoma. **Conclusiones**: La Miastenia Gravis pediátrica con debut ocular unilateral y timoma es una condición poco común, pero importante de reconocer tempranamente. Este resalta la relevancia de una evaluación clínica y diagnóstica completa, incluyendo estudios serológicos e imagenológicos, para un diagnóstico oportuno que permita un tratamiento adecuado y mejore el pronóstico.

**Palabras Clave:**

Miastenia Gravis juvenil; pediatría; ocular; ptosis unilateral

**Cavernomatosis Múltiple: Tratamiento de Epilepsia Estructural en Lactante**

María de los Ángeles Olivares-Gutiérrez^1^, Ana Luisa Carrión-Garcia^1^, Adriana Carlota Cantú-Salinas^1^, José Arenas-Ruiz^1^, José Bonilla-Galván^1^, Oscar De la Garza-Pineda^1^, Beatriz Eugenia Luevanos-Chávez^1^

^1^Servicio de Neurología, Servicio de Neurocirugía, Hospital Universitario “Dr. José Eleuterio González”, Universidad Autónoma de Nuevo León, 64460 Monterrey, México

**Introducción**: La Cavernomatosis Múltiples (CM) son lesiones hamartomatosas formadas por sinusoides dilatados, sin parénquima cerebral interpuesto, con alteración del desarrollo de red arteriolocapilar, la mayoría son esporádicas, caracterizado por la presencia de CM en un solo paciente. Afectan 0,4–0,8% de población. Con clínica variable, realizando evaluación exhaustiva del perfil de tolerancia al riesgo del paciente, para la toma de decisiones terapéuticas multidisciplinaria. **Objetivo**: Analizar el abordaje de la epilepsia estructural en un lactante con la cavernomatosis múltiple. **Resumen Clínico del Caso Con Resultado de Estudios, Tratamiento Instaurado y Evolución**: Lactante masculino de 13 meses de vida, con neurodesarrollo adecuado, que en mayo 2024 presentó estado epiléptico focal clónico en mano y pie izquierdo, con alteración estado de alerta; cediendo con levetiracetam, requiriendo se trasladado a cuidados intensivos, realizándose Resonancia Magnética Cerebral (RMC) la cual reportó cavernomatosis múltiple, con sangrado subagudo en región frontal derecha. Video-Electroencefalograma: actividad epileptiforme en región frontal derecha. Manejo quirúrgico: craneotomía frontoparietal derecha; localizando la lesión con ultrasonido transoperatorio, realizándose mapeo de áreas frontales elocuentes, colocando malla de registro, utilizando abordaje transulcal parafascicular, resecan cavernoma y área de gliosis adyacente con control electrocorticográfico; egresando sin complicaciones, actualmente se encuentra libre de crisis epilépticas desde posterior a resección quirúrgica, en sus seguimientos médicos se encuentra con neurodesarrollo adecuado para la edad. **Conclusiones**: El tratamiento integral, oportuno y adecuado, de la epilepsia por cavernomatosis multiple mejora el pronóstico neurológico del paciente. Las Lesiones múltiples son el distintivo de la forma familiar. La imagen de elección en CM es RMC. Cuando sea posible, la resección quirúrgica es la opción de tratamiento preferida en CM sintomática y en casos seleccionados, radioterapia dirigida para tratar lesiones. Se requerirá de pruebas genéticas en paciente y progenitores para determinar origen de lesiones, por ser autosómica dominante.

**Palabras Clave:**

cavernomatosis multiple; epilepsia estructural; resección quirúrgica

**Importancia del Abordaje Diagnóstico en el Debut de Enfermedad Desmielinizante en Niños. Reporte de 2 Casos**

Daniela Méndez-Fernández^1^, Martín Arturo Silva-Ramírez^1^, Fabiola Marycruz De la Fuente-Silva^1^, Flora Cebada-López^1^

^1^Instituto Mexicano del Seguro Social, UMAE Hospital General “Dr. Gaudencio González Garza” CMN La Raza, 02990 Ciudad de México, México

**Introducción**: Los trastornos desmielinizantes del Sistema Nervioso Central (SNC) tienen amplio espectro de fenotipos clínicos e imagenológicos: encefalomielitis aguda diseminada, neuritis óptica, mielitis transversa, neuromielitis óptica y esclerosis múltiple, teniendo como común denominador la afectación inflamatoria de la mielina del encéfalo, médula espinal, nervios ópticos, debido a una respuesta inflamatoria mediada por autoanticuerpos. **Objetivo**: Destacar la importancia de un adecuado abordaje diagnóstico en enfermedad desmielinizante en pacientes pediátricos. **Resumen Clínico del Caso Con Resultado de Estudios, Tratamiento Instaurado y Evolución**: Caso 1. Adolescente femenino de 17 años. Inicia con monoparesia extremidad inferior derecha con recuperación; a los 2 meses presenta monoparesia de extremidad inferior izquierda, a la exploración física con síndrome de neurona motora superior ambas extremidades inferiores, agudeza visual ojo derecho 20/70; se inició abordaje con TC con lesión hipodensa a nivel de lóbulo frontal; bandas oligoclonales en líquido cefalorraquídeo (LCR) positivas; RM de cráneo con lesiones en sustancia blanca supra e infratentoriales, medulares y nervio óptico derecho engrosado datos sugestivos de Neuromielitis. Caso 2. Adolescente de 12 años. Inicia con amaurosis de ojo derecho y dolor retro ocular de un mes de evolución. Posteriormente cefalea frontal, pulsátil, 9/10, fotofobia, con diagnóstico de neuritis óptica retrobulbar derecha, inicia abordaje con TC de cráneo múltiples lesiones hipodensas en sustancia blanca profunda, RM neuroeje con múltiples lesiones supra e infratentoriales de sustancia blanca, así como en médula espinal sugestivas de Esclerosis Múltiple con datos de actividad de la enfermedad, bandas oligoclonales en LCR positivas. **Conclusiones**: Las bandas oligoclonales positivas en LCR se pueden encontrar hasta en un 30% de casos en Neuromielitis óptica, así como seronegatividad de anticuerpos antiacuaporina 4 en 10–15%. Por otro lado, en Esclerosis Múltiple hasta un 30% de los casos debutan con cuadro de neuritis óptica; por lo cual el diagnóstico de las enfermedades desmielinizantes debe basarse no sólo en el fenotipo clínico, sino también en estudios paraclínicos complementarios y la respuesta terapéutica.

**Palabras Clave:**

neuromielitis; esclerosis múltiple; hemiparesia; enfermedad desmielinizante

**Miopatía Congénita por Desproporción del Tipo de Fibra Secundaria a Mutación en Gen *ACTA1*: Reporte de Caso**

Cetina Gómez Héctor^1^, Espinoza Zacarías Juan Pedro^1^, Ruiz Ferreira Maria Soledad^1^

^1^Hospital Regional ISSSTE, “Licenciado Adolfo López Mateos”, 01030 Ciudad de México, México

**Introducción**: Las miopatías congénitas son trastornos hereditarios raros del músculo esquelético. Se caracterizan por un inicio neonatal, con hipotonía y debilidad de predominio axial, proximal y facial, retraso en hitos del neurodesarrollo. En sus formas graves presentan alta mortalidad en los primeros años de vida por compromiso de la musculatura respiratoria, así como miocardiopatía. Tienen una prevalencia de 1:25.000, representando 14% de todos los casos de hipotonía neonatal. La clasificación reporta 5 tipos: miopatía nemalínica, miopatía centronuclear, miopatía con cores, miopatía por desproporción congénita del tipo de fibra, miopatía de depósito de miosina. Dichos trastornos involucran a más de 30 genes distintos. La miopatía congénita por desproporción del tipo de fibra es una enfermedad rara en México, con prevalencia de 1:50.000, se debe principalmente a mutación en gen *ACTA1* (codificante de proteína alfa-actina), que interfiere con la contracción muscular. **Objetivo**: Describir el caso de femenino de 3 años con diagnóstico genético de mutación de gen *ACTA1*. **Resumen Clínico del Caso Con Resultado de Estudios, Tratamiento Instaurado y Evolución**: Estudio descriptivo cualitativo retrospectivo tipo reporte de caso basado en expediente clínico. Femenino de 3 años. Antecedentes familiares de muerte neonatal por ambas ramas sin conocer causas. Prima materna con hipotonía al nacimiento, sin más información. Producto de G2, control prenatal adecuado, con USG de séptimo mes que reporta polihidramnios. Amenaza de aborto por sangrado transvaginal manejado con reposo. Se obtiene por cesárea secundaria a polihidramnios, producto femenino que respira y llora al nacer, presenta hipotonía posterior, se egresa en binomio. Presenta retraso del neurodesarrollo, hipotonía persistente. Valorado por neurología pediátrica, con retraso global del neurodesarrollo (Edad: 6 meses, DENVER II: 3 meses). EEG, sin elementos paroxísticos, TAC de cráneo simple sin alteraciones. Estudio genético atrofia muscular espinal: altamente improbable. PEATC: Inmadurez de la vía auditiva bilateral periférica izquierda. RM de encéfalo sin alteraciones. Prueba de Exoma: Gen *ACTA1* positivo. **Conclusiones**: Paciente que presenta variante de una enfermedad rara, con mutación de gen *ACTA1*, patología de la cual no se encuentra disponible una amplia cantidad de literatura en nuestro país.

**Palabras Clave:**

miopatía congénita; desproporción del tipo de fibra; gen ACTA1

**Aciduria Metilglutacónica (Síndrome de Megdel): Reporte de Caso en un Hospital Pediátrico Mexicano de 3er Nivel de Atención**

Sandoval Flores Ivonne^1^, Herrera Mora Patricia^1^, Ruiz García Matilde^1^

^1^Instituto Nacional de Pediatría, 04530 Ciudad de México, México

**Introducción**: La aciduria 3-metilglutacónica constituye un grupo heterogéneo de enfermedades raras y hereditarias que comparten un hallazgo metabólico común: aumento de la excreción en la orina de ácido 3-metilglutacónico. Se han reconocido cinco tipos distintos: la aciduria 3-metilglutacónica tipo I es un error congénito del metabolismo de la leucina; los 4 tipos adicionales afectan la función mitocondrial a través de diferentes mecanismos patológicos. **Objetivo**: Describir el cuadro clínico y evolución de una paciente femenina con diagnóstico de Síndrome de Megdel. **Resumen Clínico del Caso Con Resultado de Estudios, Tratamiento Instaurado y Evolución**: La paciente fue referida a nuestro instituto a los 4 años de edad como sospecha diagnóstica de leucodistrofia y síndrome regresivo al presentar pérdida de los hitos motores y de lenguaje. Cuenta con antecedente de embarazo normoevolutivo, requirió hospitalización durante una semana por sepsis neonatal, tiene hermana con diagnóstico de discapacidad intelectual de etiología no especificada. Se decide su ingreso al servicio de Neurología para abordaje, a la exploración física presentaba poca movilización espontanea en las extremidades, hipotonía axial, piernas con posición en tijeras, hiperreflexia generalizada con clonus espontaneo en miembro inferior izquierdo, postura distónica de tronco con arqueamiento del tórax posterior y cuello, también posturas distónicas de extremidades inferiores de predominio distal. Se realizan estudios de extensión diagnóstica encontrando elevación en orina de ácido 3 metilglutárico y 3 metilglutacónico, panel genético que reporta SERAC1(NM_032861.4):c.1763delT (p.Val588GlyfsTer11) variante significado incierto, con potenciales auditivos con hipoacusia profunda, potenciales visuales con dispersión cortical, se integra el diagnóstico de Aciduria Metilglutacónico, se decide egreso a domicilio después de un mes de hospitalización con gastrostomía y dieta restringida en leucina , así como benzodiacepina y trihexifenidilo. Actualmente con discapacidad intelectual grave y cuadriparesia espástica. **Conclusiones**: A pesar de que el reporte genético se indicó como VUS, se realizó una búsqueda en bases de datos como VarSome donde se encuentra patogénica por ser una variante nula. El retraso psicomotor, la sordera neurosensorial, la distonía y las imágenes cerebrales similares a Leigh fueron los hallazgos característicos presentados en nuestra paciente y en la literatura.

**Palabras Clave:**

deficiencia de 3-metilglutaconil-coenzima hidratasa A (MGH); síndrome de Megdel; gen *SERAC1*

**Reporte del Primer Caso Pediátrico en México de Lipofuscinosis Ceroide Neuronal Tipo 7**

López García Ameyalli^1^, Espinoza Zacarias Juan Pedro^1^, Ruiz Ferreira María Soledad^1^

^1^Hospital Regional “Lic. Adolfo López Mateos”, 01030 Ciudad de México, México

**Introducción**: La lipofuscinosis ceroide neuronal tipo 7 (NCL 7) es un trastorno autosómico recesivo causado por variantes homocigóticas o heterocigóticas bialélicas en el gen *CLN7*/*MFSD8*. **Objetivo**: Describir el cuadro clínico en un paciente pediátrico mexicano con NCL7 confirmado por prueba molecular. **Resumen Clínico del Caso Con Resultado de Estudios, Tratamiento Instaurado y Evolución**: Masculino de 9 años, padres no consanguíneos, sin antecedentes de importancia neurológica o riesgo perinatal. Inicia a los 5 años con inestabilidad de la marcha, lateropulsión indistinta y caídas frecuentes, realiza frases de dos palabras. A los 6 años con disminución en el número de palabras, fijación y seguimiento visual inconstante. A los 7 años con pérdida de la marcha, sedestación y control cefálico. A los 8 años presenta epilepsia de inicio focal con pérdida del estado de consciencia tipo motor tónico- clónico y mioclónico. Movimientos esporádicos coreoatetósicos y temblor de acción en las 4 extremidades. Exploración neurológica: Sin interacción con el medio externo. Emite sonidos guturales. NC II sin fijación ni seguimiento visual, sin respuesta visual a la amenaza NC III, IV, VI mirada conjugada con simetría a la movilidad ocular espontanea, pupilas isocóricas de 3 mm, reflejo fotomotor y consensual presentes. Resto sin alteraciones. Sistema motor: en las cuatro extremidades, hipotrofia, signo de navaja, fuerza muscular proximal, media y distal 4/5 en escala de Daniels. REM’s +++ Respuesta plantar extensora y clonus agotable bilateral. Sensibilidad: retira al estimulo táctil y doloroso. Cerebelo nistagmo horizontal bilateral con movimiento pendular. Extrapiramidal: movimientos esporádicos coreoatetósicos y signo de rueda dentada bilateral. Marcha: No logra. Sin datos meníngeos. Sin estigmas neurocutáneos. Panel genético de epilepsia: MFSD8 c.415>T (p. Arg139cys). **Conclusiones**: Este caso representa el segundo caso documentado en población mexicana pero el primero en paciente pediátrico. La NCL 7 representa un subtipo raro, en el que la acumulación de ceroide y lipofuscina dentro del lisosoma provoca la muerte neuronal. Aun no existe tratamiento curativo, sin embargo, su diagnóstico puede guiar el tratamiento de la enfermedad.

**Palabras Clave:**

lipofuscinosis ceroide neuronal tipo 7; panel genético; gen *MFSD8*

**Encefalitis Autoinmune en Pediatría. Serie de Casos**

Flores Sánchez Zyanya Stephanie^1^

^1^Hospital de Alta Especialidad del Niño “Rodolfo Nieto Padrón”, zip code city, country

**Introducción**: La encefalitis autoinmune (EA) es una inflamación cerebral que afecta que causa disfunción neurológica y síntomas psiquiátricos. Es poco común en población pediátrica, con 20.000 casos anuales en EE. UU., y predomina en niñas. En México, se estima una incidencia de 1,54 casos por millón, asociándose mayormente a infecciones virales y más común por anticuerpos anti NMDA. **Objetivo**: Mostrar las características clínicas, evolución y tratamiento de 4 pacientes con encefalitis autoinmune en periodo de 2 años. **Resumen Clínico del Caso Con Resultado de Estudios, Tratamiento Instaurado y Evolución**: El 75% presentó crisis convulsivas como debut, de los cuales el 50% con estado epiléptico, los signos iniciales más comunes: crisis convulsivas (75%), falta de respuesta al medio (75%), trastornos del habla (75%), trastornos del movimiento (75%). El 100% presentó alteración del estado de alerta con somnolencia, irritabilidad y períodos de alternancia entre estos. El 100% de los pacientes presentaron alteración electroencefalográfica con enlentecimiento, asimetría, actividad epileptiforme y “cepillos delta”. Resonancia magnética: hallazgos más relevantes fueron zonas de hiperintensidad multifocales de predominio frontal y temporal en ponderaciones FLAIR y T2; e hiperintensidad cerebelosa (25%). En el 50% se encontraron anticuerpos anti-NMDA y, 50% tuvo buena respuesta al tratamiento de primera línea con inmunoglobulina y metilprednisolona, mientras que en el 50% se tuvo que recurrir a manejo de segunda línea con rituximab y azatioprina, con adecuada respuesta. **Conclusiones**: La EA es un trastorno neurológico grave mediado por la producción de autoanticuerpos contra componentes del sistema nervioso central, siendo el anticuerpo contra el receptor N-metil-D-aspartato (NMDA) uno de los más comunes en la población pediátrica. En cuanto a la sintomatología característica en la población pediátrica, se presentan inicialmente las manifestaciones neurológicas seguidas de las psiquiátricas. El hallazgo más común son las hiperintensidades de predominio frontal y en el EEG se observa enlentecimiento y presencia de cepillos delta (anti-NMDA). Por último, el tratamiento de primera línea no siempre es efectivo, por lo que se debe recurrir al de segunda línea.

**Palabras Clave:**

encefalitis autoinmune; infecciones virales; crisis convulsivas; somnolencia; cambios conductuales; manifestaciones psiquiátricas; enlentecimiento en electroencefalograma; cepillos delta; anti NMDA

**Epilepsia Focal Lesional: Desafíos y Resultados de la Reseccion Guiada por Electrocorticografia en la Corteza Motora Presentacion de Caso Clinico**

Rodrigo Arispe Adriana Blanca^1^, Flores García Karla Wendoline^1^, Varela Larios Ricardo Jesús^1^, León Reyes Lluvia Itzel^1^, Rodríguez García Verónica^1^, Venta Sobero José Antonio^1^

^1^Centro Médico Nacional 20 de Noviembre, ISSSTE, 03104 Ciudad de México, México

**Introducción**: Paciente femenina de 15 años que debuta a los 9 años con epilepsia focal farmacorresistente que progresa a Epilepsia Parcial Continua (EPC), con crisis de predominio en extremidad inferior derecha y frecuentes episodios de paresia de Todd. Antecedente de estados epilépticos motores con progresión bilateral y deterioro cognitivo progresivo. IRM revela displasia cortical focal (DCF) en giro precentral izquierdo, zona altamente elocuente relacionada al área motora primaria. **Objetivo**: Analizar de forma detallada el abordaje diagnóstico y terapéutico de un caso de EPC, cuyo foco epileptógeno se localiza en la corteza motora primaria del lado izquierdo. Evaluar los beneficios y riesgos inherentes considerando el balance entre el control de crisis y la prevención de déficits neurológicos permanentes. **Resumen Clínico del Caso Con Resultado de Estudios, Tratamiento Instaurado y Evolución**: Revisión de expediente clínico, estudios de imagen (IRM, PET-CT, SPECT ictal), videoelectroencefalogramas ictales, Test de Wada que reportó dominancia lingüística izquierda y electrocorticografía (ECoG) prequirúrgica con involucro del área motora primaria izquierda y adicionalmente del área motora suplementaria (AMS) ipsilateral. Se realiza resección guiada por EcoG intraoperatoria en AMS izquierda con presencia de estado epiléptico en el posquirúrgico y necesidad de segunda intervención con ampliación de resección sobre el área motora. **Conclusiones**: Posterior a la segunda cirugía resectiva, la paciente presentó una hemiparesia transitoria con una evolución neurológica favorable mediante un programa intensivo de rehabilitación física. Actualmente la paciente permanece libre de crisis. La resección realizada permitió alcanzar un control completo de la epilepsia a 6 meses, sin provocar déficits neurológicos permanentes. Continúa bajo tratamiento anticrisis y terapia física con una notable mejoría en su calidad de vida.

**Palabras Clave:**

epilepsia farmacorresistente; displasia cortical focal; área motora izquierda; cirugía resectiva; paresia de Todd; Test de Wada; electrocorticografia

**Estado Epiléptico Como Manifestación Clínica de Fístula Arteriovenosa Pial Occipital, Reporte de un Caso y Revisión de la Literatura**

Sánchez Encarnación Rocío^1^, Munive Báez Leticia^1^, Melo Guzmán Gustavo^2^

^1^Instituto Nacional de Pediatría (INP), 04530 Ciudad de México, México

^2^Hospital Juárez de México, 07760 Ciudad de México, México

**Introducción**: Las fístulas arteriovenosas piales (pAVFs) o fístulas arteriovenosas no galénicas, son malformaciones vasculares cerebrales poco frecuentes, se caracterizan por una conexión directa de alto flujo entre una arteria pial y una vena cortical, sin un nido capilar intermedio, con mayor prevalencia en pacientes pediátricos, en su mayoría congénitas. Presentan síntomas como insuficiencia cardíaca en neonatos, hidrocefalia, retraso del neurodesarrollo, crisis epilépticas y/o hemorragia intracraneal en lactantes. Debido a su baja prevalencia (1,6%–3,7% de todas las malformaciones vasculares cerebrales) el diagnóstico y tratamiento siguen siendo un reto. **Objetivo**: Sensibilizar la evaluación de manifestaciones epilépticas en pacientes con pAVFs. Estudio descriptivo, observacional, longitudinal y ambispectivo. Reporte de caso clínico. **Resumen Clínico del Caso Con Resultado de Estudios, Tratamiento Instaurado y Evolución**: Femenino de 8 meses, perinatales sin alteraciones, al nacimiento proptosis bilateral, retraso global del neurodesarrollo, síndrome hipotónico central, síndrome piramidal. A los 7 meses presenta dos estados epilépticos focales ingresando al Instituto Nacional de Pediatría, se realizó EEG: actividad epileptiforme en región frontal y temporal derecha, RM cerebral: malformación vascular con fístula arteriovenosa de la ACM izquierda, panangiografía: fístula arteriovenosa pial gigante y aneurisma gigante asociado, con drenaje a seno transverso. Se establece tratamiento interdisciplinario con el equipo de cirugía endovascular del Hospital Juárez de México, realizan embolización de pedículos arteriales provenientes de la ACM izquierda con técnica de Plug and Plush (embolización con coil seguido de embolización con EVOH), además de tratamiento con levetiracetam y oxcarbazepina, logrando control de crisis clínicas, mejoría del alerta y tono axial. **Conclusiones**: La epilepsia es una manifestación subdiagnosticada en pacientes con pAVFs, al ser más evidentes las manifestaciones hemodinámicas. El diagnóstico integral permite establecer alternativas terapéuticas oportunas que detengan la hipoperfusión cerebral, evitando progresión del compromiso sensoriomotor, de la epilepsia o complicaciones como EVC y estado epiléptico. El método y planeación para realizar las sesiones de embolización, debe de ser en forma individualizada, considerando las características anatómicas y hemodinámicas en pacientes pediátricos.

**Palabras Clave:**

epilepsia; fístula arteriovenosa pial; fístula arteriovenosa no Galénica

**Espectro de Pacientes Diagnosticados con Mogad en la Edad Pediátrica en un Hospital de Tercer Nivel: Serie de Casos**

González González Raúl Humberto^1^, Márquez Villaseñor Lenin Antonio^1^, Hernandez González Claudia Nelly^1^, Flores Alfaro Fernanda^1^, Saldivar Dávila Sergio^1^, Vázquez Fuentes Salvador^1^, Chávez Luevanos Beatriz Eugenia^1^

^1^Servicio de Neurología, Hospital Universitario “Dr. José Eleuterio González”, Universidad Autónoma de Nuevo León, 64460 Monterrey, México

**Introducción**: La enfermedad por anticuerpos antiglicoproteína oligodendrocítica de la mielina (MOGAD) es una patología poco común con una base inflamatoria desmielinizante del sistema nervioso central presentando una incidencia de alrededor de 1,6 casos por cada millón de personas por año y llegando a ser hasta 3,1 en la población pediátrica, con una distribución similar entre hombres y mujeres. Esta entidad aparece con un amplio espectro de presentaciones, siendo las más comunes: neuritis óptica, encefalomielitis diseminada aguda o enfermedad multifocal del sistema nervioso central y mielitis transversa, y es monofásica en alrededor del 50% de los casos. Generalmente presentan hallazgos imagenológicos de lesiones hiperintensas en secuencia T2 mal definidas en RMN de cerebro, médula y órbita. Típicamente los hallazgos al estudiar el líquido cefalorraquídeo no demuestran alguna alteración importante. **Objetivo**: Describir el fenotipo clínico, hallazgos imagenológicos y resultados de LCR de los pacientes pediátricos diagnosticados con MOGAD en el periodo de enero 2023 a marzo 2025. **Resumen Clínico del Caso Con Resultado de Estudios, Tratamiento Instaurado y Evolución**: Se estudiaron cuatro casos diagnosticados en el periodo descrito sus características están descritas en la. Dos pacientes con neuritis óptica bilateral y dos con enfermedad multifocal del SNC, edades entre 7 y 14 años, hombres y mujeres por igual. En los 4 casos pudimos observar las hiperintensidades en regiones afectadas al observar la RMN y al momento de estudiar el líquido cefalorraquídeo no mostró alguna alteración en el mismo en los parámetros de: celularidad, glucorraquia, presión de apertura, proteinorraquia. **Conclusiones**: En nuestra serie de casos se observan dos de los fenotipos clínicos más comunes de la presentación de MOGAD en la población pediátrica y se observan los hallazgos imagenológicos y de líquido cefalorraquídeo acordes a lo descrito en la literatura, todos fueron abordados terapéuticamente con esteroides a dosis altas presentando adecuada evolución y egresados para seguimiento externo sin requerir hasta el momento el inicio de terapia de mantenimiento.

**Palabras Clave:**

mogad; desmielinizante; neuritis óptica

**Reporte de Caso de Gangliosidosis Tipo II en un Centro de Alta Especialidad**

Delgado Ramos Claudia Vanessa^1^, Santiago Gómez José Manuel^1^, Rodríguez García Verónica^1^

^1^Centro Médico Nacional 20 de Noviembre, 03104 Ciudad de México, México

**Introducción**: Antecedentes: La gangliosidosis tipo II (GM2), es una enfermedad autosómica recesiva de depósito lisosomal ocasionada por una mutación en el gen *GLB1*, con una incidencia reportada de 1/100.000 a 200.000 nacidos vivos. Clínicamente se caracteriza por ataxia, discapacidad intelectual, alteraciones esqueléticas leves, así como ausencia de hepatoesplenomegalia o de mancha rojo cereza. En algunos casos se ha reportado también la presencia de distonía generalizada. **Objetivo**: Presentación de un caso de gangliosidosis tipo 2 en una paciente adolescente que debutó con presencia de distonía focal. **Resumen Clínico del Caso Con Resultado de Estudios, Tratamiento Instaurado y Evolución**: Femenina de 15 años, debut clínico caracterizado por distonía focal en pie izquierdo que progresó en el transcurso de 10 años a ser generalizada, condicionándole, alteración en la marcha y lenguaje anartrico. Dentro de su abordare se realizó RMN de cerebro encontrándose hiperintensidades T2 y FLAIR en núcleos de la base bilateral, sospechándose enfermedad por depósito. Se solicitó exoma metabólico que reportó la presencia de una variante patogénica en estado heterocigoto en el gen *GLB1*, estableciéndose el diagnóstico de gangliosidosis tipo II. Se determinó un coeficiente intelectual limítrofe por psicometría. Se descartó la presencia de macha rojo cereza en ojo. Se realizó prueba terapéutica con levodopa-carbidopa y trihexifenidilo sin mejoría clínica; presentó respuesta parcial al inicio de baclofeno. **Conclusiones**: La paciente ha tenido una evolución lenta, con mejoría de la calidad de vida con tratamiento farmacológico para distonía. Es pronóstico funcional es malo.

**Palabras Clave:**

gangliosidosis infantiles; enfermedad lisosomal

**Neurofibromas Plexiformes Múltiples Asociados a Neurofibromatosis Tipo 1, Reporte de un Caso**

Ibarra Delgado Alberto^1^, Loman Zúñiga Verónica^1^, Caballero Navarro Yael^1^, Cebada López Flora^1^

^1^UMAE Hospital General, “Dr. Gaudencio González Garza”, 02990 Ciudad de México, México

**Introducción**: La neurofibromatosis tipo 1 es una enfermedad hereditaria asociado una mutación del gen supresor de tumores NF1, clínicamente se manifiesta con seis o más manchas cafés con leche, pecas axilares o inguinales, dos o más neurofibromas, glioma del nervio óptico, dos o más nódulos de Lisch y lesiones óseas. Debido a que el gen *NF1* codifica para la neurofibromina hace que los pacientes con este diagnóstico sean más propensos a crecimientos celulares acelerados con la formación secundaria de neurofibromas; en la población general se estima que estas neoplasias constituyen alrededor del 25–29% de los tumores intradurales extramedulares, sin embargo no se ha explorado lo suficiente, a pesar del impacto significativo que las anomalías espinales pueden tener en la salud mental y física de una persona. **Objetivo**: Describir la presentación clínica de un paciente con lesiones plexiformes a múltiples niveles extramedular. **Resumen Clínico del Caso Con Resultado de Estudios, Tratamiento Instaurado y Evolución**: Se presenta el caso de un paciente masculino de 8 años con diagnóstico de neurofibromatosis desde los 4 años, cuenta con criterios diagnósticos a expensas de neurofibromas plexiformes, familiar de primer grado con neurofibromatosis tipo 1 y múltiples manchas café con leche de mínimo 1 cm de diámetro; 1 año posterior a su diagnóstico inició con pérdida del control de esfínter vesical así como dificultad para la marcha y perdida de la fuerza muscular en extremidades inferiores. Cuenta con resonancia magnética de medula encontrándose con múltiples lesiones plexiformes extramedulares a nivel de T1, T2 y T12. **Conclusiones**: La neurofibromatosis tipo 1 es una patología frecuente en la población, sin embargo, las lesiones localizadas en medula espinal con clínica secundaria ocupan un 5% de todos los neurofibromas que presentan estos pacientes, debido a este es importante un seguimiento estrecho de los pacientes en búsqueda de lesiones a cualquier nivel. Nuestro paciente se podría catalogar como altamente candidato a terapia modificadora de la enfermedad.

**Palabras Clave:**

neurofibroma; neurofibromatosis; medula espinal; compresión

**Lipofuscinosis Ceroidea Neuronal Tipo 6 en Gemelos: Serie de Casos**

Cruz Mejía Saytin Gabriela^1^, Hernández Antúnez Blanca Gloria^1^, Ruiz García Matilde^1^

^1^Instituto Nacional de Pediatría, 04530 Ciudad de México, México

**Introducción**: Las lipofuscinosis ceroideas neuronales se caracterizan por la acumulación de lipopigmentos en los lisosomas neuronales, causando deterioro neurológico progresivo, existen 14 tipos, la tipo 6 (CLN6) es causada por mutaciones en el gen *CLN6*, generalmente inicia entre los 3 y 8 años. Se manifiesta con ataxia, mioclonías, disartria, pérdida visual y regresión de habilidades motoras, con evolución progresiva y tiene un curso fatal. **Objetivo**: Describir las manifestaciones neurológicas asociadas a lipofuncinosis ceroidea neuronal tipo 6. **Resumen Clínico del Caso Con Resultado de Estudios, Tratamiento Instaurado y Evolución**: Caso 1: Masculino producto de embarazo gemelar nace de termino sin complicaciones. A los 2 años de vida presenta ataxia, mioclonías, pérdida progresiva de la visión, y regresión de habilidades como bipedestación, sedestación y dificultad para la deglución. Acude a valoración de 4 años 6 meses se inicia abordaje, RMN cerebral con hiperintensidad difusa de la sustancia blanca cerebral y alteración en el volumen y señal de los tálamos. EEG con actividad paroxística. PEV con onda P100 no definida y dispersa bilateral. Caso 2: Masculino producto de embarazo gemelar nace de termino sin complicaciones. A los 4 años de vida presenta ataxia, pérdida progresiva de la visión, e incapacidad a la bipedestación. Acude a valoración de 4 años 6 meses se inicia abordaje, RMN cerebral con hiperintensidad difusa de la sustancia blanca periventricular, subinsular y de los núcleos dentados. EEG con actividad paroxística en región frontocentral, sin actividad fotoparoxistica. PEV con severa dispersión temporal del componente cortical. Se realiza exoma en ambos pacientes donde se reporta mutación en gen *CLN6* en NM_017882.2: c.200T>C p. (Leu67Pro). **Conclusiones**: El diagnóstico en los síndromes regresivos se dificulta debido a que los signos clínicos suelen ser inespecíficos. La CLN6 es una enfermedad devastadora, su diagnóstico temprano es fundamental para el manejo integral y el asesoramiento genético familiar. Aunque se trata de un trastorno monogénico, la expresión clínica puede variar, incluso entre gemelos, como se reporta en este caso.

**Palabras Clave:**

lipofuscinosis ceroidea neuronal tipo 6; síndrome regresivo; atrofia óptica; ataxia; mioclonías

**Caracterizacion Clinica de una Serie de Pacientes con Distonia Monogenica en el Instituto Nacional de Pediatria**

Sandoval Flores Ivonne^1^, Herrera Mora Patricia^1^, Ruiz García Matilde^1^, Rodríguez Violante Mayela^1^, Arellano Reynoso Alfonso^1^

^1^Instituto Nacional de Pediatría, 04530 Ciudad de México, México

**Introducción**: La distonía provoca posturas y movimientos anormales debido a espasmos musculares esporádicos o continuos. Pueden ser progresivas y deterioran la calidad de vida. La etiología es diversa, el diagnóstico se basa en los síntomas clínicos. Una etiología es la genética y existen formas monogénicas de distonía/discinesia (DYT) como las variaciones patogénicas del gen *VPS16*, *TOR1A*, *THAP1*, *GNAL*, KMT2B y ANO3 y están relacionados a una distonía aislada. **Objetivo**: Describir la evolución clínica de 5 pacientes con distonía de origen monogénico. **Resumen Clínico del Caso Con Resultado de Estudios, Tratamiento Instaurado y Evolución**: Estudio descriptivo, transversal en un periodo de 2019 al 2025, con estudio genético confirmado. Se excluyeron pacientes con otras etiologías. Se detectan 5 pacientes, 3 con mutación del gen *TOR1-A*, 2/3 con inicio focal en extremidades inferiores. Todos presentaron evolución progresiva, generalizada que limitaba la sedestación, bipedestación y marcha, edad promedio de inicio 5 años, 2/3 pacientes cursaron con estado distónico, 2/3 pacientes recibieron marcapaso intracerebral profundo (MICP) con mejoría 1 paciente mioclono-distonía DYT-SGCE, inicio a los 3 años, con contracción sostenida intermitente del pie derecho e hiperextensión rodilla al caminar, caídas frecuentes y mioclonías principalmente en miembros superiores. Torpeza motora fina que limita actividades cotidianas. Sin respuesta a tratamiento farmacológico con colocación (MICP) con mejoría. 1 paciente con distonía KMTB inicio 6 años, con inicio de distonía multifocal a nivel de miembros inferiores con progresión a tronco, extremidades y afección laríngea incapacitante. extremidades y afección laríngea, incapacitante. **Conclusiones**: La distonía relacionada al gen *DYT-TOR1A*, *KMT2B* y *DYT-SGCE* son progresivas e incapacitantes, con pobre respuesta farmacológica, las dos primeras se presentan como distonía aislada. El KMT2B puede afectar la musculatura laríngea y disfunción bulbar, se asocia a discapacidad intelectual. La DYT-SGCE se acompaña de mioclonías a la acción y se asocia a trastornos psiquiátricos. Todos los casos fueron refractarios a tratamiento farmacológico. En esta serie 3/5 pacientes son portadores de MICP con mejoría habilidades motoras. El cuadro clínico y evolución es similar a lo reportado en la literatura internacional.

**Palabras Clave:**

distonía; marcapasos cerebral profundo; discinesia; monogénico; mioclonías

**No Todo es Encefalopatía Hiperbilirrubinémica Neonatal: Síndrome de NESCAV con Variante Patogénica en Gen *KIF1A*. Reporte de Caso**

Sergio Ugarte-Anchondo^1^, Antonio Bravo-Oro^1^, Jorge Luis García-Ramírez^1^

^1^Hospital Regional de Alta Especialidad “Dr. Ignacio Morones Prieto”, 78210 San Luis Potosí, México

**Introducción**: El síndrome NESCAV es un trastorno neurodegenerativo raro, causado por variantes patogénicas en el gen *KIF1A*. Se caracteriza por un retraso global del desarrollo, espasticidad progresiva predominantemente en extremidades inferiores lo que frecuentemente compromete la marcha independiente, y deterioro intelectual de grado variable. Otros hallazgos incluyen retraso del lenguaje, dificultades de aprendizaje, alteraciones conductuales, atrofia óptica con afectación visual cortical, neuropatía periférica, convulsiones, disautonomía, ataxia y distonía. La neuroimagen suele mostrar atrofia cerebelosa y adelgazamiento del cuerpo calloso. Algunos pacientes pueden experimentar regresión del desarrollo, especialmente en habilidades motoras. El fenotipo clínico es ampliamente variable, lo que puede dificultar su diagnóstico. **Objetivo**: Describir la presentación clínica del síndrome NESCAV y destacar la importancia de considerar enfermedades neurodegenerativas genéticas incluso en presencia de antecedentes postnatales sugestivos de insulto cerebral. **Resumen Clínico del Caso Con Resultado de Estudios, Tratamiento Instaurado y Evolución**: Paciente masculino de 9 años con antecedente de hiperbilirrubinemia neonatal multifactorial que requirió hospitalización. Durante el seguimiento se identificó retraso global del desarrollo y retraso motor evidente, por lo que se sospechó daño neurológico secundario a encefalopatía por bilirrubina. No obstante, la evolución clínica mostró espasticidad progresiva, alteraciones en el lenguaje y dificultades para la marcha, lo que motivó una reevaluación diagnóstica por sospecha de un trastorno neurodegenerativo. **Resultados**: La resonancia magnética reveló atrofia cerebelosa sin compromiso de ganglios basales. El electroencefalograma mostró actividad epileptiforme, y el estudio genético confirmó la presencia de una variante patogénica en KIF1A, estableciendo el diagnóstico de síndrome NESCAV. **Conclusiones**: Este caso resalta la importancia de mantener una sospecha clínica amplia frente a cuadros de neurodesarrollo alterado progresivo, incluso en pacientes con antecedentes postnatales potencialmente explicativos. La progresión de síntomas, junto con hallazgos específicos en neuroimagen, deben orientar hacia trastornos neurodegenerativos de base genética. La secuenciación molecular, particularmente del gen *KIF1A*, resulta fundamental para el diagnóstico, pronóstico y asesoramiento genético familiar.

**Palabras Clave:**

KIF1A; NESCAV; trastorno neurodegenerativo; atrofia cerebelosa; retraso del desarrollo

**Trastorno Neurológico Funcional, un Reto Diagnostico, Serie de Casos en un Hospital del Sureste de México**

Marrufo Suarez Cristian A^1^, González Medina Mario^1^, Valladares Sánchez Pablo^1^

^1^Hospital de Alta Especialidad del Niño Dr. Rodolfo Nieto Padrón, 86030, Villahermosa, México

**Introducción**: El trastorno de síntomas neurológicos funcionales es una condición psiquiátrica caracterizada por síntomas que afectan la función sensorial o motora. Estos signos y manifestaciones clínicas no siguen patrones compatibles con enfermedades neurológicas conocidas ni con otras afecciones médicas. Aunque este trastorno carece de una base orgánica demostrable, sus síntomas impactan significativamente la capacidad funcional del paciente. Es importante destacar que dichos síntomas no pueden ser controlados voluntariamente y no se consideran simulados de manera intencional. El término trastorno de conversión fue introducido por primera vez en la literatura por Sigmund Freud. El neurólogo austriaco y fundador del psicoanálisis sostenía que los síntomas funcionales, cuando no podían ser explicados por una enfermedad neurológica o una afección médica subyacente, reflejaban un conflicto inconsciente. **Objetivo**: Presentar una serie de casos de trastorno neurológico funcional en un cuadro comparativo y analizarlos en relación con la bibliografía existente. **Resumen Clínico del Caso Con Resultado de Estudios, Tratamiento Instaurado y Evolución**: Estudio descriptivo en la cual se realizó un análisis de siete casos diagnosticados con trastorno neurológico funcional, con especial énfasis en los antecedentes de relevancia, factores de riesgo, sintomatología neurológica observada y manifestaciones psiquiátricas asociadas. **Conclusiones**: Los hallazgos obtenidos son consistentes con la literatura existente, evidenciando un predominio de cefalea sin las características típicas de la migraña, así como alteraciones motoras, principalmente trastornos de la marcha y parestesias, además de síntomas sensoriales con diversas formas de presentación. Se observó una recuperación completa en todos los casos, sin secuelas a largo plazo.

**Palabras Clave:**

trastornos neurológicos funcionales (TNF); trastornos del movimiento funcionales; crisis convulsivas no epilépticas; neuropsiquiatría; trastorno de conversión

**Presentación de Caso Clinico de Paciente Pediátrico con Sindrome de Schimmelpenning-Feuerstein-Mims y Mutacion en el Gen *SLC12A5***

Daniela Benitez Mexia^1^, Alejandro Durán De La Re^1^, Giussepe Domenico Perez Moya^1^

^1^Hospital Infantil del Estado de Sonora, 83100 Hermosillo, México

**Introducción**: El síndrome de Schimmelpenning-Feuerstein-Mims es un defecto multisistémico raro, neurocutáneo, incidencia estimada de 1 a 3 por cada 10.000 nacidos vivos. La triada clásica de este síndrome es la presencia de nevo sebáceo, convulsiones y retraso mental. La proteína codificada por el gen *SCL12A5* es un cotransportador K-Cl de membrana integral, esta proteína puede actuar con otros cotransportadores de K-Cl, para mantener la homeostasis del cloruro en las neuronas. La epilepsia de la infancia con convulsiones focales migratorias (SLC12A5-EIMFS) se caracteriza por una encefalopatía epiléptica grave de inicio temprano, se han reportado nueve casos de niños con niños con SLC12A5-EIMFS. Es por esta razón que se desea dar a conocer el reporte de caso. **Objetivo**: Describir un caso de Síndrome de Schimmelpenning-Feuerstein-Mims y Epilepsia con mutación en el gen *SLC12A5*. **Resumen Clínico del Caso Con Resultado de Estudios, Tratamiento Instaurado y Evolución**: Masculino lactante de 2 años con diagnósticos comentados. Presentó espasmos infantiles que remitieron el mismo primer año de vida posterior a inicio de tratamiento. Biopsia de lesiones, reporte microscópico hiperqueratosis y paraqueratosis y papilomatosis. Resonancia magnética de cerebro 2024, reportado asimetría de lóbulos temporales, mayor volumen en lóbulo temporal derecho, sugestivo de encephalitis. Electroencefalograma actividad epileptiforme con patrón de brote supresión. Electroencefalograma disfuncional por asimetría y asincronía interhemisférica con la presencia de ondas agudas y trifásicas frontotemporales derechas. Actualmente presenta mioclonías palpebrales en hemicara derecha acompañado de alteración en el estado de alerta. Tratamiento actual Vigabatrina y Valproato de Magnesio. Presenta nevo epidérmico verrugoso, afecta piel cabelluda y hemicara derecha y cuello porción media anterior. Presencia de alopecia en zonas parietal derecho y parietooccipital derecho. **Conclusiones**: Se hace diagnóstico de Síndrome de Schimmelpenning-Feuerstein-Mims por clínica (tríada clásica), se encuentra mutación en gen *SLC12A5*, el cual cuenta con significancia clínica para trastornos epilépticos y neurológicos que clásicamente se describen como crisis focales migratorias, nuestro en paciente se mostraron en su primer año de vida espasmos infantiles, actualmente presenta mioclonías palpebrales derechas. Continúa en seguimiento con neurología, dermatología y genética.

**Palabras Clave:**

Sindrome De Schimmelpenning-Feuerstein-Mims; epilepsia; gen *SLC12A5*

**Enfermedad de Lafora, Reto Diagnóstico: Reporte de Caso**

Sánchez Vargas Jorge Luis^1^, Ruiz García Matilde^1^, López Corella^2^, Magos Rodríguez Daniel^1^

^1^Servicio de Neurología Instituto Nacional de Pediatría (INP), 04530 Ciudad de México, México

^2^Servicio de Patología Instituto Nacional de Pediatría (INP), 04530 Ciudad de México, México

**Introducción**: La enfermedad de Lafora (EL) es una epilepsia mioclónica progresiva autosómica recesiva, mortal y de inicio en la adolescencia, caracterizada por la acumulación anormal de glucógeno en forma de cuerpos de Lafora en neuronas y células gliales. Se asocia con deterioro cognitivo, mioclonías intratables y convulsiones de difícil control. Las mutaciones en los genes *EPM2A* y *NHLRC1* son responsables de esta condición. Su diagnóstico es complejo debido a la superposición clínica con otras epilepsias progresivas. **Objetivo**: Describir las manifestaciones clínicas, evolución y hallazgos diagnósticos de un caso de enfermedad de Lafora, resaltando la importancia del diagnóstico temprano en pacientes adolescentes con epilepsia mioclónica progresiva. **Resumen Clínico del Caso Con Resultado de Estudios, Tratamiento Instaurado y Evolución**: Material y Métodos: Se presenta el caso clínico de una paciente femenina de 16 años, sin antecedentes perinatales relevantes, con inicio de crisis tónico-clónicas a los 11 años. Se analizaron datos clínicos, neurológicos, neuroimagen, EEG y biopsia de piel. Se documentó el tratamiento farmacológico previo y la evolución clínica. **Resultados**: La paciente presentó mioclonías diarias, ataxia progresiva, disartria y deterioro cognitivo severo. El EEG mostró descargas epileptiformes occipitales; la resonancia magnética reveló atrofia cerebelosa y la biopsia cutánea confirmó cuerpos de Lafora. Pese a múltiples esquemas antiepilépticos, la paciente evolucionó a dependencia funcional con disfagia y pérdida de autonomía motora. **Conclusiones**: La EL debe considerarse en adolescentes con epilepsia mioclónica progresiva y deterioro neurológico. La participación de astrocitos en la fisiopatología y la correlación genotipo-fenotipo refuerzan la necesidad de un diagnóstico genético temprano. Aunque el tratamiento es sintomático, las terapias génicas y los inhibidores de glucógeno sintasa emergen como alternativas prometedoras.

**Palabras Clave:**

epilepsia mioclónica progresiva; enfermedad de Lafora; crisis epilépticas intratables; neurodegeneración; diagnóstico diferencial

**Estudio Molecular en la Epilepsia Farmacorresistente, Microcefalia y Atrofia Cortical**

Castro Ruiz-Alfredo^1^, Gutiérrez Soto-Marisol^1^, Reyes Cuayahuitl-Araceli^1^, Rayo Mares-Jesús Darío^1^

^1^Servicio de Neurología Pediátrica, Hospital de Pediatría CMN Siglo XXI, IMSS, 06720 Ciudad de México, México

**Introducción**: Los complejos de partículas de proteína de tráfico (TRAPP) regulan el tráfico de vesículas a través de la actividad del factor de intercambio de nucleótidos de guanina. El tráfico mediado por TRAPP es crucial para el desarrollo y la función cerebral, ya que las variantes en múltiples subunidades de TRAPP conducen a trastornos superpuestos del desarrollo neurológico, con trastornos del movimiento, discapacidad intelectual, epilepsia, y disminución del volumen cerebral. **Objetivo**: Describir la presentación clínica, hallazgos electroencefalográficos y de neuroimagen en un paciente con epilepsia y variante patogénica del gen *TRAPPC6B*. **Resumen Clínico del Caso Con Resultado de Estudios, Tratamiento Instaurado y Evolución**: Masculino de 4 años 7 meses, con antecedente de retraso global del neurodesarrollo, microcefalia, ausencia de lenguaje expresivo y epilepsia farmacorresistente. Inicia a los 6 meses, con espasmos epilépticos, a los 2 años inicia con crisis a focales motoras tónicas con alteración del estado de conciencia, se agregan crisis focales motoras mioclónicas de extremidad superior derecha y crisis mioclónico-atónicas, manejado con diferentes fármacos antiepilépticos, en manejo actual con valproato de magnesio, levetiracetam y lamotrigina. Presenta crisis focales tipo tónicas (3/día) y atónicas-mioclónicas (3/día). En la RMN de encéfalo se observa atrofia subcortical frontotemporoparietal izquierda, en el registro electroencefalográfico frecuentes paroxismos de puntas, ondas agudas y complejos de punta onda lenta en región frontotemporal bilateral, temporoparietal izquierdo, temporal derecho como focos independientes con propagación a áreas adyacentes y generalización secundaria en vigilia y sueño. Con exoma que reporta variante heterocigota del gen *TRAPPC6B*. **Conclusiones**: Los fenotipos asociados a TRAPPC6B se superponen con variantes en genes que codifican otras subunidades. La epilepsia asociada a variantes en el gen *TRAPPC6B* se clasifica según la ILAE como una epilepsia de inicio temprano dentro de un trastorno neurodesarrollo. El paciente presentado cuenta con las características fenotípicas descritas, así como la identificación de la variante patogénica genética. El pronóstico se considera malo, se tienen datos de resistencia al tratamiento en un 30–50% de los casos, retraso cognitivo y motor en un 60–80% y atrofia cerebral progresiva en un 50–70%.

**Palabras Clave:**

TRAPPC6B; epilepsia farmacorresistente; atrofia cerebral

**Manifestaciones Neurológicas Asociadas a Mycoplasma Pneumoniae: Serie de Casos**

Medellín López Haideé Deyanira^1^, Sánchez Encarnación Roció^1^, Munive Báez Leticia^2^

^1^Instituto Nacional de Pediatría (INP), 04530 Ciudad de México, México

^2^Universidad Nacional Autónoma de México (UNAM), 04510 Ciudad de México, México

**Introducción**: Mycoplasma pneumoniae ha sido subdiagnosticado en pacientes pediátricos inmunocompetentes con encefalitis en ausencia de manifestaciones respiratorias concurrentes; los pacientes pueden presentar un espectro amplio de manifestaciones neurológicas ocasionadas por mecanismos patogénicos directos y por mecanismos autoinmunes secundarios. **Objetivo**: Estudio descriptivo, observacional, retrospectivo y transversal, serie de casos. Describir las manifestaciones neurológicas asociadas a neuroinfección por Mycoplasma pneumoniae y sensibilizar el diagnóstico dirigido aún en ausencias de manifestaciones respiratorias. **Resumen Clínico del Caso Con Resultado de Estudios, Tratamiento Instaurado y Evolución**: Caso 1: Masculino 13 años con incoordinación de la marcha, habla escandida, hipotonía troncal, ataxia, dismetría bilateral, hiperreflexia pendular, antecedente de síntomas respiratorios y gastrointestinales 7 días previos, se realizó punción lumbar obteniendo LCR: microproteínas 43 mg/dL, células 22 (87% mononucleares, 13% polimorfonucleares) y glucosa 55 mg/dL, panel meníngeo negativo, por antecedente infeccioso se toma hisopado nasal con PCR positiva para Mycoplasma pneumoniae, manejo con levofloxacino con mejoría de la sintomatología. Caso 2: Masculino 8 años ingresa con irritabilidad, alteración del alerta, crisis tónico-clónica y signos meníngeos, antecedente 14 días previos de infección de vías aéreas superiores, se realiza punción lumbar obteniendo LCR: microproteínas 45 mg/dL, glucosa 67 mg/dL, células 120 (96% mononucleares, 4% polimorfonucleares), panel meníngeo negativo, hisopado nasal con PCR positiva para Mycoplasma pneumoniae. Se indicó levofloxacino, levetiracetam, durante su evolución presentó síndrome piramidal y extrapiramidal, por lo que se agrega gammaglobulina humana 1g/kg/dosis, corticoesteroide a dosis inmunomoduladora y baclofeno, se realizó RMN cerebral identificando hiperintensidad en T2 FLAIR en núcleos caudados y putamen, con reforzamiento con el material de contraste y disminución de N-Acetil-aspartato en espectroscopía. El paciente recupera alerta, presenta conducta impulsiva, control clínico de las crisis epilépticas, reduce distonía, pero activa movimientos coreicos. **Conclusiones**: Un panel meníngeo negativo no descarta la presencia de neuroinfección ante las manifestaciones clínicas, es importante capacitar al personal en la realización de PCR en LCR para Mycoplasma. Las manifestaciones clínicas por mecanismos directos parecen ser más sensibles al tratamiento específico, mientras que la activación de mecanismos autoinmunes secundarios se asocia más a secuelas, siendo importante controlar desde el inicio la respuesta inmunológica del paciente.

**Palabras Clave:**

Mycoplasma pneumoniae; cerebelitis; vasculitis; núcleos de la base

**Uso de Taurina Como Tratamiento Preventivo de “Stroke Like” en Pacientes con Diagnóstico de Síndrome de Melas, Reporte de 3 Casos**

Ibarra Delgado Alberto^1^, Caballero Navarro Yael^1^, Rosas Contreras Miguel Ángel^1^, Loman Zúñiga Verónica^1^, Cebada López Flora^1^

^1^UMAE Hospital General, “Dr. Gaudencio González Garza”, 02990 Ciudad de México, México

**Introducción**: El síndrome de MELAS (encefalomiopatía mitocondrial, acidosis láctica y episodios similares a evento vascular cerebral isquémico, por sus siglas en inglés) es una mitocondropatía hereditaria, se manifiesta por episodios similares a eventos vasculares cerebrales “stroke like” antes de los 40 años, encefalopatía y acidosis láctica. El tratamiento recomendado incluye suplementación con cofactores y antioxidantes, actualmente se ha propuesto el uso de taurina como tratamiento preventivo de “stroke like”, sin embargo, en la población pediátrica aun sin establecer una dosis especifica. **Objetivo**: Comparar la utilidad de la taurina como tratamiento preventivo de eventos de “stroke like” en pacientes con síndrome de MELAS. **Resumen Clínico del Caso Con Resultado de Estudios, Tratamiento Instaurado y Evolución**: Caso 1: Femenino de 15 años con diagnóstico de síndrome de MELAS en 2019, presentó 4 eventos de “stroke like” previo al inicio de taurina, actualmente en manejo de 3 gramos/día (0,18 mg/kg/día), posterior al tratamiento solo ha presenta 1 nuevo evento. Caso 2: Femenino de 12 años con diagnostico a los 8 años en 2019. Presentó 5 eventos previo al inicio de taurina, está iniciándose en 2023, posteriormente se mantuvo libre de eventos durante un año, en 2024 presentó 3 eventos requiriendo aumento en la dosis de taurina, y en 2025 ha presentado 1 evento con dosis de taurina en 6,6 gramos/día (0,26 g/kg/día). Caso 3: Masculino de 13 años, diagnostico a los 10 años en 2022, presentó 5 eventos previo al inicio de taurina, posteriormente 1 año libre de “stroke like” y hasta la fecha ha presentado 3 eventos más, actualmente en manejo con taurina 2 gramos/día (0,05 g/kg/día). **Conclusiones**: Se pudo observar una disminución de los eventos de “stroke like” posterior al inicio de taurina, de acuerdo con lo observado se podría considerar que el uso de taurina ayuda como tratamiento preventivo de dichos eventos, de igual manera posterior a una recaída al incrementar la dosis de taurina se observó una remisión más rápida de los síntomas, sin embargo, se requiere de estudios más amplios para determinar su eficacia.

**Palabras Clave:**

MELAS; taurina; preventivo; stroke like

**Enfermedad de Huntington de Inicio Juvenil: Presentación de un Caso Clínico con Alteraciones Neuropsiquiátricas y Confirmación Genética**

Joel Iván Arriaga-Espinosa^1^, Araceli Reyes-Cuayahuitl^1^, Luis Antonio Arenas-Aguayo^1^, Juan Carlos Huicochea Montiel^1^

^1^Hospital de Pediatría CMN Siglo XXI, 06720 Ciudad de México, México

**Introducción**: La enfermedad de Huntington pediátrica o enfermedad de Huntington de inicio juvenil (EHJ), es una forma rara y temprana de la enfermedad de Huntington, trastorno neurodegenerativo autosómico dominante causado por una expansión anormal del triplete CAG en el gen *HTT* (5% de los casos). Se caracteriza por una tríada de síntomas motores, cognitivos y psiquiátricos. A diferencia de la forma adulta, la JHD suele presentar distonía, bradicinesia y temblores, mientras que los movimientos coreicos son menos comunes, además de crisis epilépticas. La progresión de la enfermedad es más rápida en comparación con adultos. **Objetivo**: Descripción clínica de un paciente con diagnóstico clínico de EHJ. **Resumen Clínico del Caso Con Resultado de Estudios, Tratamiento Instaurado y Evolución**: Femenino de 11 años, con antecedente de madre y abuelo materno de enfermedad de Huntington (finados) y sospecha de episodio depresivo. Se reportan alteraciones del movimiento, sueño fraccionado, dificultades en el aprendizaje, principalmente en la lectoescritura. Cuenta con registro electroencefalográfico con actividad paroxística generalizada con complejos punta-onda lenta y polipunta-onda lenta durante el sueño sin evidencia clínica. Se inicio tratamiento con levetiracetam. La resonancia magnética muestra atrofia de ambos núcleos caudados y lenticulares. Se agrega a la evolución dificultad para control de esfínteres y alteraciones de la deglución. Se realiza estudio molecular detectando alelo con 75 repeticiones del triplete CAG (penetrancia completa) confirmando el diagnóstico de EHJ. **Conclusiones**: La enfermedad de Huntington de inicio pediátrico, aunque poco frecuente, debe considerarse en pacientes con antecedentes familiares relevantes y síntomas neurológicos, psiquiátricos y cognitivos progresivos. El caso descrito destaca la importancia de una evaluación integral, que incluya estudios de neuroimagen, electroencefalograma y confirmación genética, permitiendo un diagnóstico certero en etapas tempranas. El abordaje multidisciplinario y el seguimiento continuo son fundamentales para mejorar la calidad de vida del paciente y ofrecer asesoramiento genético oportuno a la familia.

**Palabras Clave:**

Huntington; pediatria; neuropsiquiatria

**Meningitis por Tuberculosis. Presentación de Dos Casos**

Flores Sánchez Zyanya Stephanie^1^, Mario González Medina^1^, Valladares Sánchez Pablo^1^

^1^Hospital de Alta Especialidad del Niño “Rodolfo Nieto Padrón”, 86030 Villahermosa, México

**Introducción**: La meningitis por tuberculosis (MT) es una enfermedad endémica en el mundo, poco frecuente en pediatría, causada por Mycobacterium tuberculosis. Su curso suele ser grave y su diagnóstico definitivo representa un desafío clínico. Afecta principalmente a personas inmunocomprometidas. **Objetivo**: Mostrar las características clínicas, de laboratorio, gabinete, evolución y tratamiento de dos pacientes con MT. **Resumen Clínico del Caso Con Resultado de Estudios, Tratamiento Instaurado y Evolución**: Caso de dos pacientes con MT, con edades entre 12 meses y 5 años, género femenino y masculino, ninguno recibió la vacuna BCG, ambos sin antecedentes de inmunocompromiso. Durante la fase prodrómica presentaron fiebre y disminución del peso corporal. El paciente masculino cursó con tuberculosis pulmonar, alteración del estado de alerta, estado epiléptico, signos meníngeos, cuadriparesia espástica, hipertensión endocraneana, distonía y síndrome neuroendocrino; posteriormente, afección del nervio craneal III. La niña presentó fiebre y pubertad precoz, un mes después, crisis convulsivas, cráneo hipertensivo, signos meníngeos, cuadriparesia espástica, mínimo estado de conciencia, coma y finalmente afección de pares craneales. Ambos pacientes presentaron líquido cefalorraquídeo (LCR) y tomografía de cráneo con hallazgos característicos de infección por tuberculosis, encefalopatía en el EEG, y solo uno de ellos con BAAR y GeneXpert® positivos (niño). Solo uno recibió tratamiento antifímico, los dos presentaron el mismo desenlace, fallecimiento. **Conclusiones**: La meningitis tuberculosa en los pacientes presentados demuestra que esta patología puede producir secuelas graves, incluso, desencadenar la muerte, como lo menciona. Entre los síntomas prodrómicos se encontraron fiebre persistente, cefalea y pérdida de peso. En la fase meníngea, los más comunes fueron crisis convulsivas, alteración del estado de alerta, signos meníngeos, síndrome de motoneurona superior, hipertensión endocraneana y compromiso de nervios craneales, siendo el nervio craneal III el más afectado. Ninguno de los dos pacientes recibió la vacuna BCG. Estudios como los de han demostrado que la vacuna BCG reduce en un 73% el riesgo de meningitis tuberculosa, por lo tanto, es indispensable su aplicación.

**Palabras Clave:**

meningitis por tuberculosis; Mycobacterium tuberculosis; vacuna BCG

**Síndrome del Desarrollo Multisistémico en la Edad Pediátrica. Reporte de un Caso**

Marquez Villaseñor Lennin Antonio^1^, Falcon Delgado Alfredo^1^, Cantú Salinas Adriana Carlota^1^, De La Garza Pineda Oscar^1^, Villarreal Rodríguez Diana Laura^1^, Vázquez Fuentes Salvador^1^, Carrión García Ana Luisa^1^, Chávez Luevanos Beatriz Eugenia^1^

^1^Servicio de Neurología, Hospital Universitario, Dr. José Eleuterio González, Universidad Autónoma de Nuevo León, 64460 Monterrey, México

**Introducción**: El síndrome del desarrollo multisistémico o síndrome de KBG (SKBG) es una enfermedad genética autosómica dominante originada por variantes patogénicas de la proteína 11 del dominio anquirina (ANKRD11) en el cromosoma 16q24.3. Esta proteína influye en la expresión de varios genes vinculados al crecimiento embrionario, neuronal y óseo. El SKBG puede manifestarse con malformaciones cerebrales, retraso en el neurodesarrollo, macrodoncia, anomalías esqueléticas y alteraciones craneofaciales distintivas. Según las estadísticas internacionales, actualmente se han identificado 200 pacientes con el SKBG a nivel mundial. **Objetivo**: Investigar las características clínicas, genéticas y moleculares del SKBG con la finalidad de un diagnóstico temprano, así como entender su variabilidad clínica y su impacto en la calidad de vida de los pacientes. **Resumen Clínico del Caso Con Resultado de Estudios, Tratamiento Instaurado y Evolución**: Masculino de 5 años con las siguientes manifestaciones clínicas: Frente amplia, hipoplasia de tercio medio facial, puente nasal prominente, hipertelorismo, orejas de implantación baja, labios delgados, macrodoncia, braquidactilia, clinodactilia del 5to dedo bilateral. Actualmente cursando con retraso global del neurodesarrollo por test de Denver II en las áreas motor grueso, motor fino, social y lenguaje. Escala de maduración de Vineland reportando madurez cerebral con retraso de 18 meses, así como trastorno por déficit de atención e hiperactividad (TDAH). Resultados de estudios de abordaje: Resonancia magnética simple y contrastada de cerebro con hallazgos de inserción baja del tentorio, aumento de amplitud de cisterna magna, hipertelorismo y quiste aracnoideo temporal derecho sin evidencia de realces a la administración de gadolinio. Panel genético para trastornos neurodegenerativos de 241 genes con reporte de una variante patogénica (c.3240del-p. Asp1081Thrfs*237) identificada en el gen *ANKRD11* asociado con SKBG. **Conclusiones**: El SKBG es una condición genética inusual que afecta el desarrollo cognitivo, físico y neurológico. A pesar de su baja prevalencia, su estudio continuo es esencial para avanzar en el entendimiento de sus características, lo que permitirá mejorar el diagnóstico, manejo y calidad de vida de los pacientes.

**Palabras Clave:**

síndrome KBG (SKBG); proteína; ANKRD11; anquirina; enfermedad genética; autosómica dominante; macrodoncia

**Empiema Subdural VS Absceso Epidural en Pediatría: Dos Escenarios Clínicos Derivados de una Sinusitis Complicada**

Jacqueline D. Lucio-Escamilla^1^, Cantú Salinas Adriana Carlota^1^, Carrión García Ana Luisa^1^, Vázquez Fuentes Salvador^1^, De la Garza Pineda Oscar^1^, Chávez Luévanos Beatriz Eugenia^1^

^1^Servicio de Neurología, Hospital Universitario “Dr. José Eleuterio González”, Universidad Autónoma de Nuevo León, 64460 Monterrey, México

**Introducción**: El empiema subdural y el absceso epidural son complicaciones neurológicas infrecuentes pero graves en pediatría, asociadas comúnmente a sinusitis. El empiema subdural se localiza entre la duramadre y la aracnoides y puede extenderse ampliamente por la convexidad cerebral, mientras que el absceso epidural, ubicado entre el cráneo y la duramadre, puede originarse por diseminación desde estructuras vecinas como la celulitis orbitaria o el absceso subperióstico. Dada su elevada morbimortalidad, el diagnóstico temprano mediante imagenología y el tratamiento oportuno con antibióticos y drenaje quirúrgico son fundamentales. **Objetivo**: Comparar las manifestaciones clínicas, diagnósticas y terapéuticas del empiema subdural y el absceso epidural en pacientes pediátricos con sinusitis complicada, resaltando la importancia de la identificación temprana para prevenir secuelas neurológicas. **Resumen Clínico del Caso Con Resultado de Estudios, Tratamiento Instaurado y Evolución**: Caso 1: Femenina de 13 años con antecedente de sinusitis recurrente parcialmente tratada, que inició con fiebre y cefalea frontotemporal punzante. Posteriormente desarrolló debilidad progresiva en hemicuerpo izquierdo, con hemiparesia 2/5 e hiporreflexia, sin signos meníngeos ni alteraciones en LCR. La resonancia magnética evidenció empiema subdural frontoparietal derecho con extensión interhemisférica, paquimeningitis, leptomeningitis y pansinusitis derecha. Recibió ceftriaxona, metronidazol y vancomicina intravenosos, con recuperación neurológica progresiva. Caso 2: Masculino de 9 años, con antecedente de sinusitis recurrente, inició con cefalea frontal de tipo opresiva de intensidad 10/10 en la escala visual análoga, exacerbada por maniobra de Valsalva y edema bipalpebral derecho. Fue diagnosticado con celulitis orbitaria y absceso subperióstico, y tratado con ceftriaxona, vancomicina y levofloxacino. La tomografía reveló absceso epidural frontal derecho y pansinusitis. Neurológicamente sólo presentó signos piramidales izquierdos, sin otras alteraciones. **Conclusiones**: La sinusitis complicada constituye un factor predisponente para el desarrollo de entidades neurológicas severas en la población pediátrica, como el empiema subdural, caracterizado por focalidad neurológica, y el absceso epidural, de presentación clínica más insidiosa. Su identificación oportuna es crucial para prevenir secuelas neurológicas permanentes y disminuir la mortalidad asociada.

**Palabras Clave:**

empiema subdural; absceso epidural; sinusitis complicada; complicaciones intracraneales

**Encefalitis Autoinmune Asociada a Proteína 6 Dipeptidil Positiva: Un Caso Clínico Pediátrico Inusual**

Soto Macedo Andrea Sofia^1^, Vazquez Briseño J Jesús^1^, Martínez Guzman Edgar^1^

^1^Hospital Regional ISSSTE León, 37520 León de los Aldama, México

**Introducción**: La encefalitis anti-proteína similar a dipeptidil-peptidasa 6 (DPPX) es una forma rara de encefalitis autoinmunitaria. **Objetivo**: Identificar de manera oportuna dado a que la ausencia de hallazgos típicos de imagen y neurofisiológicos dificulta el diagnóstico temprano. **Resumen Clínico del Caso Con Resultado de Estudios, Tratamiento Instaurado y Evolución**: Paciente de 4 años que presenta inicio abrupto de síntomas, comenzando con indiferencia al medio, mirada perdida, sin respuesta a estímulos y 10 episodios de vómito. Acude a urgencias, donde presenta crisis epilépticas tónicas, clónicas y generalizadas. Se observa regresión del neurodesarrollo, por lo que se decide intubación. La evolución es tórpida y en la RMN de cráneo, se detecta hiperintensidad hipocampal bilateral. Se solicita muestra de LCR, con resultado positivo para anticuerpos anti-proteína 6-dipeptidil, confirmando encefalitis autoinmune. Se inicia manejo con inmunoglobulina intravenosa. **Conclusiones**: Esta encefalitis, causa disfunción cognitiva, síntomas neuropsiquiátricos e hiperexcitabilidad del sistema nervioso central. Su expresión en el hipocampo, cerebelo, estriado, riñón y plexo mientérico explica las características clínicas multifocales de la autoinmunidad contra DPPX. El diagnóstico de encefalitis anti-DPPX se basa en la presencia de anticuerpos contra DPPX en LCR. Los subtipos de encefalitis autoinmunitaria mediada por anticuerpos contra GABA-B, AMPAR, GABA-A, DPPX estas representan causas poco comunes de encefalitis mediada por anticuerpos, por lo que se beneficiarán de un mayor reconocimiento y una disponibilidad más amplia en las pruebas de anticuerpos.

**Palabras Clave:**

DPPX, dipeptidil-peptidasa 6; RMN, resonancia magnética; LCR, líquido cefalorraquídeo

**Neuropatía Craneal Múltiple Autoinmune en Paciente Pediátrico, un Desafío Diagnóstico. Reporte de un Caso**

Katia Moreno-Silva^1^, Martin Arturo Silva-Ramírez^1^, Flora Cebada López^1^, Yael Caballero-Navarro^1^, Fabiola Marycruz De la Fuente-Silva^1^, Edwin Steven Vargas-Cañas^1^

^1^Instituto Mexicano del Seguro Social UMAE Hospital General” Dr. Gaudencio González Garza”, Centro Médico Nacional La Raza, 02990 Ciudad de México, México

**Introducción**: La neuropatía craneal múltiple se define como una disfunción de dos o más nervios craneales la cual puede ocurrir de manera simultánea o secuencialmente. La afectación puede ocurrir en cualquier punto de su recorrido, desde el tronco encefálico hasta su trayecto periférico. Su etiología puede ser por distintos mecanismos patogénicos como procesos infecciosos, inflamatorios, neoplásicos, vasculares, tóxicos, traumáticos, trastornos óseos y autoinmunes. **Objetivo**: Destacar la importancia de un adecuado abordaje diagnostico para considerar la causa autoinmune de neuropatía craneal múltiple en pacientes pediátricos. **Resumen Clínico del Caso Con Resultado de Estudios, Tratamiento Instaurado y Evolución**: Masculino de 12 años con neuropatía craneal múltiple de tres meses de evolución caracterizado por ptosis palpebral izquierda y paresia del III, IV Y VI nervio craneal bilateral. Se realizó prueba de hielo positiva y se inició manejo empírico con piridostigmina sin presentar mejoría clínica. Se realizó protocolo diagnostico para descartar múltiples etiologías, con toma de anticuerpos anti-MUSK negativos y anti-Acetilcolina positivos, por lo que se solicita prueba de estimulación nerviosa repetitiva negativa a fatigabilidad de placa neuromuscular, por lo que se suspende piridostigmina. Posteriormente, dentro del abordaje para causas infecciosas se descartan etiologías virales y micóticas, con punción lumbar con resultado positivo para Borrelia burgdorferi por lo que por parte de infectología se inició doxiciclina. El paciente recibió prednisona oral, pero al suspenderla mostró recaída de la oftalmoplejía, por lo que se realizó biopsia de musculo con resultado negativo para miopatías mitocondriales. Actualmente se sospecha etiología autoinmune y se decide la administración de rituximab para retiro posterior de la prednisona. **Conclusiones**: En el abordaje de neuropatía craneal múltiple se deben descartar causas infecciosas que son las más frecuentes seguidas de las causas autoinmunes. En nuestro caso al retiro del inmunomodulador el paciente presentó deterioro neurológico, por lo que ante resultados negativos de fatigabilidad de la placa neuromuscular y recaída del paciente nos hace sospechar una etiología autoinmune. En el seguimiento del paciente se ha iniciado manejo con Rituximab por lo que estaremos en vigilia de su respuesta terapéutica.

**Palabras Clave:**

oftalmoplejía; neuropatía craneal multiple; autoinmune

**Infarto Talámico Bilateral por Oclusión de la Arteria de Percherón en un Paciente Pediátrico: Reporte de Caso**

Karla Fabiola Díaz Corral^1^, Luz Elena Armejo Chávez^1^, Marysol Ulloa Carrillo^1^, Irving Mendoza^1^

^1^Servicio de Neurología Pediátrica, Centro Médico Nacional de Occidente, 44340 Guadalajara, México

**Introducción**: El tálamo recibe irrigación principalmente a través de ramas del segmento P1 de las arterias cerebrales posteriores. En 1973 el neurólogo francés Gérard Percheron describió tres variantes de irrigación de estas, identificando que en algunos casos una sola rama perforante de la arteria cerebral posterior puede irrigar ambos tálamos (variante presente en 4–12% de la población). La oclusión de esta arteria resulta en un infarto bilateral medial de ambos tálamos, caracterizándose por la triada de compromiso de conciencia, parálisis de la mirada vertical y alteración de la memoria, llevando el nombre de Síndrome de Percherón, con una incidencia reportada en adultos de 0,1 a 0,3% de todos los accidentes cerebrovasculares, y siendo extremadamente rara en población pediátrica. **Objetivo**: Describir las características clínicas, diagnósticas y terapéuticas de un caso de Síndrome de Percherón en edad pediátrica. **Resumen Clínico del Caso Con Resultado de Estudios, Tratamiento Instaurado y Evolución**: Se describe el caso clínico de un paciente pediátrico, atendido en el CMNO del IMSS. La información fue recolectada a partir del expediente clínico físico y electrónico; se analizaron notas médicas, resultados de laboratorio y de imagen, así como valoraciones por los diferentes servicios del hospital que completaron su abordaje diagnóstico. Se trata de paciente femenino de 17 años, sin antecedentes heredofamiliares o perinatales de importancia para su padecimiento, con antecedente de encontrarse en puerperio tardío, además de ser consumidora de alcohol, tabaco, marihuana y cocaína. Inicia con hipertensión, cefalea intensa, mareo, dolor ocular, bradilalia y lenguaje desorganizado. Durante la exploración inicial se muestra con fluctuaciones del estado de alerta, con paresia a la mirada inferior, pupilas con anisocoria. La tomografía y la angioresonancia evidenciaron lesiones compatibles con isquemia talámica bilateral. Abordándose y descartándose patología infecciosa, hematológica, toxicológica y cardíaca. **Conclusiones**: Este caso resalta la importancia de ampliar los diagnósticos diferenciales de pacientes pediátricos, considerando los cambios en los hábitos sociales, culturales y ambientales de la población pediátrica actual.

**Palabras Clave:**

infarto talámico; isquemia cerebral; síndrome de Percherón; evento vascular; pediatrías

**Síndrome de Megdel: Una Enfermedad del Siglo XXI**

Maria Fernanda Pérez-Álvarez^1^, Fabiola Marycruz De la Fuente-Silva^1^, Martín Arturo Silva-Ramírez^1^, Flora Cebada-López^1^

^1^Instituto Mexicano Del Seguro Social, UMAE Hospital General “Dr. Gaudencio González Garza” Centro Médico Nacional La Raza, 02990 Ciudad de México, México

**Introducción**: El Síndrome de MEGDEL se caracteriza por aciduria 3-metilglutacónica, sordera, encefalopatía y síndrome similar a Leigh. Descrito en 2006, descubriendo mutación del gen *SERAC1* en 2012, con herencia autosómica recesiva. Puede presentar alteraciones multiorgánicas, con fenotipo amplio, reportándose aproximadamente 102 casos a nivel mundial. Existen 2 tipos de inicio, juvenil e infantil, siendo esté último más severo. Cursan con elevación de lactato y alanina. En el período neonatal o durante el primer año de vida, alrededor del 50% desarrollan disfunción hepática transitoria grave, y/o signos de insuficiencia hepática. La RM de cráneo presenta afectación por etapas: núcleos basales (signo del ojo putaminal), atrofia cerebral y cerebelosa. El diagnóstico se confirma mediante estudio genético. No existe tratamiento curativo, teniendo un pronóstico sombrío en la mayoría de los casos. **Objetivo**: Presentar un caso de síndrome de MEGDEL, con énfasis en sus características clínicas y la importancia del asesoramiento genético. **Resumen Clínico del Caso Con Resultado de Estudios, Tratamiento Instaurado y Evolución**: Masculino de 6 años, hiperbilirrubinemia e hipoglicemias al nacimiento, hospitalizado la primera semana de vida, con mal manejo de secreciones, succión débil. Desarrollo psicomotor normal, con regresión de habilidades previamente adquiridas a los 9 meses, microcefalia. Desde el primer año con espasticidad, con movimiento coreoatetósicos, a los 3 años se realiza gastrostomía/funduplicatura por alteración en la mecánica de deglución, a los 4 años se detecta atrofia óptica, hipoacusia bilateral y epilepsia generalizada. EEG lento para la edad, paroxismos frecuentes de polipunta y onda lenta frontocentral, frontotemporal derecho, sin grafoelementos de sueño. RM de cráneo con lesiones en núcleos basales, atrofia cortico subcortical e hipoplasia cerebelosa mostrando evolución de lesiones concordantes con diagnóstico. Panel multigen con 2 variantes patogénicas en gen *SERAC1*, asociado con síndrome de MEGDEL. Actualmente persiste con crisis epilépticas, en seguimiento multidisciplinario, con adecuado estado nutricio. **Conclusiones**: El Síndrome de MEGDEL es una entidad poco conocida, descrita recientemente con escasa información a nivel mundial. Se requiere de su difusión y conocimiento, dado el impacto para el paciente y su familia, haciendo hincapié en su clínica e imagen característica y el asesoramiento genético, con el fin de apoyar a las familias de pacientes afectados y/o portadores.

**Palabras Clave:**

MEGDEL; ojo putaminal

**Pseudotumor Cerebri en Población Pediátrica de un Hospital de Tercer Nivel: Serie de Casos**

Karla Fabiola Díaz Corral^1^, Alma Maritza Huerta Hurtado^1^

^1^Centro Médico Nacional de Occidente, 44340 Guadalajara, México

**Introducción**: El pseudotumor cerebri (PC), también conocido como hipertensión intracraneal idiopática, se caracteriza por síntomas de hipertensión intracraneal sin causa secundaria identificable. En adultos, la presentación clásica incluye cefalea, papiledema y alteraciones visuales; sin embargo, en la población pediátrica, la sintomatología puede variar considerablemente. Su diagnóstico es de exclusión y se requiere una presión de apertura de líquido cefalorraquídeo mayor a 28 cmH_2_O, así como estudios de neuroimagen sin presencia de lesiones ocupantes. El tratamiento, ya sea farmacológico o quirúrgico, tiene como objetivo reducir la presión intracraneal. **Objetivo**: Describir la presentación clínica de 4 pacientes pediátricos diagnosticados con PC en un hospital de tercer nivel. **Resumen Clínico del Caso Con Resultado de Estudios, Tratamiento Instaurado y Evolución**: Análisis retrospectivo de 4 casos de pacientes pediátricos diagnosticados con PC entre 2023 y 2025. La información fue recolectada a partir de los expedientes tanto físicos como electrónicos y revisión de la literatura en bases de datos como PubMed y UpToDate, además del uso de herramientas de inteligencia artificial como Consensus y Open Evidence. (1) Femenino, 16 años, con antecedente de derivación ventriculoperitoneal secundaria a trauma craneoencefálico con hemorragia subaracnoidea al año de edad. A los 15 años presentó cefalea escotomas, y baja visual progresiva en el ojo izquierdo, con papiledema. Fue tratada con a acetazolamida y colocación de válvula de derivación lumbo-peritoneal. (2) Masculino, 8 años, sano. Inició con otalgia, mirada desconjugada y cefalea. Se le administró acetazolamida durante 2 años sin recidiva. (3) Masculino, 12 años, sano, inicia con alteración del estado de alerta, cefalea intensa, vómitos, bradicardia sinusal asintomática, hipertensión arterial, parálisis del NC III, IV y VI, y alteraciones de la agudeza visual, manejado con VDVP. (4) Femenino, 15 años, sana, con cefalea intensa, fotofobia, náuseas, escotomas, y edema de papila, manejada con acetazolamida y triamcinolona transeptal. **Conclusiones**: En los 4 pacientes se corroboró el diagnóstico con presión de apertura mayor a 28 mmH_2_O. El PC presenta diversas manifestaciones clínicas en la población pediátrica, por lo que es crucial considerar este diagnóstico, aunque su incidencia sea baja.

**Palabras Clave:**

pseudotumor cerebri; hipertensión intracraneal idiopática; pediatría

**Importancia Serológica en Abordaje de Neuritis Óptica de Debut Seronegativo**

Sobrevilla Ponce Anali Guadalupe^1^, Cantú Salinas Adriana Carlota^1^, Carrión García Ana Luisa^1^, Vázquez Fuentes Salvador^1^, Chávez Luévanos Beatriz Eugenia^1^

^1^Hospital Universitario Dr. José Eleuterio González, 64460 Monterrey, México

**Introducción**: La neuritis óptica seronegativa en niños es un trastorno desmielinizante del Sistema nervioso central en el cual la inflamación del nervio óptico ocurre sin la presencia de anticuerpos comunes asociados, según criterios actuales, se clasifica en Neuritis óptica posible: se basa únicamente en características clínicas como dolor ocular y pérdida de la visión y Neuritis óptica definitiva: la cual se confirma con estudios paraclínicos los cuales podrían ser RMN cerebral u orbitaria, análisis de líquido cefalorraquídeo, detección de anticuerpos específicos AQP4 o MOG; al ser seronegativa dificulta identificar la causa y hace importante el seguimiento a largo plazo, sin embargo una de sus etiologías es la enfermedad asociada a la glicoproteína de oligodendrocitos de mielina (MOGAD) en donde anticuerpos dirigidos contra MOG inducen una respuesta inflamatoria que daña la mielina con un espectro cínico amplio y variable, abarcando neuritis óptica, mielitis transversa, encefalomielitis diseminada aguda y encefalitis. El tratamiento se divide en agudo y de mantenimiento, siendo uno de los más utilizados en el tratamiento de mantenimiento el rituximab. **Objetivo**: Presentar características clínicas y paraclínicas de paciente con neuritis óptica y la importancia del seguimiento evolutivo para la detección etiológica. **Resumen Clínico del Caso Con Resultado de Estudios, Tratamiento Instaurado y Evolución**: Femenina de 13 años, inició en enero 2023 con cuadro de visión borrosa hasta llegar a amaurosis y dolor ocular, se realizó diagnóstico de neuritis óptica ameritando hospitalización por 10 días, se abordó con RMN y OCT. La punción lumbar con parámetros normales, bandas oligoclonales negativas, antiaquaporina 4 (AQP4): negativa, ant-MOG negativos, se trató con metilprednisolona 1 gr por 5 días con mejoría del cuadro clínico y una duración del evento de dos semanas, egresando sin tratamiento. El 11 03 2025 presenta segundo evento de neuritis óptica, con dolor ocular izquierdo intermitente, agravado a la abducción y aducción; agregándose visión borrosa, discromatopsias, atrofia de nervio óptico bilateral; así como disminución del reflejo de acomodación y amenaza, agudeza visual OD 20/25, OI AV 20/25, Ishihara 5/16 en ojo derecho y 6/16 en ojo izquierdo. Se realizó RMN reportando nervio óptico bilateral con hiperintensidad, derecho con diámetro de 3,7 mm e izquierdo de 3,6 mm, de trayecto y morfología conservados, con reforzamiento difuso al contraste en segmentos ocular, intraorbitario y cisternal posterior, y restricción en difusión. Potenciales evocados visuales con alteración en N75 OD, OCT con datos compatibles con edema. Anti MOG en suero Positivo por técnica de Células vivas. Se inició tratamiento de mantenimiento con rituximab, 375 mg/m^2^ dosis única, 1er dosis, con seguimiento ambulatorio sin nuevas recaídas. **Discusion y Conclusiones**: La prevalencia de seroconversión en neuritis óptica seronegativa se sitúa en torno al 5–20% dependiente del contexto clínico. Ante una neuritis óptica seronegativa, a pesar de las recomendaciones diagnósticas de las nuevas guías, consideramos valioso el seguimiento serológico en caso de recaída, que permitirá la identificación y definición diagnóstica, así como un tratamiento orientado; además de brincar importante información pronóstica.

**Palabras Clave:**

MOGAD; neuritis óptica seronegativa; seroconversión y seguimiento

**Síndrome Goldenhar Asociado a Mosaicismo de la Trisomia 9, Características Clínico-Evolutivas: Reporte de Caso**

Sánchez Martínez Lumi Atzcalli^1^, Rivera Rodríguez Lidia Tania^1^, Ruíz Ferreira María Soledad^1^, Espinosa Zacarias Juan Pedro^1^

^1^Hospital Regional Lic. Adolfo Lopez Mateos, 01030 Ciudad de México, México

**Introducción**: El síndrome de Goldenhar, también conocido como síndrome óculo-aurícula-vertebral, es una anomalía innata. En el 85% de los casos la afectación facial es unilateral, siendo frecuente la afectación del lado derecho. En raras ocasiones, la afectación es bilateral. Su etiología exacta aún se desconoce. Se han identificado diversas anomalías cromosómicas. Estas incluyen: Mosaicismo de la trisomía 7, 9 y 22. Se han reportado casos de retraso global del desarrollo. Las malformaciones sistémicas más comunes incluyen anomalías del oído (100%), seguidas de afectación ocular (72%), vertebral (67%), anomalías del sistema nervioso central (50%) y cardiopatías congénitas (33%). Esta fórmula establece la presencia de 46 cromosomas, incluyendo el par de cromosomas sexuales XX, define la presencia de una translocación entre los cromosomas 9 y 21, la presencia de un iso cromosoma del brazo corto (p) del 9 y la ausencia de un cromosoma 21 libre. **Objetivo**: Describir el caso clínico de un paciente pediátrico con este síndrome asociado a cromosopatia. **Resumen Clínico del Caso Con Resultado de Estudios, Tratamiento Instaurado y Evolución**: Femenino de 4 años 7 meses, sin antecedentes familiares relevantes. Se obtiene producto masculino a las 34,5 semanas de gestación vía abdominal, bajo anestesia general, debido a preeclampsia con datos de severidad, peso 1800 g y talla 44 cm, APGAR 7/9, al nacimiento presenta microtia izquierda, hepatomegalia y laringomalacia posteriormente resuelta con traqueostomía, gastrostomía y funduplicatura. Comunicación interventricular desalineada, persistencia de conducto arterioso, foramen oval permeable corregido por cateterismo cardíaco a los 4 meses con 2 paros cardiorrespiratorios. Retinopatía del prematuro estadio 2 zona III ojo derecho/ estadio 1 zona III ojo izquierdo. Hipoplasia vertebral. Hipotiroidismo primario, IGF1 negativo. Epilepsia de componente motor tónico clónicas diagnosticada a los 2 meses tratada con Levetiracetam. Neurodesarrollo con retraso global. **Conclusiones**: Este caso clínico reportado con mosaicismo del cromosoma 9, dentro de las múltiples anomalías, existen pocos casos reportados con esta asociación de los cuales no se conoce la evolución de los mismos, la mayoría se describen en etapas avanzadas, con mal pronóstico. A pesar de ello, se dio seguimiento por 3 años, con buena evolución.

**Palabras Clave:**

síndrome Goldenhar; mosaicismo trisomía 9; epilepsia; parálisis cerebral

**Síndrome de Leigh Imitando la Presentación de una Encefalomielitis Diseminada Aguda (EMDA) en un Lactante**

Delgado Ramos Claudia Vanessa^1^, Santiago Gómez José Manuel^1^, León-Reyes Lluvia Itzel^1^

^1^Centro Médico Nacional 20 de Noviembre, 03104 Ciudad de México, México

**Introducción**: Tanto la encefalomielitis diseminada aguda (EMDA) como el síndrome de Leigh presentan un inicio agudo caracterizado por encefalopatía no explicada por causas infecciosas, o enfermedades sistémicas, pudiendo ambas entidades afectar la sustancia blanca, incluso con involucro del tronco encefálico y médula espinal en el caso de EMDA, y con probable involucro adicional de los ganglios basales y lesiones generalmente simétricas en el Síndrome de Leigh. Dentro del abordaje diferencial del EMDA de curso inesperado están los trastornos metabólicos. **Objetivo**: Presentación de un caso de síndrome de Leigh que debutó clínicamente y por neuroimagen con un cuadro compatible con EMDA con compromiso metabólico evidenciado a lo largo de su evolución. **Resumen Clínico del Caso Con Resultado de Estudios, Tratamiento Instaurado y Evolución**: Masculino de 2 años con antecedente de prematurez e hipoxia perinatal, diagnosticado con una anomalía de Ebstein Carpenter e hipoacusia que cursa con padecimiento subagudo caracterizado por fiebre, crisis, deterioro neurológico y estado epiléptico superrefractario cuya resonancia (IRM) mostró en T2FLAIR múltiples áreas hiperintensas difusas cortico-subcorticales localizadas en regiones occipital izquierda, frontal derecha y parietal bilateral de predominio izquierdo así como periventriculares y mesencefálicas. El EEG con actividad epileptiforme focal frontocentrotemporal derecha. Se descarta proceso infeccioso e inmunológico. Presenta evolución tórpida. En el seguimiento se identifica hiperamonemia e hiperlactatemia con resto de panel metabólico normal. Recibió tratamiento con biotina, coenzima Q10, levocarnitina, tiamina, cobalamina con mejoría clínica. Durante el episodio agudo presentó arritmias cardiacas que cedieron posteriormente. Actualmente con retraso del neurodesarrollo, epilepsia y movimientos hipercinéticos. Resultado de exoma con mutación de NDUFV1: c.232G>A (p. Gly78Ser) implicado en el complejo I de la cadena respiratoria mitocondrial asociada a su vez a Síndrome de Leigh. **Conclusiones**: En concordancia con la literatura consultada, el espectro del síndrome de Leigh es un trastorno neurodegenerativo, cuyas descompensaciones son detonadas frente a situaciones de aumento en la demanda metabólica, que debe de tomarse en cuenta en el abordaje etiológico de otras patologías de presentación similar, pero con diferente tratamiento y evolución.

**Palabras Clave:**

encefalomielitis diseminada aguda; síndrome de Leigh; trastorno mitocondrial

**Importancia de la Interpretracion de las Mutaciones de los Canales de Sodio. Presentacion de una Mutación en SCN8A (C1855-1863 DUp GGTACAGC) Gly619:ser621Dup, con Debut Catastrofico**

Mercado Silva Miguel Francisco^1^, Jimenez Arredondo Ramon Ernesto^1^

^1^Hospital Ángeles del Carmen, Medicina Privada, 44130 Guadalajara, México

**Introducción**: La encefalopatía epiléptica (EE) por mutación en el gen *SCN8A* que codifica subunidad del canal iónico de sodio es patología muy poco frecuente, infradiagnosticada y asociada a fenotipo de espectro amplio como una severa encefalopatía, con crisis epilépticas intratables de inicio al comienzo de la vida. En el banco de reportes mundiales son 23 casos reportados de pacientes con EE y mutaciones causantes de enfermedad en SCN8A. **Objetivo**: Presentar reporte de caso de paciente con mutación en gen *SCN8A* donde la determinación Genómica permitió su tratamiento adecuado. **Resumen Clínico del Caso Con Resultado de Estudios, Tratamiento Instaurado y Evolución**: Masculino 13 años de edad, sin epilepsia familiar, producto del 2° embarazo, sin hipoxia neonatal, desarrollo Psicomotor normal, escolaridad preescolar con buen desempeño hasta los 4 años. Inició 4 años de edad con estado epiléptico refractario a tratamiento, TAC y RMNC de cráneo normales, LCR para panel viral negativo. EEG brotes de punta onda lenta operculares bisincronícas intermitentes. Recibió tratamiento con DFH, Fenobarbital, vigabatrina, Oxcarbazepina, levetiracetam, Acido Valproico, topiramato, lamotrigina, acetazolamida, flunarizina, clonazepam, Lorazepam, Canabidiol, primidona, brivaracetam, lacosamida, Inmunoglobulinas intravenosas. Se colocó Estimulador del nervio Vago (VNC) con persistencia de 5 a 120 crisis al mes, con variantes como parcial motora izquierda facial, espasmos tónicos, clónicas generalizadas y astáticas. Se realizó perfil Genético encontrándose Mutación en SCN8A (C1855-1863 DUp GGTACAGC) Gly619: ser621Dup, modificando el tratamiento farmacológico aa Oxcarbamazepinay Valproato de magnesio con un control completo y únicamente descontrol con fiebre. **Conclusiones**: En nuestro caso se identificó mutación con duplicación in-frame repitiéndose una pequeña porción del gen (nucleótidos 1855 a 1863): GGTACAGC, no cambia marco de lectura, pero duplica tres aminoácidos en proteína, desde glicina 619 (Gly619) hasta serina 621 (Ser621). Estas duplicaciones in-frame en SCN8A pueden alterar la apertura/cierre del canal, provocando hiperexcitabilidad neuronal, favoreciendo epilepsia de difícil control por una ganancia de función.

**Palabras Clave:**

encefalopatia epileptica; mutacion SCN8A

**Neuromodulación con Estimulador Profundo en 2 Pacientes Pediátricos con Epilepsia Farmacorresistente Posterior a Fracaso de Cirugia Resectiva**

Flores García Karla Wendoline^1^, Villarreal García Lourdes Verónica^1^, Roldán Gutiérrez Martín Iván^1^, León Reyes Lluvia Itzel^1^, Rodríguez García Verónica^1^, Venta Sobero José Antonio^1^

^1^Centro Médico Nacional “20 de Noviembre”, ISSSTE, 03229 Ciudad de México, México

**Introducción**: Ante los casos de epilepsia farmacorresistente (EFR), especialmente en displasias corticales focales (DCF), la cirugía personalizada toma un papel principal llevando a un mejor desarrollo cognitivo e integración social en los pacientes. Revisiones recientes reportan hasta un 79% de control de crisis post cirugía en los centros con más experiencia. El 10% sin embargo, experimenta recaída tras un periodo libre de crisis de 2 años. Dentro de la patogénesis de la red de crisis epilépticas, la conectividad talamocortical se encuentra como mecanismo de propagación en epilepsias generalizadas y focales. La estimulación talámica se torna en una opción en estos casos, sin embargo, existe una escasez de datos sobre los resultados en pacientes pediátricos. **Objetivo**: Describir la experiencia con estimulador talámico anterior en dos pacientes pediátricos con diagnóstico de EFR. **Resumen Clínico del Caso Con Resultado de Estudios, Tratamiento Instaurado y Evolución**: Caso 1: paciente de 13 años con DCF frontal izquierda y frontotemporal derecha asociada a Hipomelanosis de Ito con frecuencia de hasta 75 crisis por día y múltiples estados epilépticos. En el 2022 se realizó lobectomía temporal anterior con amigdalo-hipocampectomía derecha, con reducción del 30% de crisis aproximadamente y persistencia de estados epilépticos por lo que en el 2023 se decide colocación de estimulador talámico anterior, con frecuencia actual de 1–4 crisis por día y remisión de estados epilépticos. Caso 2: paciente de 17 años con diagnóstico de EFR secundaria a DCF temporoparietal con 60 crisis al día. En el 2012 se realizó lobectomía temporal anterior derecha con reducción del 50% de crisis, pero siendo estas altamente discapacitantes, por lo que en el 2024 se decide colocación de estimulador talámico anterior, mostrando posteriormente 3–4 crisis por semana y mejor calidad de vida. **Conclusiones**: Se evidenció una mejoría significativa en el patrón de frecuencia y duración de las crisis, así como ausencia de estados epilépticos.

**Palabras Clave:**

epilepsia farmacorresistente; estimulador cerebral profundo; estimulador talámico anterior; displasia cortical focal

**Tratamientos Combinados y Secuenciales Para Atrofia Muscular Espinal: Primera Experiencia con Onasemnogén Abeparvovec (Zolgensma®) Más Risdiplam (Everysdi®) en un Hospital Privado en México**

Infante Cantú José Antonio^1^, Rodríguez Rivera Sofía Lucila^1^, Martínez Héctor R.^1^, Caro Osorio Enrique^1^

^1^Instituto de Neurología y Neurocirugía, Hospital Zambrano Hellion, TecSalud, Tecnológico de Monterrey, 66278, San Pedro Garza García, México

**Introducción**: La atrofia muscular espinal (AME) es una enfermedad neuromuscular autosómica recesiva [mutación homocigota gen *SMN1* brazo largo cromosoma 5 (5q11.1–13.3)] con degeneración de motoneuronas alfa asta anterior médula espinal, con debilidad muscular progresiva y alto riesgo de soporte ventilatorio. Zolgensma® (terapia génica) contiene onasemnogen abeparvovec, con copia funcional gen *SMN*. Risdiplam es un modificador del empalme del pre-ARNm de SMN2. **Objetivo**: Evaluar la eficacia en dos pacientes con AME en quienes se aplicó terapia génica con onasemnogen abeparvovec (Zolgensma®) con posterior tratamiento combinado con Risdiplam (Everysdi®). **Resumen Clínico del Caso Con Resultado de Estudios, Tratamiento Instaurado y Evolución**: Caso clínico 1 y 2. Femenino 4 meses y masculino 6 meses de edad, productos de embarazos normoevolutivos, parto eutócico, con hipotonía y retraso del neurodesarrollo al mes de edad y a los 4 meses, respectivamente. Fasciculaciones linguales, hipotonía y debilidad proximal 4 extremidades 3/5, arreflexia. Disociación toraco-abdominal. Pruebas genéticas MLPA: 0 y 0 copias gen *SMN1* exón 7; 1 y 1 copia gen *SMN1* exón 8; 2 y 3 copias gen *SMN2*. Diagnóstico: atrofia muscular espinal tipo 1 (a los cuatro y cinco meses de edad). Anticuerpos anti-AAV9 (negativos <1:50). Zolgensma® se aplicó a los cinco y seis meses de edad, dosis única IV 1,1 × 1014 genomas virales/kg. Escala Chop Intend: 37 (grado moderado) antes del tratamiento con Zolgensma®; 42 y 45 después del tratamiento con Zolgensma®, con mejoría en fuerza muscular y control cefálico. Seis meses después se inició Risdiplam vía oral 0,20 mg/kg con mejoría en Escala Chop Intend a 46 y 49, logrando sedestación con apoyo. Prueba estadística Friedman: la escala motora es diferente según el tratamiento (Zolgensma® vs. Zolgensma® más Risdiplam), con significancia <0,05. **Conclusiones**: Los pacientes presentaron mejoría en escalas motoras y no requirieron uso de ventilación mecánica con terapia combinada y secuencial de Zolgensma® más Risdiplam. En la literatura se menciona que es posible utilizar terapias combinadas y secuenciales. Aún se requieren más estudios para evaluar la eficacia y seguridad en el uso inicial de terapias combinadas, siendo la primera experiencia en hospital privado en México.

**Palabras Clave:**

chop intend; atrofia muscular espinal; onasemnogen abeparvovec; risdiplam

**Discapacidad Intelectual Ligada al Cromosoma X Por Variante Patogénica del Gen *GRIA3* Asociada a Trastorno Mioclónico y Epilepsia**

Narváez González Diana Elisa^1^, Rosas Ayala Ana Elia^1^, Palacios Ponce José Luis^1^, Cantú Salinas Adriana Carlota^1^, Carrión García Ana Luisa^1^, Vázquez Fuentes Salvador^1^, De la Garza Pineda Oscar^1^, Chávez Luévanos Beatriz Eugenia^1^

^1^Servicio de Neurología Pediátrica, Hospital Universitario “Dr. José Eleuterio González”, Universidad Autónoma de Nuevo León, 64460 Monterrey, México

**Introducción**: El gen *GRIA3* localizado en el cromosoma Xq15 codifica la subunidad 3 del receptor de glutamato tipo AMPA (AMPAR) responsable de las corrientes postsinápticas excitatorias rápidas, actuando en el desarrollo cerebral, comunicación neuronal, procesos de aprendizaje y memoria, a través de la plasticidad sináptica. **Objetivo**: Describir la evolución clínica y abordaje terapéutico en paciente con mutación genética poco frecuente. **Resumen Clínico del Caso Con Resultado de Estudios, Tratamiento Instaurado y Evolución**: Masculino de 6 años de edad, producto de la primera gesta de embarazo normoevolutivo. Nace a término, peso y talla adecuados, APGAR 5/8, requiriendo maniobras avanzadas de reanimación. A los 2 años de vida se detecta retraso global del neurodesarrollo, a la exploración física con escaso sostén cefálico, hiperreflexia e hipertonía en las 4 extremidades. A los 36 meses debuta con crisis focales alternadas con movimientos tipo clónicos y mioclónicos, agregándose diplejía y datos clínicos compatibles con parálisis cerebral infantil. Durante los primeros 5 años de vida, ninguno de los electroencefalogramas (EEG) realizados mostraba actividad ictal, posteriormente a los 6 años, se detecta actividad focal en región temporal izquierda. Los movimientos rápidamente se volvieron más frecuentes y resistentes a fármacos, manteniendo un estado mioclónico persistente multitratado sin mejoría. Se valora por primera vez en nuestro servicio, observando lenguaje limitado, alteración del nervio craneal accesorio, fuerza muscular 3/5 superior, 2/5 inferior, incremento del tono muscular, reflejos osteotendinosos +++ hemicuerpo izquierdo, imposibilidad para la marcha y alteraciones en la coordinación. En resonancia magnética, se observa aumento en la amplitud de astas frontales, así como disminución de núcleos caudados, cuerpo calloso adelgazado, megacisterna magna y silla turca morfología en J. Es enviado a Genética donde se obtiene ADN con resultado positivo de variante genómica GRIA3, se ajustan anticrisis y se inicia Piracetam a dosis bajas, con mejoría en las primeras semanas de administración. **Conclusiones**: No existe un fenotipo específico para la mutación del GRIA3, ya que el número de pacientes documentados es muy limitado, no obstante, la asociación al trastorno mioclónico se ha evidenciad, pese a que aún se encuentra en estudio, el Piracetam actúo como opción terapéutica adecuada en mejoría clínica del paciente.

**Palabras Clave:**

mutación; epilepsia; mioclónico; Piracetam

**Neurolupus Como Primera Manifestación de Lupus Eritematoso Sistémico de Inicio en la Infancia: Experiencia Clínica en el Hospital Regional de Alta Especialidad Dr. Ignacio Morones Prieto**

Rodolfo Enrique Rangel Ayon^1^, Antonio Bravo Oro^1^, Jorge Luis García Ramírez^1^

^1^Hospital Regional de Alta Especialidad “Dr. Ignacio Morones Prieto”, 78210 San Luis Potosí, México

**Antecedentes**: El lupus eritematoso sistémico (LES) de inicio en la infancia es una enfermedad autoinmune compleja con una prevalencia estimada de 1–25 por cada 100.000 niños. El neurolupus pediátrico, como manifestación neurológica del LES, representa una de las formas más graves y menos comprendidas. Su espectro clínico incluye crisis convulsivas, eventos vasculares cerebrales, ataxia, corea, neuropatías periféricas y alteraciones cognitivas. En hasta un 40% de los casos, el compromiso neurológico puede preceder al diagnóstico formal de LES, lo que resalta la importancia de un enfoque clínico estructurado y multidisciplinario. **Objetivo**: Describir las manifestaciones clínicas y de laboratorio en una serie de pacientes pediátricos con LES cuya manifestación inicial fue neurológica, destacando la relevancia del diagnóstico temprano y abordaje integral. **Material y Métodos:** Estudio observacional de una serie de 8 casos pediátricos con diagnóstico de LES, todos cumpliendo criterios SLICC, cuya primera manifestación fue neurológica. **Resultados**: Se analizaron 8 pacientes con diagnóstico de LES con manifestaciones neurológicas como forma de presentación inicial. La relación F:M fue de 6:2, con edad promedio de inicio de 10,4 años. Las manifestaciones neurológicas más frecuentes incluyeron: cefalea (50%), hemiparesia (37,5%), crisis convulsivas, ataxia, disartria y síntomas neuropsiquiátricos (25%). También se documentaron signos focales como nistagmo, afecciones pares craneales, corea y alteración estado alerta. Predominaron los EVC isquémicos en el contexto de vasculitis SNC, seguidos de ataxia cerebelosa, corea lúpico y crisis convulsivas sintomáticas agudas. Todos los pacientes presentaron ANA positivos; una parte presentó anticuerpos antifosfolípidos. **Conclusiones**: El neurolupus puede ser la manifestación inicial del LES en población pediátrica y cursar con síntomas neurológicos graves. El reconocimiento precoz, el uso adecuado de estudios inmunológicos y de neuroimagen, así como el inicio oportuno del tratamiento inmunosupresor, son fundamentales para prevenir secuelas. La experiencia de nuestro centro enfatiza el valor del abordaje multidisciplinario y del seguimiento neurológico estrecho en estos casos.

**Palabras Clave:**

lupus eritematoso sistemico (LES); evento vascular cerebral (EVC)

**Espectro Clínico de Epilepsias Relacionadas con Mutaciones en Canales de Sodio en un Centro de Atención Terciaria: Heterogeneidad Genotípica-Fenotípica**

Rodolfo Enrique Rangel Ayon^1^, Antonio Bravo Oro^1^, José de Jesús Vázquez Montante^1^, Jorge Luis García Ramírez^1^

^1^Hospital Regional de Alta Especialidad “Dr. Ignacio Morones Prieto”, 78210 San Luis Potosí, México

**Antecedentes**: Mutaciones en los canales de sodio dependientes de voltaje en los últimos años se han asociado a epilepsias del desarrollo, encefalopatías epilépticas y trastornos del ritmo cardíaco. Mutaciones en *SCN1A*, *SCN2A*, *SCN3A* y *SCN5A* alteran la excitabilidad neuronal y se asocian a epilepsias de inicio temprano, discapacidad intelectual y otros trastornos del neurodesarrollo desde edades tempranas. **Objetivo**: Evaluar y describir la correlación entre genotipo y fenotipo de pacientes con variantes patogénicas en canales de sodio en nuestra institución. **Material y Métodos**: Se describe la heterogeneidad genotípica-fenotípica de 7 pacientes no relacionados con variantes patogénicas en canales de sodio. **Resultados**: Se analizaron un total de 7 pacientes pediátricos no relacionados. La edad promedio de inicio de las crisis fue de 3,2 años; 6 fueron varones. Se identificaron siete variantes patogénicas en 4 genes distintos: una en *SCN1A*, 4 en *SCN2A*, una en *SCN3A* y una en *SCN5A*. De ellas, dos variantes fueron nuevas y no reportadas previamente: *SCN3A* c.2974C>T (p. Leu992Phe) y *SCN5A* c.101G>A (p. Arg34His). En cuanto a diagnóstico clínico, un paciente presentó síndrome de crisis febriles plus (GEFS+), cuatro presentaron DEE y dos epilepsias farmacorresistentes sin un síndrome específico. 6 de los 7 pacientes tenían discapacidad intelectual asociada; únicamente el caso con GEFS+ mostró neurodesarrollo preservado. **Conclusiones**: Este estudio destaca la heterogeneidad clínica de las epilepsias relacionadas con canalopatías por variantes patogénicas en SCN. El hallazgo de estas variantes, especialmente en casos de epilepsia de difícil control o inicio temprano, es fundamental para establecer un diagnóstico etiológico preciso y orientar a un mejor tratamiento. Además, las variantes novedosas descritas en nuestro estudio enriquecen el conocimiento genético actual y podrían tener implicaciones futuras en terapias dirigidas y farmacogenómica.

**Palabras Clave:**

canal de sodio dependiente de voltaje (SCN); crisis febriles plus (+) (GEFS +); encefalopatía epiléptica y del desarrollo (DEE)

**Impacto del Uso de Pantallas Sobre el Neurodesarrollo en Menores de 6 Años**

Diana L. Gutiérrez-Ramírez^1^, Sarahé A. Acuña-Mendoza^2^, Carlos Sánchez-García^2^, Adriana C. Cantú-Salinas^1^, Beatriz E. Chavez-Luevanos^1^

^1^Neurología Pediátrica, Hospital Universitario “Dr. José Eleuterio González”, 64460 Monterrey, Nuevo León, México

^2^Pediatría, Hospital del Niño “Federico Gómez Santos”, 25000 Saltillo, Coahuila, México

**Antecedentes**: En México la relación de trastornos del neurodesarrollo (ND) con la exposición a pantallas ha sido pobremente estudiada. La evaluación del ND se realiza con la Evaluación del Desarrollo Infantil (EDI). **Objetivo**: Determinar el impacto del uso de pantallas en el ND en niños menores de 6 años. **Material y Métodos**: Estudio transversal, descriptivo, observacional, realizado en el mes de octubre del año 2023, en el Hospital del Niño de Saltillo, Coahuila. Con muestra total: 115 pacientes, 5 con criterios de exclusión (diagnóstico de trastorno del ND, antecedente de prematurez extrema sin rehabilitación o pacientes con síndromes que afectan ND). Se realizó prueba EDI según el grupo de edad correspondiente, además de encuesta sobre uso de pantallas. Se analizó con frecuencias, T de student y prueba exacta de Fisher; las variables estadísticamente significativas se sometieron a regresión logística univariada y multivariada. La exposición a pantallas en horas se analizó con ANOVA. **Resultados**: 110 pacientes, 46,4% (n = 51) fueron mujeres y 53,6% (n = 59) fueron hombres. 81,8% (n = 90) tuvieron contacto temprano a pantallas. Media en hrs de exposición de 2,56 con DS de 1,52; mediana de 2, rango de 0 a 8 hr. 59,1% (n = 65) con resultado verde (normal); 15,5% (n = 17) amarillo (probable rezago en el ND); 25,5% (n = 28) con resultado rojo (probable retraso en el ND). Áreas con mayor afección: lenguaje (20%, n = 22), conocimiento (10,9%, n = 12) y motor fino (10%, n = 11). El grupo 13 de evaluación (Edad: 25 a 36 meses) (*p* = 0,005, OR 5,61), nivel socioeconómico bajo (*p* = 0,007, OR 3,51) y horas de exposición a pantallas (*p* = 0,007, OR 1,57) obtuvieron significancia estadística. En cuanto a las horas de exposición de pantallas, se decidió realizar una comparación entre resultados verdes, amarillos y rojos, por medio de un análisis de la varianza de medidas repetidas. **Conclusiones**: La exposición temprana y excesiva a pantallas representa un riesgo para el ND del menor de 6 años, sobre todo entre los 25 y 36 meses de vida, con nivel socioeconómico bajo.

**Palabras Clave:**

neurodesarrollo; trastorno; pantallas

**Caracteristicas Clinicas, Electroencefalograficas, de Imagen E Histopatolgicas de Pacientes en Edad Pediátrica con Diagnóstico de Encefalitis de Rasmussen**

Gutiérrez Soto-Marisol^1^, Reyes Cuayahuitl-Araceli^1^, Rayo Mares-Jesús Darío^1^, Carlos Esteban Linares Franco^1^, Orozco Suárez Sandra^2^, López Gallegos-Gabriela Monserrat^1^

^1^Servicio de Neurología Pediátrica, Hospital de Pediatría CMN Siglo XXI, IMSS, 06720, Ciudad de México, México

^2^Unidad de Investigación Médica en Enfermedades Neurológicas, UMAE Hospital de Especialidades CMN Siglo XXI, IMSS, 06720 Ciudad de México, México

**Antecedentes**: La encefalitis de Rasmussen (ER) es una enfermedad que se manifiesta comúnmente en la infancia cuyas principales características incluyen estado epiléptico focal continuo, hemiplejia lentamente progresiva y discapacidad intelectual. La RNM de encéfalo muestra un agrandamiento unilateral del sistema ventricular y atrofia hemisférica ipsilateral. Los rasgos histopatológicos característicos son inflamación cortical, pérdida neuronal y gliosis confinada a un hemisferio cerebral. El tratamiento el quirúrgico en la mayoría de los casos. **Objetivo**: Describir la presentación clínica, electroencefalográficas, estudios de imagen e histopatológicas en pacientes con encefalitis de Rasmussen. **Material y Métodos**: Estudio transversal, con datos obtenidos a través de bases de datos e historias clínicas. **Resultados**: Se incluyeron 10 pacientes la edad más frecuente fue de 5 años, la mayoría del sexo masculino, tiempo de diagnóstico de 1–5 años. El 70% (n = 7) presentaron crisis focales motoras izquierdas, 20% (n = 2) derechas, 30% (n = 3) TCG, 30% (n = 3) crisis no motoras. En un 50% (n = 5) presentaron hemiparesia izquierda, 20% derecha. Al 70% (n = 7) se les realizó hemisferectomia anatómica derecha, 20% (n = 2) izquierda. 10% (n = 1) hemisferectomia funcional derecha + lesionectomía frontal derecha. Anticuerpos anti-Glu3 positivos en un 50% (n = 5). En RNM hemiatrofia hemisférica en un 10% (n = 1) lado derecho de predominio frontal. 10% (n = 1) atrofia cortico subcortical de predominio frontal bilateral + hiperdensidad en núcleos de la base. El 30% (n = 3) presentó hemianopsia homónima. En el reporte histopatológico se encontró en un 40% (n = 4) infiltrado linfocitario perivascular CD4++ microglía activa y un 20% (n = 2) infiltrado linfocitario perivascular CD8++ y pérdida neuronal. **Conclusiones**: La encefalitis de Rasmussen representa un reto terapéutico en la edad pediátrica, por caracterizarse por epilepsia focal resistente, deterioro neurológico progresivo y signos de disfunción hemisférica, requiere un alto índice de sospecha para permitir una intervención oportuna. La resonancia magnética cerebral seriada y el EEG son herramientas clave para su identificación. La hemisferectomía como lo fue en todos los pacientes presentados continúa siendo el tratamiento más eficaz para el control de crisis y la estabilización clínica en casos refractarios.

**Palabras Clave:**

encefalitis de Rasmussen; hemiparesia; hemisferectomia anatómica; anticuerpos anti-Glu3

**Comparación de la Respuesta Clínico-Terapéutica de Pacientes Con Síndrome de Opsoclono Mioclono Ataxia: Un Estudio de Cohorte**

Katia Moreno-Silva^1^, Alberto Ibarra-Delgado^1^, Miguel Ángel Rosas-Contreras^1^, Yael Caballero-Navarro^1^, Yereth Torres-Damián^1^, Flora Cebada López^1^, Daniela Méndez Fernández^1^, Verónica Lomán-Zúñiga^1^, Viridiana Cadena-Ramos^1^, Andrea Carolina Saldívar Santillán^1^

^1^Instituto Mexicano del Seguro Social UMAE Hospital General “Dr. Gaudencio González Garza”, Centro Médico Nacional La Raza, 02990 Ciudad de México, México

**Antecedentes**: El síndrome opsoclono-mioclono (SMO) es un trastorno inmunitario poco frecuente que se caracteriza por opsoclono, mioclonía, ataxia y cambios de comportamiento. En algunos casos puede estar asociado con la presencia de un tumor de la cresta neural principalmente neuroblastoma, ganglioneuroblastoma y ganglioneuroma. El pronóstico ha mejorado con el uso temprano de terapias combinadas. **Objetivo**: Comparar la respuesta clínica al tratamiento clásico Vs tratamiento actual. **Material y Métodos**: Se realizó un estudio observacional, analítico, ambispectivo y longitudinal de pacientes pediátricos con síndrome de opsoclono mioclono ataxia atendidos en la UMAE Hospital General Dr. Gaudencio González Garza. Se realizó un muestreo no probabilístico consecutivo donde se incluyeron a todos los pacientes atendidos en el periodo de tiempo comprendido de 2019 a 2025. Se empleó estadística descriptiva para las variables cualitativas con cálculo de frecuencias y porcentajes, en cuanto a las variables cuantitativas se utilizaron media y mediana de acuerdo al tipo de distribución de las variables. Se utilizaron las escalas de Mitchell y Pranzatelli para el seguimiento de los pacientes, con evaluaciones a los 3, 6, 9 y 12 meses de haber sido diagnosticado. **Resultados**: Se estudiaron un total de 12 pacientes de los cuales 42% fueron hombres y 58% mujeres en su mayoría menores de 6 años, observando una mediana inicial de 24 puntos en la escala de Pranzatelli y 14 en la escala de Mitchell, con mejoría significativa a los 3 meses después de iniciado el tratamiento, 3 de los pacientes no mejoraron tras la administración de inmunoglobulina y esteroide por lo que requirieron de rituximab. Solo en 2 casos se identificó una neoplasia asociada a la enfermedad, ambas recibieron tratamiento correspondiente. **Conclusiones**: Dentro de las primeras líneas de tratamiento se recomienda pulsos de esteroide sistémico con inmunoglobulina humana lo cual en la mayoría de los pacientes se logra una remisión de la enfermedad al año de edad, un pequeño porcentaje requieren manejo con rituximab. En nuestra población estudiada se observan datos similares a lo descrito en la literatura internacional con mejoría de los síntomas posterior a inicio de tratamiento dirigido.

**Palabras Clave:**

síndrome opsoclono-mioclono-ataxia; Pranzatelli; Mitchell; inmunoterapia

**Síndrome de Guillain-Barré con Afectación Bulbar 10 Años de Experiencia en el Instituto Nacional de Pediatría**

Medellín López Haideé Deyanira^1^, Hernández Antúnez Blanca Gloria^1,2^, Ruiz García Matilde^2^

^1^Instituto Nacional de Pediatría (INP), 04530 Ciudad de México, México

^2^Universidad Nacional Autónoma de México, 04510 Ciudad de México, México

**Antecedentes**: El síndrome de Guillain-Barré (SGB) es una polirradiculoneuropatía inflamatoria aguda inmunomediada. La presentación clásica no suele plantear un desafío diagnóstico, pero las manifestaciones atípicas representan un reto cuando no se las considera. Por lo que, este trabajo de investigación plantea la presentación clínica con afectación bulbar, con la finalidad de caracterizar esta variante. **Objetivo**: Describir la presentación clínica de los pacientes pediátricos con Síndrome de Guillain-Barré con afectación bulbar en el Instituto Nacional de Pediatría durante un periodo de 10 años. **Material y Métodos**: Se presentan los casos valorados en el Instituto Nacional de Pediatría con diagnóstico de SGB y afectación bulbar del 2014 al 2024. Estudio observacional, retrospectivo, descriptivo y analítico. **Resultados**: En nuestro trabajo de un total de 125 pacientes con diagnóstico de SGB, 42 pacientes (33,6%) presentaron afectación bulbar. El grupo etario más frecuente fueron los escolares (42,9%), adolescentes (33,3%), preescolares (21,4%) y lactantes (2,4%), con predominio de sexo masculino (71,4%), presentaba una infección respiratoria previa (38,1%) y gastrointestinal (28,6%). De las manifestaciones clínicas en orden de frecuencia presentadas fueron: dificultad respiratoria (54,8%), parálisis facial (52,4%), disfagia (42,9%) y oftalmoparesia (30,9%). De la escala Erasmus GBS Respiratory Insufficiency Score (EGRIS) 23 pacientes presentaron >5 puntos y requirieron ventilación mecánica invasiva el 45,2%. Presentaron disociación albumino citológica (73,8%), 25 pacientes fueron evaluados para anticuerpos anti-gangliósidos, de los cuales fueron positivos IgG anti-GT1a (28%), anti-GQ1b (24%), anti GD1b (12%). De las variantes neurofisiológicas la más frecuente fue la variante axonal motora (AMAN – 57,1%) seguida de polineuropatía desmielinizante aguda (AIDP – 21,4%). Dentro de las complicaciones asociadas a la hospitalización se reportó neumonía asociada a la ventilación mecánica como la más frecuente (45,2%), el requerimiento de traqueostomía (28,6%) y gastrostomía (19%). **Conclusiones**: Nuestra serie de casos amplía la comprensión del SGB con afectación bulbar. Invita a considerar las manifestaciones clínicas más frecuentes en estos pacientes, así como la utilidad de escalas de predicción clínica. Serológicamente, la presencia de anticuerpos IgG anti-GT1a, anti-GQ1b y anti GD1b predice y contribuye a la enfermedad por lo que habría que tomarlo en cuenta al momento del diagnóstico.

**Palabras Clave:**

síndrome de Guillain-Barré; polirradiculoneuropatía; afectación bulbar; oftalmoparesia; parálisis facial

**Análisis Clínico y Epidemiológico de Pacientes con Epilepsia del Hospital General Pachuca**

Cruz Alaguna Mariana Ivonne^1^, Palomares Valdez Eduardo^1^

^1^Hospital General Pachuca, 42078 Pachuca de Soto, México

**Antecedentes**: La epilepsia es una de las enfermedades neurológicas más prevalentes en edad pediátrica, con implicaciones clínicas, familiares y sociales. La caracterización de los pacientes a nivel local permite orientar estrategias terapéuticas y de seguimiento más efectivas para mejorar su calidad de vida. **Objetivo**: Describir las características clínicas y epidemiológicas de pacientes pediátricos con diagnóstico de epilepsia atendidos en el área de consulta externa del Hospital General de Pachuca en el periodo comprendido entre enero y abril del 2025. **Material y Métodos**: Estudio observacional, descriptivo y retrospectivo. Se revisaron 300 expedientes clínicos de pacientes con epilepsia. Se recolectaron variables sociodemográficas (edad, sexo), clínicas (tipo de crisis, antecedente de crisis febriles, comorbilidades) y terapéuticas (efectos secundarios). El análisis se realizó con estadística descriptiva. **Resultados**: La edad promedio fue de 9,8 años (rango: 1 a 19 años). El 53% fueron de sexo masculino (160 pacientes y 42,7% sexo femenino (128 pacientes). Dentro del tipo de crisis, las crisis tónico clónico-generalizadas fue la más frecuente en un 98% (294 pacientes), las crisis de ausencia en 1,3% (4 pacientes) y focales en 0,7% (2 pacientes). El 89% sin antecedentes de crisis febriles (268 pacientes) y solo el 11% con antecedentes positivos (32 pacientes). La comorbilidad más frecuente fue la discapacidad intelectual, presente en diversas formas: severa (6 casos), moderada (3), leve (3). Solo el 1,6% (5 pacientes) presento algún efecto secundario farmacológico. El 98,8% de los pacientes recibían tratamiento farmacológico. Pacientes sin tratamiento: 2 (0,7%). **Conclusiones**: La mayoría de los pacientes pediátricos con epilepsia atendidos en el Hospital General de Pachuca presentan crisis tónico-clónica-generalizadas, presentando comorbilidades neurológicas asociadas, especialmente discapacidad intelectual. Estos hallazgos subrayan la importancia de un enfoque multidisciplinario para su atención y seguimiento.

**Palabras Clave:**

epilepsia; epidemiología; pediatría; discapacidad intelectual

**Descripción Clinico-Etiológica del Síndrome de Guillain-Barré en la Unidad Médica de Alta Especialidad, Hospital de Pediatría CMNSXXI**

López Gallegos-Gabriela Monserrat^1^, Castro Ruiz-Alfredo^1^, Reyes Cuayahuitl-Araceli^1^, Rayo Mares-Jesús Darío^1^

^1^Hospital de Pediatría CMN Siglo XXI, IMSS, 06720 Ciudad de México, México

**Antecedentes**: El síndrome de Guillain-Barré es la principal causa de parálisis flácida aguda. Se suele presentar un antecedente de infección respiratoria o gastrointestinal; generalmente se manifiesta con debilidad en extremidades de inicio distal y progresión ascendente. La enfermedad puede progresar hasta alcanzar una fase de meseta, en la que 20–30% desarrollan complicaciones, afectando el mantenimiento de la vía aérea y desencadenando insuficiencia respiratoria. El tratamiento se basa en la gammaglobulina intravenosa. La detección oportuna permite establecer un diagnóstico que influye en la toma de decisiones; es por ello que con el presente trabajo se propone evaluar las manifestaciones clínicas y la etiología de la enfermedad en pacientes pediátricos. **Objetivo**: Describir la etiología de los pacientes pediátricos con síndrome de Guillain Barré y sus manifestaciones clínicas en la UMAE Pediatría CMN Siglo XXI. **Material y Métodos**: Estudio transversal descriptivo, con datos obtenidos a través de bases de datos e historias clínicas, que se llevó a cabo en la Unidad Médica de Alta Especialidad de Pediatría, Centro Médico Nacional Siglo XXI en el periodo comprendido del 2015 al 2022. **Resultados**: Se incluyeron 53 pacientes. Se observó predominio de la patología en adolescentes (49%), sin diferencias significativas respecto al sexo. 32 pacientes presentaron antecedentes de proceso infeccioso, de los cuales el 59% correspondieron a infecciones gastrointestinales y 38% infecciones de vías respiratorias. Se encontró prueba positiva de IgG para CMV en un paciente, un caso de aislamiento de Borrelia en el LCR y un caso asociado a infección por SARS-CoV-2. Se observó que la enfermedad cursó predominantemente con debilidad simétrica (84%, n = 45), arreflexia universal (94%, n = 50) y progresión de la debilidad (92%, n = 49). Respecto al tiempo de recuperación, se obtuvo un promedio de 18 días, con una estancia hospitalaria promedio de 29 días. Se reportó la defunción de 3 pacientes. **Conclusiones**: La mayoría de los casos presentan asociación con algún antecedente infeccioso, no en todos se identificó el agente específico, situación que es importante para la instauración de medidas preventivas o de tratamiento dirigido.

**Palabras Clave:**

Guillain-Barré; proceso infeccioso; arreflexia; debilidad

